# Azide–alkyne cycloaddition (click) reaction in biomass-derived solvent Cyrene^TM^ under one-pot conditions

**DOI:** 10.3762/bjoc.21.117

**Published:** 2025-07-30

**Authors:** Zoltán Medgyesi, László T Mika

**Affiliations:** 1 Department of Chemical and Environmental Process Engineering, Faculty of Chemical Technology and Biotechnology, Budapest University of Technology and Economics, Műegyetem rkp. 3., H-1111, Budapest, Hungary

**Keywords:** alternative solvent, click chemistry, cycloaddition, Cyrene^TM^, 1,2,3-triazoles

## Abstract

It was demonstrated that Cyrene^TM^, as a biomass-originated polar aprotic solvent, could be utilized as an alternative reaction medium for one-pot copper(I)-catalyzed azide–alkyne cycloaddition (click or CuAAC) reactions, for the synthesis of various 1,2,3-triazoles under mild conditions. Nineteen products involving *N*-substituted-4-phenyl-1*H*-1,2,3- and 1-allyl-4-substituted-1*H*-1,2,3-triazoles were synthesized under one-pot conditions and isolated with good to excellent yields (50–96%) and purity (>98%). The observed results represent an example that proves that biomass-derived safer solvents can be introduced into a synthetically important transformation exhibiting higher chemical and environmental safety.

## Introduction

In the past few decades, transition-metal-catalyzed coupling and addition reactions have represented one of the most powerful and atom-economical strategies for efficiently assembling new carbon–carbon [1–3] and carbon–heteroatom [4–6] bonds. Thus, it has become the most attractive and facile methodology for creating various complex organic molecular structures from the laboratory to the industrial scale. Among these methods, the copper(I)-catalyzed azide–alkyne cycloaddition (CuAAC) reaction, the so-called click reaction [7], has received substantial attention for the selective synthesis of various 1,2,3-triazoles that are of utmost importance in the synthesis of biologically active compounds such as active pharmaceutical ingredients (APIs) [8–11] and pesticides [12], or metabolic labeling molecules in plant science [13], to name a few important applications. The CuAAC reactions can be easily carried out under mild reaction conditions and exhibit excellent functional group tolerance [7].

While water has been characterized as an ideal solvent for click reactions, the limited solubility of most organic substrates could limit its application. Thus, the transformations of either water-insoluble or solid compounds require a solvent to establish high reaction performance, i.e. homogeneous solutions with low viscosity. Accordingly, the CuAAC reactions are usually performed in fossil-based common organic reaction media having high vapor pressure, toxicity, flammability, etc., which could result in several serious environmental concerns. According to the FDA guideline [14], common polar aprotic organic solvents used for click reactions such as chlorinated hydrocarbons [15–16], toluene [16], tetrahydrofuran (THF) [17–18], *N*,*N*-dimethylformamide (DMF) [19–20], *N*-methylpyrrolidone (NMP) [21], dimethyl sulfoxide (DMSO) [17,19,22], or acetonitrile [23] are classified into Class 1 and 2, of which applications should be strictly limited, particularly in the pharmaceutical industry. To develop an environmentally benign alternative to this useful method, the identification of an alternative reaction medium is highly desired.

**Scheme 1:**
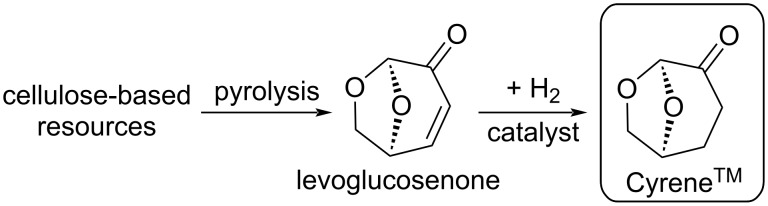
Synthesis of Cyrene^TM^ (dihydrolevoglucosenone) from cellulose-based feeds via levoglucosenone (LG).

Among the recently characterized biomass-based potential solvents dihydrolevoglucosenone (1*R*,5*S*)-7,8-dioxabicyclo-[3.2.1]octan-2-one, CAS: 53716-82-8) or Cyrene^TM^ (Scheme 1) has received increasing interest over the last few years. It can be produced from cellulose-containing feedstocks, through pyrolysis and a selective hydrogenation of levoglucosenone (Scheme 1). Regarding the market position, the Circa Group announced the production of Cyrene^TM^ of 1 ton/year in 2020, signifying the large-scale production of this new biobased molecule [24]. Cyrene^TM^ is a non-toxic substance with an LD_50_,_oral_ > 2000 mg/kg (OECD No. 423, acute toxicity method). E(L)C_50_ > 100 mg/L (daphnia and algae), and it is negative in the Ames test [25]. Recently, we determined key physicochemical properties of Cyrene^TM^ and showed that it has a negligible vapor pressure (<9.6 kPa) and low viscosity (<6.8 mPa·s) at typical transition-metal-catalyzed reaction temperatures (30–140 °C) [26].

Cyrene^TM^ has been successfully introduced into homogeneous [26–29] and heterogeneous [30–31] carbon–carbon and carbon–heteroatom bond-forming protocols. Although its reactive carbonyl group could limit its application when a strong base (aldol condensation [29]) or amines (potential Schiff-base formation) are present, a wide range of organic reactions, e.g., urea and amide formation [32–33], amide coupling [34], aldol condensation [35], C–H difluoromethylation [36], aromatic substitution [37], and MOFs synthesis [38] were demonstrated in Cyrene^TM^. Very recently, Fasano and Citarella first reported a CuAAC reaction in Cyrene^TM^ using 10 mol % Cu load, sodium ascorbate as base, and 24 h reaction time [39]. The protocol was successfully applied to synthesize more than 20 1,2,3-triazoles with excellent yields and purity.

Because the CuAAC reaction is a well-studied transformation, preparing various 1,2,3-triazoles in a less toxic and recyclable medium could further control and reduce the environmental impacts of this synthetically very important transformation.

Herein, we report a study on the copper(I)-catalyzed azide–alkyne cycloaddition (CuAAC) reaction in Cyrene^TM^ under mild conditions.

## Results and Discussion

**Scheme 2:**
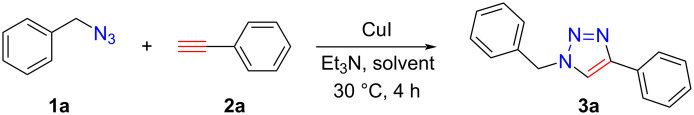
Copper-catalyzed azide–alkyne cycloaddition of benzyl azide (**1a**) and phenylacetylene (**2a**) in various solvents.

We recently demonstrated that Pd-catalyzed Heck reactions could be performed in Cyrene^TM^ [26]. To extend its applicability, we first compared the typical conventional organic media, selected biomass-derived solvents (i.e., levulinic acid and γ-valerolactone-based solvents), and Cyrene^TM^ in the transformation of 1.15 mmol benzyl azide (**1a**) and 1 mmol phenylacetylene (**2a**) in the presence of 0.01 mmol CuI and 0.1 mmol Et_3_N as a model reaction (Scheme 2) under typically used “click conditions” [7].

**Figure 1:**
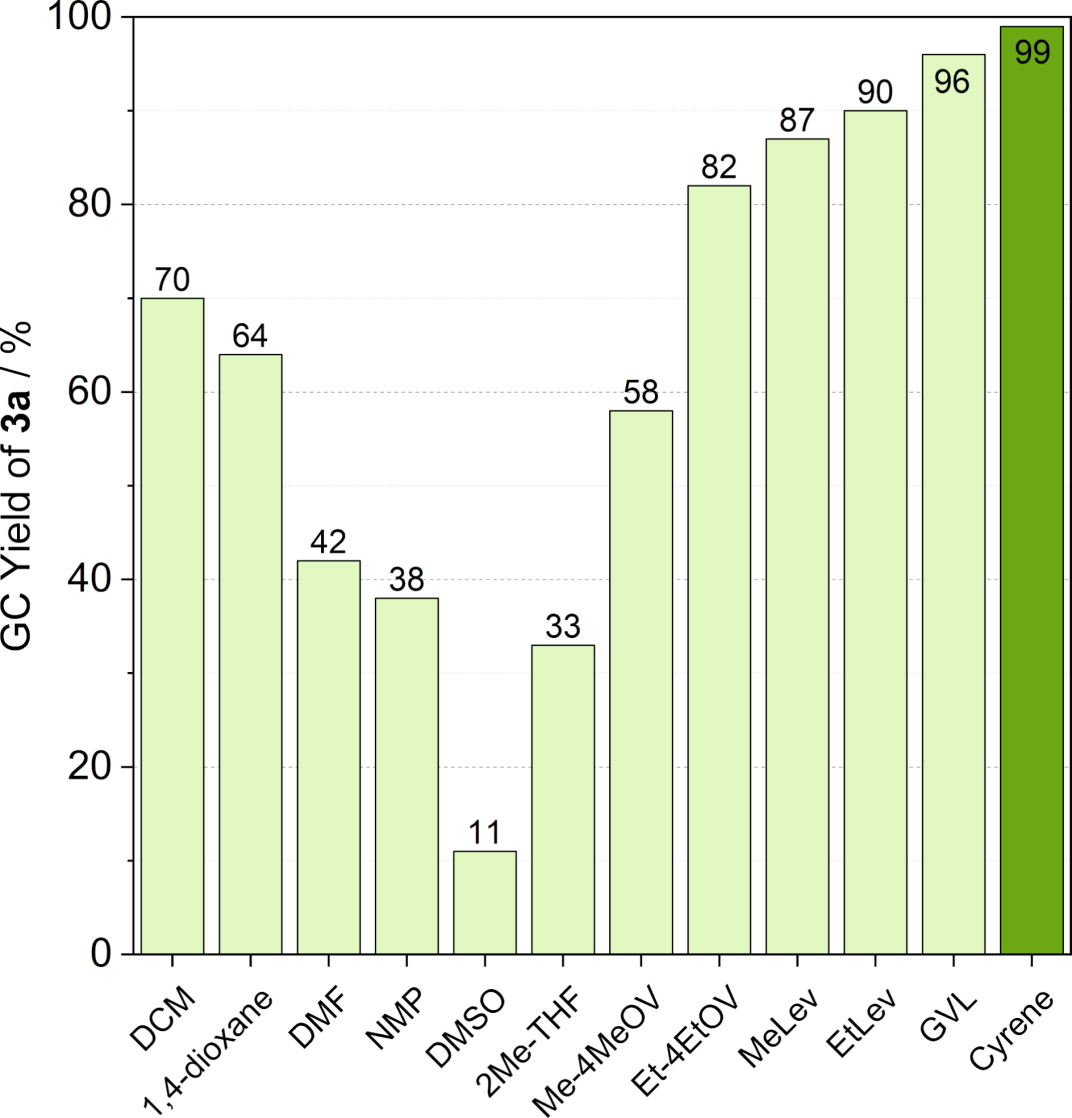
Comparison of various solvents used in the CuAAC reaction. Reaction conditions: 1.15 mmol benzyl azide, 1 mmol phenylacetylene, 2.5 mL solvent, 0.01 mmol CuI, 0.1 mmol Et_3_N, *T* = 30 °C, *t* = 4 h. DCM: dichloromethane, DMF: *N*,*N*-dimethylformamide, NMP: *N*-methylpyrrolidone, DMSO: dimethyl sulfoxide, 2Me-THF: 2-methyltetrahydrofuran, Me-4MeOV: methyl 4-methoxyvalerate, Et-4EtOV: ethyl 4-ethoxyvalerate, MeLev: methyl levulinate, EtLev: ethyl levulinate, GVL: γ-valerolactone.

In common organic solvents, the yields of 1-benzyl-4-phenyl-1*H*-1,2,3-triazole (**3a**) were moderate (DCM, 1,4-dioxane) or low (DMF, NMP, DMSO) (Figure 1). While low conversion was still detected in biomass-originated 2-MeTHF, MeLev, and EtLev established better performance. When their corresponding 4-alkoxy derivatives were applied, moderate (Me-4MeOV) or slightly lower (Et-4EtOV) conversions could be observed. However, significantly higher efficiencies were detected in GVL and Cyrene^TM^, which clearly verify that both solvents are appropriate for click chemistry.

**Figure 2:**
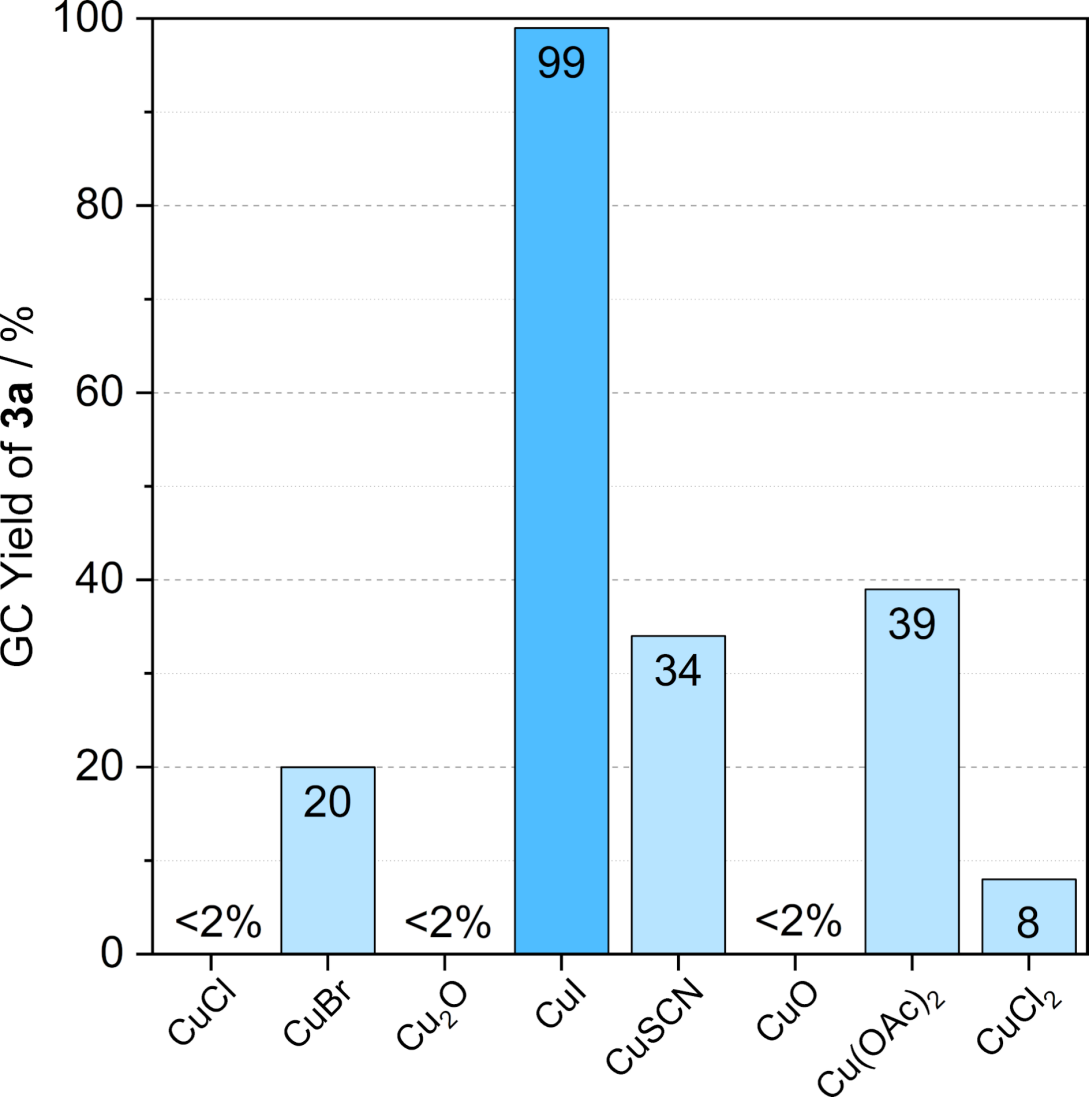
Effect of the Cu source used in the click reaction of benzyl azide (**1a**, 1.15 mmol) and phenylacetylene (**2a**, 1 mmol). Reaction conditions: 2.5 mL Cyrene^TM^, 1 mol % catalyst precursor, 0.1 mmol Et_3_N*, T* = 30 °C, *t* = 0.5 h.

The source of copper could also have a significant effect on the reaction’s performance [40]. Accordingly, both Cu(I) and Cu(II) salts (1 mol % Cu) were tested in the conversion of 1.15 mmol benzyl azide (**1a**) and 1 mmol phenylacetylene (**2a**) in 2.5 mL Cyrene^TM^ at 30 °C. All the selected Cu salts catalyzed the cycloaddition; however, Cu chlorides and oxides resulted in unexpectedly low product yields after 0.5 h. The solubility of Cu oxides was significantly lower in Cyrene^TM,^ indicated by a slightly turbid, inhomogeneous initial reaction mixture. Copper(I) bromide, thiocyanate, and acetate also gave low yields. However, CuI afforded almost complete conversion of **1a** under identical conditions (Figure 2). The results are in accordance with those obtained for Cu sources in different solvent systems [40–41].

Although CuAAC reactions can be efficiently performed in water, the moisture content of the organic reaction environment could have a significant effect on the efficiency of a transition-metal-catalyzed reaction. Because Cyrene^TM^ is fully miscible in water, investigating the possible effect of the water content on the reaction was highly desired. We found that a slight decrease in formation of product **3a** was detected when the moisture content was varied between 0.05 and 3.0 wt % (Table 1). A higher moisture content reduces the product formation; thus, keeping water content below 1% is necessary to maintain high reaction efficiency. The negative effect could be due to the decreased solubility of **2a** at higher water content [42–44].

**Table 1:** Effect of the water content on the CuAAC reaction of benzyl azide (**1a**) and phenylacetylene (**2a**).^a^

Entry	Water content/wt %	Yield **3a**^b^/%

1	<0.05	>99
2	1.0	88
3	2.0	86
4	3.0	70
5	4.0	47
6	5.0	29

^a^Reaction conditions: 1.15 mmol benzyl azide (**1a**), 1 mmol phenylacetylene (**2a**), 2.5 mL Cyrene^TM^, 1 mol % CuI, 0.1 mmol Et_3_N*, T* = 30 °C, *t* = 1 h. ^b^GC yield.

**Figure 3:**
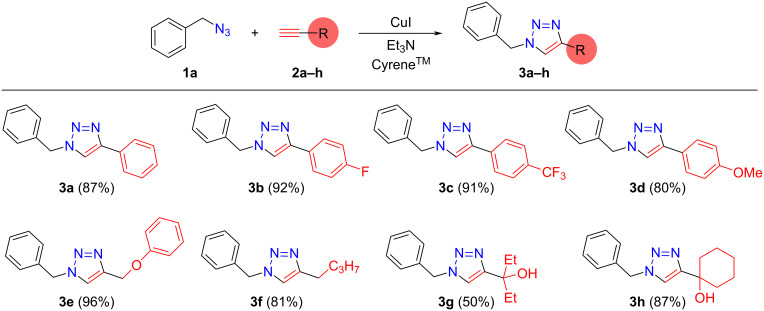
Copper-catalyzed azide–alkyne cycloaddition of benzyl azide (**1a**) and various acetylenes **2a–h** in Cyrene^TM^. Reaction conditions: 1.15 mmol **1a**, 1 mmol **2a–h**, 0.1 mmol Et_3_N, 0.01 mmol CuI, 2.5 mL Cyrene^TM^, *T* = 30 °C, *t* = 12 h; isolated yields based on **2a–h** are given in parentheses.

Hereafter, the readily available CuI was selected as a catalyst precursor to facilitate click reactions involving benzyl azide (**1a**) and various acetylenes **2b–h** in Cyrene^TM^ at 30 °C for 12 h (Figure 3). It should be noted that all components readily dissolved in Cyrene^TM^, resulting in clear, homogeneous reaction mixtures. With the exception of 3-(1-benzyl-1*H*-1,2,3-triazol-4-yl)pentan-3-ol (**3g**), the isolated 1,2,3-triazole derivatives were generally obtained with good to excellent yields (50–96%). Both electron-withdrawing (fluoro (**3b**) or trifluoromethyl (**3c**)) and electron-donating (methoxy, phenoxy, and alkyl (**3d–h**)) groups were tolerated on the acetylene reaction partner species. In accordance with results reported by Citarella et al. [39], excellent functional group tolerance was verified and the isolated yields are in the same range as reported by Citarella et al. (for **3a**, 90% [39] and 87% [39]). It should be noted that no Cu-catalyzed Glaser-coupling of acetylenes [45] was observed, indicating further the applicability of the present synthetic method.

**Figure 4:**
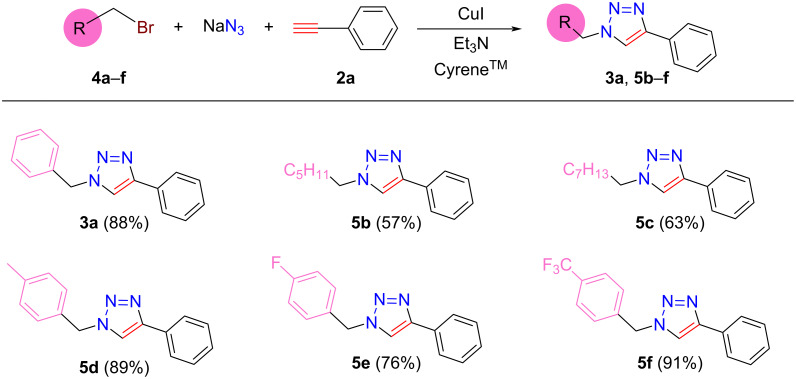
Consecutive synthesis of various *N*-substituted-4-phenyl-1*H*-1,2,3-triazoles in Cyrene^TM^. Reaction conditions: 1st step: 1.15 mmol **4a**–**e**, and 1.3 mmol NaN_3_, 2.5 mL Cyrene^TM^, *T* = 85 °C, *t* = 8 h. 2nd step: 1 mmol **2a**, 0.1 mmol Et_3_N, 0.01 mmol CuI, *T* = 30 °C, *t* = 12 h. Isolated yields based on phenylacetylene (**2a**) are given in parentheses.

Due to the excellent solvating power of Cyrene^TM^, the “one-pot” synthesis of 1,2,3-triazoles could be proposed to eliminate the preparation and isolation steps of azide components. This could open an even greener and facile protocol for CuAAC reactions. To demonstrate the one-pot synthesis of 1-benzyl-4-phenyl-1*H*-1,2,3-triazole (**3a**), 1.23 mmol benzyl bromide (**4a**), 1.57 mmol NaN_3_, 1.06 mmol phenylacetylene (**2a**), 0.011 mmol CuI, and 0.1 mmol Et_3_N were mixed in 2.5 mL of solvent and stirred at 85 °C. After 24 h, GC analysis verified a 90% yield of **3a**, which proves that the CuAAC reaction was completed in Cyrene^TM^ under one-pot conditions. After the work-up procedure, a satisfactory 84% yield of **3a** was obtained. When the one-pot reaction was sequenced, it first involved the synthesis of benzyl azide (**1a**) using 1.17 mmol benzyl bromide (**4a**) and 1.31 mmol NaN_3_ at 85 °C. After 8 h, 1.06 mmol phenylacetylene (**2a**), 0.01 mmol CuI, and 0.1 mmol Et_3_N were added to initiate the click reaction. The mixture was stirred at 30 °C for 12 h. The GC analysis showed complete conversion, and after the workup procedure, 88% **3a** was isolated with 98.5% purity. It is important to note that there are no differences between the isolated yields (cf. Figure 3 and Figure 4 for **3a**). According to the observation that the consecutive synthesis could be more efficient, the scope of the method was extended to synthesizing various *N*-substituted-4-phenyl-1*H*-1,2,3-triazoles in Cyrene^TM^ (Figure 4). It was shown that the protocol resulted in the formation of products **3a** and **5b–f** with yields of 57–91% depending on the structure of the bromide derivatives. The isolated yields were in the same range as reported by Citarella et al. [39].

**Figure 5:**
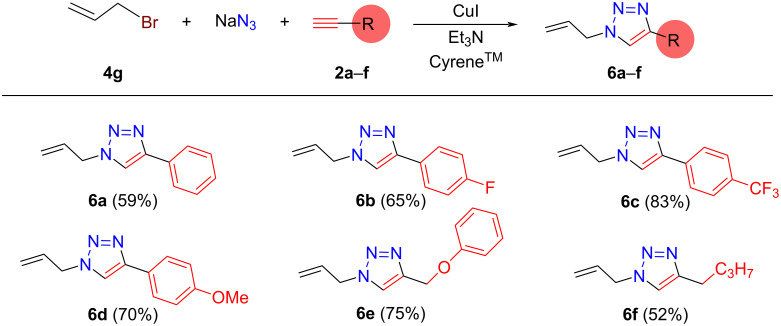
“One-pot” synthesis of various 1-allyl-4-substituted-1*H*-1,2,3-triazoles in Cyrene^TM^. Reaction conditions: 1st step: 1.15 mmol **4g**, 1.3 mmol NaN_3_, 2.5 mL Cyrene^TM^, *T* = 75 °C, *t* = 24 h. 2nd step: 1 mmol **2a**–**f**, 0.01, mmol CuI, 0.1 mmol Et_3_N, *T* = 30 °C, *t* = 12 h. Isolated yields based on corresponding acetylene derivatives are given in parentheses.

The presence of a terminal carbon–carbon double bond in a certain molecular structure could allow for efficient subsequent functionalization via, for example, an addition reaction, opening possibilities for building even more complex molecular skeletons involving 1,2,3-triazole units. Using allyl bromide in the reaction sequence results in the formation of 1-allyl-4-substituted-1*H*-1,2,3-triazoles bearing a terminal C–C double bond moiety. Thus, we attempted to prepare a series of 1-allyl-4-substituted-1*H*-1,2,3-triazoles (Figure 5, **6a**–**f**) from allyl bromide (**4g**) and selected acetylenes **2a**–**f**. Similar to the formation of *N*-substituted-4-phenyl-1*H*-1,2,3-triazoles, the method exhibits good functional group tolerance and gives the corresponding products **6a**–**f** with moderate and/or good isolated yields (52–83%).

**Figure 6:**
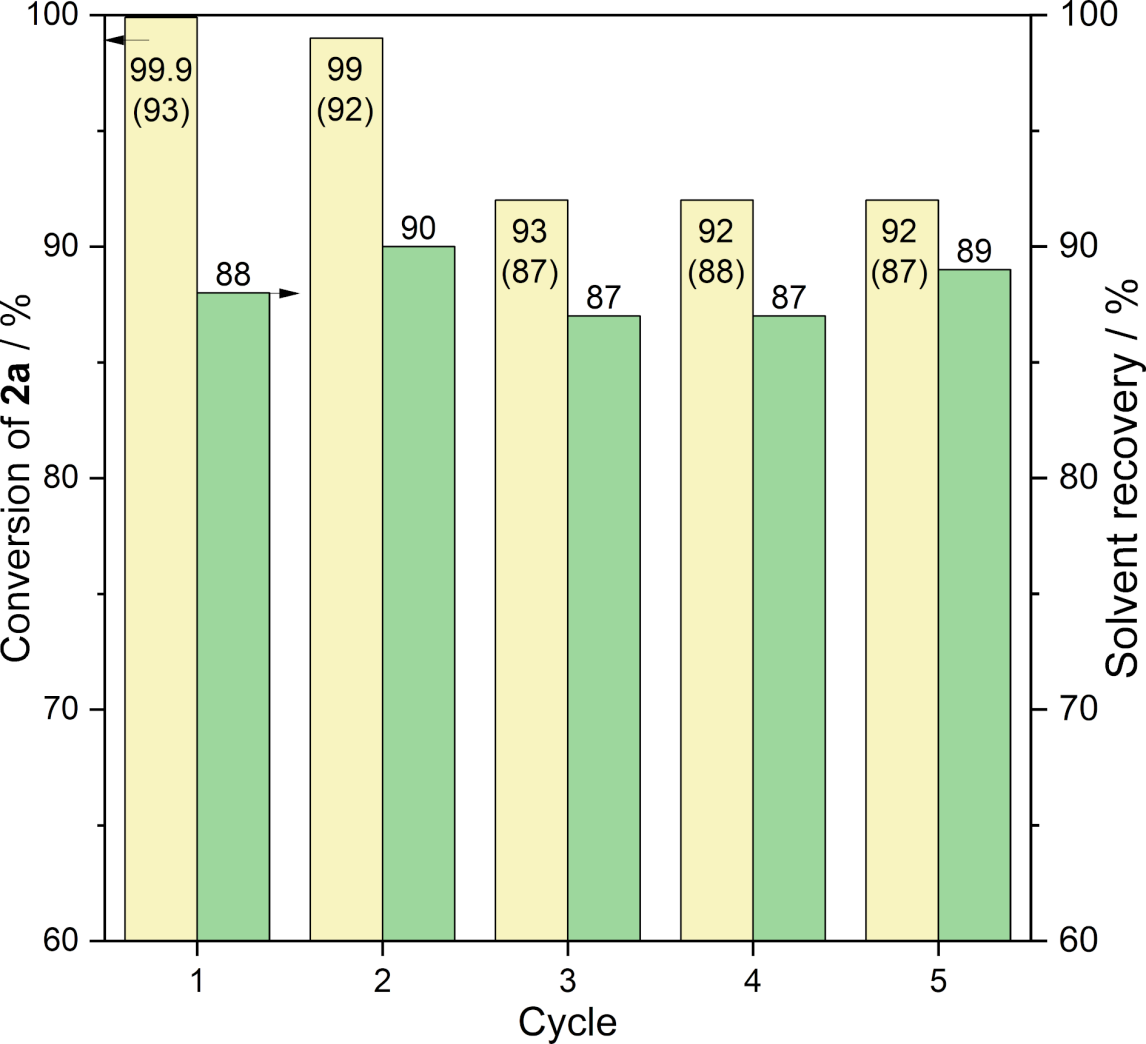
Solvent recovery for the CuAAC reaction of **1a** and **2a**. Reaction conditions: 12.5 mL Cyrene^TM^, 1 mol % CuI, 0.5 mmol Et_3_N*, T* = 30 °C, *t* = 2 h. Isolated yields are given in parentheses.

Our investigation finally focused on the solvent recovery and reuse, which is a key issue for large-scale applications. When 5 mmol **1a** and 5.75 mmol of **2a** were reacted in the presence of 1 mol % CuI and 0.5 mmol Et_3_N at 30 °C for 2 h, a >99.9% conversion was detected. After the reaction, 25 mL of cold water was added to precipitate **3a**, which was subsequently filtered, dried, and isolated with a yield of 93.7%. After the removal of volatile compounds from the aqueous phase by vacuum distillation, 88% of Cyrene^TM^ (13.7 g) was recovered. The reaction was repeated four times with the same procedure under identical conditions (same catalyst and substrate concentration). It was shown that Cyrene^TM^ could be successfully recovered for 4 consecutive runs, which resulted in high conversion of **1a** in each run (Figure 6).

To evaluate and compare the one-pot protocol with published methods, the environmental factor (E-factor) was calculated for the synthesis of **3a**. Considering an average 88% solvent recovery, the E-factor is 76. It is lower than that obtained in the conventional organic medium DMSO (E-factor = 104, [46]) and higher than the one calculated by Citarella (E-factor = 24, [39]) for the Cyrene^TM^-based protocol. However, they used a 15 times higher substrate loading.

## Conclusion

In conclusion, we have demonstrated that biomass-derived Cyrene^TM^ can be utilized as an alternative reaction medium for the one-pot copper(I)-catalyzed azide–alkyne cycloaddition (CuAAC) reaction of various acetylenes and azides. Due to the strong solvating power of Cyrene^TM^, a sequenced one-pot synthesis of triazoles was successfully demonstrated. The protocol was tested for a wide range of substrates, and successful synthesis and isolation of nineteen 1,2,3-triazole derivatives **3a**–**h**, **5b**–**f**, and **6a–f** with moderate to excellent isolated yields (50–96%) and purity (>98%) was shown.

## Experimental

The sources of chemicals are listed in Supporting Information File 1. ^1^H, ^13^C, and ^19^F NMR spectra were collected on a Bruker Avance 300 MHz or Bruker Avance-III 500 MHz instrument and processed by MestReNova v. 14.3.1-31739 (2022) MestreLab Research S. L.

GC analyses were performed on an HP 5890 N Series II instrument with Restek RTX^®^-50 capillary column (15 m × 0.25 mm × 0.25 µm) using H_2_ as a carrier gas. For the analysis, 10 μL of the reaction mixture was dissolved in 1 mL of ethyl acetate, followed by adding 10 μL toluene as the internal standard. Heating profile of GC–FID analysis: The initial temperature was 100 °C and was hold for 0.5 min. Heating rate: 40 °C/min up to the final temperature of 270 °C. The final temperature was held for 4.25 min.

The water content of Cyrene^TM^ was measured on a Methrom 684 KF Coulometer at Balint Analitika Ltd, Budapest, Hungary.

Cyrene^TM^ was purchased from Sigma-Aldrich Kft. Budapest, Hungary. Its purification was performed by vacuum distillation (18–20 mmHg, 114–116 °C) and stored under argon before subsequent use. The purity (>99.99%) was checked by ^1^H and ^13^C NMR spectroscopy. ^1^H NMR (300 MHz, CDCl_3_, TMS) δ 5.10 (s, 1H, CH), 4.71 (s, 1H, CH), 4.05 (d, *J* = 7.3 Hz, 1H, CH), 3.96 (t, *J* = 6.3 Hz, 1H, CH), 2.73–2.02 (m, 4H, CH_2_); ^13^C NMR (75 MHz, CDCl_3_, TMS) δ 200.3 (CO), 101.5 (CH), 73.1 (CH), 67.6 (CH_2_), 31.1 (CH_2_), 29.9 (CH_2_). The NMR data correspond to the published results.

Methyl 4-methoxyvalerate and ethyl 4-ethoxyvalerate were prepared using the published method [47].

The synthesis of benzyl azide, detailed experimental procedures, and characterization of prepared compounds are reported in Supporting Information File 1.

### General procedure for click reaction in Cyrene^TM^

In a 4 mL screw-cap vial, 1.15 mmol of benzyl azide (**1a**), 1 mmol corresponding acetylene, 0.1 equiv Et_3_N, and 0.01 mmol CuI, were dissolved in 2.5 mL Cyrene^TM^. The reaction mixture was stirred overnight at a given temperature. After the reaction, 20 mL of cold distilled water was added, followed by intensive stirring. The solid product was filtered, washed with distilled water (3 × 5 mL), and dried until constant weight under the fume hood. The detailed experimental procedure, as well as the characterization of isolated compounds, are provided in Supporting Information File 1.

### General procedure for click reaction in Cyrene^TM^ under one-pot conditions

In a 4 mL screw-cap vial, 1.15 mmol of corresponding bromine and 1.3 mmol NaN_3_ were dissolved in 2.5 mL Cyrene^TM^ and stirred at a given temperature. After a given reaction time, 1 mmol of the corresponding acetylene compound, 0.1 mmol of Et_3_N, and 0.01 mmol of CuI were added to the reaction mixture and reacted for a given time at a given temperature. The work-up procedure is similar to the one given above. The detailed experimental procedure, as well as the characterization of isolated compounds, are provided in Supporting Information File 1.

## Supporting Information

File 1 Source of chemicals, detailed experimental procedure, and characterization of isolated compounds.

## Data Availability

All data that supports the findings of this study is available in the published article and/or the supporting information of this article.
