# Supplementary material for: Azide–alkyne cycloaddition (click) reaction in biomass-derived solvent CyreneTM under one-pot conditions
Source: Beilstein J Org Chem. 2025 Jul 30;21:1544–51. doi: 10.3762/bjoc.21.117 (PMC12320739; doi:10.3762/bjoc.21.117)
Supplement: File 1 — Source of chemicals, detailed experimental procedure, and characterization of isolated compounds. [file Beilstein_J_Org_Chem-21-1544-s001.pdf]

BEILSTEIN JOURNAL OF ORGANIC CHEMISTRY

Supporting Information

for

Azide–alkyne cycloaddition (click) reaction in biomass-derived solvent Cyrene^{TM} under one-pot conditions

Zoltán Medgyesi and László T. Mika

Beilstein J. Org. Chem. **2025,** *21,* 1544–1551. doi:10.3762/bjoc.21.117

Source of chemicals, detailed experimental procedure, and characterization of isolated compounds

License and Terms: This is a supporting information file under the terms of the Creative Commons Attribution License (https://creativecommons.org/licenses/by/4.0). Please note that the reuse, redistribution and reproduction in particular requires that the author(s) and source are credited and that individual graphics may be subject to special legal provisions.
The license is subject to the *Beilstein Journal of Organic Chemistry* terms and conditions: (https://www.beilstein-journals.org/bjoc/terms)

Table of contents

Source of chemicals S2

Preparation and characterization of the synthesized compounds S2

^{1}H and ^{13}C NMR spectra of Cyrene^{TM} S10

^{1}H, ^{13}C, and ^{19}F NMR data for synthesized compounds S11

References S34

Source of chemicals

Toluene, diethyl ether, and *N*-methyl-2-pyrrolidone were obtained from Molar Chemicals Ltd., Budapest, Hungary, and used without further purification.

1-Ethynyl-4-fluorobenzene, 1-ethynyl-4-(trifluoromethyl)benzene, 4-methylbenzyl bromide, 4-fluorobenzyl bromide, 4-(trifluoromethyl)benzyl bromide and copper(I) iodide were purchased from TCI Chemicals and used without further purification.

Benzyl bromide, allyl bromide, 1-hexyl bromide, 1-octyl bromide, sodium azide, 1,4-dioxane, dimethyl sulfoxide, 2-methyltetrahydrofuran, methyl levulinate, ethyl levulinate, γ-valerolactone, phenylacetylene, 1-ethynyl-4-methoxybenzene, phenyl propargyl ether, 1-hexyne, 3-ethyl-1-pentyn-3-ol, 1-ethynyl-1-cyclohexanol, copper(I) thiocyanate, copper(I) chloride, copper(I) bromide were purchased from Sigma-Aldrich Kft., Budapest, Hungary and used as received.

Preparation and characterization of the synthesized compounds

Benzyl azide (1a)

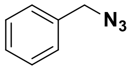

20.1 g (117.5 mmol) benzyl bromide, 15 g (230.7 mmol) sodium azide 152 µL (156.1 mg, 1.17 mmol) were reacted in 50 mL ethanol. The reaction was performed under reflux for 24 h. The reaction mixture was then dissolved in 35 mL diethyl ether and washed with 80 mL water. The organic phase was washed with brine (3 × 10 mL). The solution was dried with $MgSO_4$ and filtered, and the solvent was removed in vacuo. Yield: 13.9 g (88%) yellowish oil. ^{1}H NMR (500 MHz, $CDCl_3$, TMS, ppm): δ= 4.32 (s, 2H), 7.29 – 7.40 (m, 5H).^{13}C NMR (126 MHz, $CDCl_3$, TMS, ppm): δ= 54.80, 128.22, 128.31, 128.84, 135.38. It corresponds to the published results.[1]

1-Benzyl-4-phenyl-1*H*-1,2,3-triazole (3a)

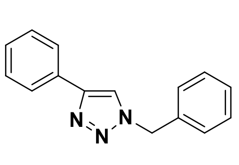

104 µL (104.3 mg, 1.02 mmol) phenylacetylene, 152 µL (156.1 mg, 1.17 mmol) benzyl azide, 10 µL (6.0 mg) triethylamine, 2.1 mg copper(I) iodide, and 2.5 mL of Cyrene^{TM} as solvent. The reaction mixture was stirred overnight at 30 °C. Then, 20 mL of cold distilled water is added, followed by intensive stirring. The solid product was filtered, washed with distilled water (3 × 5 mL). It was dried until constant weight under the fume hood. Yield: 209.9 mg (87%) white solid. ^{1}H NMR (500 MHz, $CDCl_3$, TMS, ppm): δ= 5.56 (s, 2H), 7.28 – 7.33 (m, 3H), 7.34 – 7.42 (m, 5H), 7.66 (s, 1H), 7.79 (dd, *J* = 8.4, 1.4 Hz, 2H). ^{13}C NMR (126 MHz, $CDCl_3$, TMS, ppm): δ= 54.21, 119.52, 125.69, 128.05, 128.16, 128.77, 128.80, 129.15, 130.54, 134.69, 148.21.MS: m/z (%):116.05 (100), 91.00 (82), 206.00 (45), 89.00 (30), 104.00 (23), 65.00 (22), 235.05 (13), 207.00 (12). 62.95 (10), 117.05 (9). It corresponds to the published results.[2]

1-Benzyl-4-(4-fluorophenyl)-1*H*-1,2,3-triazole (3b)

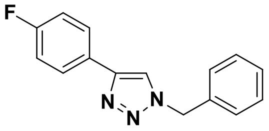

144.4 mg (1.20 mmol) 1-ethynyl-4-fluorobenzene, 184.7 mg, (1.38 mmol) benzyl azide, 10 µL (9.1 mg) triethylamine, 2.1 mg copper(I) iodide, and 2.5 mL of Cyrene^{TM} as solvent. The reaction mixture was stirred overnight at 30 °C. Then, 20 mL of cold distilled water is added, followed by intensive stirring. The solid product was filtered and washed with distilled water (3 × 5 mL). It was dried until constant weight under the fume hood. Yield: 280.1 mg (92%) white solid.^{1}H NMR (500 MHz, $CDCl_3$, TMS, ppm): δ= 5.55 (s, 2H), 7.05 – 7.10 (m, 2H), 7.30 (dd, *J* = 7.6, 2.0 Hz, 2H), 7.34 – 7.40 (m, 3H), 7.62 (s, 1H), 7.73 – 7.78 (m, 2H).^{13}C NMR (126 MHz, $CDCl_3$, TMS, ppm): δ= 54.25, 115.78 (d, *J* = 21.4 Hz), 119.27, 126.80 (d, *J* = 2.5 Hz), 127.43 (d, *J* = 7.6 Hz), 128.08, 128.83, 129.18, 134.62, 147.36, 161.67, 163.63. ^{19}F NMR (282 MHz, $CDCl_3$, TMS, ppm): δ= –113.55. MS: m/z (%): 134.00 (100), 91.00(93), 224.00 (62), 107.00 (38), 65.00 (27), 104.00 (18), 253.05 (17), 198.00 (13), 225.00 (11), 197.00 (9). It corresponds to the published results.[2]

1-Benzyl-4-(4-(trifluoromethyl)phenyl)-1*H*-1,2,3-triazole (3c)

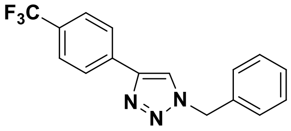

169.5 mg, (0.99 mmol) 1-ethynyl-4-(trifluoromethyl)benzene, 152.9 mg, (1.15 mmol) benzyl azide, 10 µL (8.1 mg) triethylamine, 2.1 mg copper(I) iodide, and 2.5 mL of Cyrene^{TM} as solvent. The reaction mixture was stirred overnight at 30 °C. Then, 20 mL of cold distilled water is added, followed by intensive stirring. The solid product was filtered and washed with distilled water (3 × 5 mL). It was dried until constant weight under the fume hood. Yield: 276.0 mg (91 %) white solid. ^{1}H NMR (500 MHz, $CDCl_3$, TMS, ppm): δ= 5.59 (s, 2H), 7.30 – 7.35 (m, 2H), 7.36 – 7.43 (m, 3H), 7.64 (d, *J* = 8.4 Hz, 2H), 7.75 (s, 1H), 7.91 (d, *J* = 8.2 Hz, 2H). ^{13}C NMR (126 MHz, $CDCl_3$, TMS, ppm) δ= 54.39, 120.29, 124.10 (q, *J* = 271.3 Hz), 125.18, 125.81 (q, *J* = 4.2 Hz), 128.15, 128.97, 129.26, 130.00 (q, *J* = 32.3 Hz), 134.00, 134.41, 146.86. ^{19}F NMR (282 MHz, $CDCl_3$, TMS, ppm): δ= -62.59. δ= -113.55. MS: m/z (%): 91.00 (100), 184.00 (64), 274.05 (42), 65.00 (23), 104.05 (20), 179.05 (14), 303.05 (10), 275.05 (10), 206.05 (8), 157.00 (8). It corresponds to the published results.[3]

1-Benzyl-4-(4-methoxyphenyl)-1*H*-1,2,3-triazole (3d)

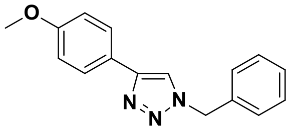

138.4 mg, (1.04 mmol) 1-ethynyl-4-methoxybenzene, 157.9 mg, (1.19 mmol) benzyl azide, 10 µL (8.1 mg) triethylamine, 2.2 mg copper(I) iodide, and 2.5 mL of Cyrene^{TM} as solvent. The reaction mixture was stirred overnight at 30 °C. Then, 20 mL of cold distilled water is added, followed by intensive stirring. The solid product was filtered and washed with distilled water (3 × 5 mL). It was dried until constant weight under the fume hood. Yield: 223.6 mg (80%) white solid.^{1}H NMR (500 MHz, $CDCl_3$, TMS, ppm): δ= 3.82 (s, 3H), 5.54 (s, 2H), 6.90 – 6.94 (m, 2H), 7.29 (dd, *J* = 7.6, 2.0 Hz, 2H), 7.33 – 7.41 (m, 3H), 7.57 (s, 1H), 7.69 – 7.74 (m, 2H). ^{13}C NMR (126 MHz, $CDCl_3$, TMS, ppm): δ=54.17, 55.30, 114.20, 118.70, 123.30, 127.00, 128.03, 128.72, 129.12, 134.80, 148.09, 159.60. MS: m/z (%): 206.95 (100), 236.05 (98), 91.00 (97), 146.00 (86), 119.00 (50), 65.00 (41), 265.05 (38), 209.00 (30), 280.95 (28), 237.00 (22). It corresponds to the published results.[3]

1-Benzyl-4-(phenoxymethyl)-1*H*-1,2,3-triazole (3e)

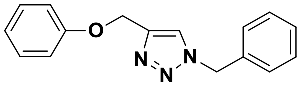

136.9 mg, (1.04 mmol) phenyl propargyl ether, 157.9 mg, (1.19 mmol) benzyl azide, 10 µL (8.7 mg) triethylamine, 2.4 mg copper(I) iodide, and 2.5 mL of Cyrene™ as solvent. The reaction mixture was stirred overnight at 30 °C. Then, 20 mL of cold distilled water is added, followed by intensive stirring. The solid product was filtered and washed with distilled water (3 × 5 mL). It was dried until constant weight under the fume hood. Yield: 255.5 mg (96%) white solid. ^{1}H NMR (500 MHz, $CDCl_3$, TMS, ppm): δ= 5.18 (s, 2H), 5.52 (s, 2H), 6.94 – 6.99 (m, 3H), 7.27 (d, *J* = 8.5 Hz, 4H), 7.33 – 7.40 (m, 3H), 7.53 (s, 1H).^{13}C NMR (126 MHz, $CDCl_3$, TMS, ppm): δ= 54.24, 62.04, 114.75, 121.24, 122.59, 128.11, 128.81, 129.14, 129.52, 134.46, 144.72, 158.18. MS: m/z (%):91.00 (100), 144.05 (31), 65.00 (15), 172.05 (11), 94.00 (9), 92.00 (8), 104.00 (7), 39.00 (7), 265.05 (6), 117.05 (6).It corresponds to the published results.[4]

1-Benzyl-4-butyl-1*H*-1,2,3-triazole (3f)

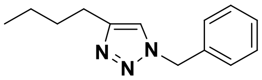

82.5 mg, (1.00 mmol) 1-hexyne, 151.4 mg, (1.14 mmol) benzyl azide, 10 µL (6.8 mg) triethylamine, 1.9 mg copper(I) iodide, and 2.5 mL of Cyrene™ as solvent. The reaction mixture was stirred overnight at 30 °C. Then, 20 mL of cold distilled water is added, followed by intensive stirring. The solid product was filtered and washed with distilled water (3 × 5 mL). It was dried until constant weight under the fume hood. Yield: 175.7 mg (81%) white solid. ^{1}H NMR (500 MHz, $CDCl_3$, TMS, ppm): δ= 0.91 (t, *J* = 7.3 Hz, 3H), 1.36 (h, *J* = 7.4 Hz, 2H), 1.62 (p, *J* = 7.5 Hz, 2H), 2.69 (t, *J* = 7.7 Hz, 2H), 5.49 (s, 2H), 7.19 (s, 1H), 7.25 (dd, *J* = 7.7, 1.9 Hz, 2H), 7.32 – 7.39 (m, 3H). ^{13}C NMR (126 MHz, $CDCl_3$, TMS, ppm): δ= 13.82, 22.32, 25.42, 31.52, 53.96, 120.49, 127.95, 128.58, 129.04, 135.02, 148.98. MS: m/z (%): 91.00 (100), 65.00 (10), 92.00 (9), 41.00 (8), 173.05 (5), 104.00 (5), 39.00 (4), 96.05 (3), 69.05 (3), 144.05 (3), 215.10 (0.3). It corresponds to the published results.[5]

3-(1-Benzyl-1*H*-1,2,3-triazol-4-yl)pentan-3-ol (3g)

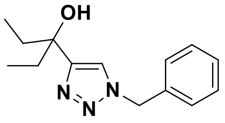

115.0 mg, (1.02 mmol) 3-ethyl-1-pentyn-3-ol, 153.1 mg, (1.15 mmol) benzyl azide, 10 µL (7.8 mg) triethylamine, 2.3 mg copper(I) iodide, and 2.5 mL of Cyrene™ as solvent. The reaction mixture was stirred overnight at 30 °C. Then, 20 mL of cold distilled water and 10 mL diethyl ether are added, followed by intensive stirring. The ether phase was washed with water (3 × 3 mL). The solution was dried with $MgSO_4$ and filtered, and the solvent was removed in vacuo. Yield: 127.0 mg (50%), slightly yellowish solid. ^{1}H NMR (500 MHz, $CDCl_3$, TMS, ppm): δ= 0.80 (t, *J* = 7.5 Hz, 6H), 1.78 – 1.94 (m, 4H), 2.50 (s, 1H), 5.51 (s, 2H), 7.24 (dd, *J* = 7.6, 2.0 Hz, 2H), 7.30 – 7.41 (m, 4H). ^{13}C NMR (126 MHz, $CDCl_3$, TMS, ppm): δ= 7.80, 33.75, 54.09, 74.11, 120.44, 127.89, 128.66, 129.10, 134.83, 153.57. MS: m/z (%): 91.00 (100), 216.05 (28), 65.00 (9), 92.00 (8), 146.05 (7), 217,00 (4), 57.00 (3), 41.00 (2), 160.00 (2), 38.95 (2).

1-(1-Benzyl-1*H*-1,2,3-triazol-4-yl)cyclohexanol (3h)

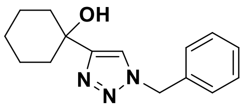

125.9 mg, (1.01 mmol) 1-ethynyl-1-cyclohexanol, 155.4 mg, (1.15 mmol) benzyl azide, 10 µL (8.8 mg) triethylamine, 2.3 mg copper(I) iodide, and 2.5 mL of Cyrene™ as solvent. The reaction mixture was stirred overnight at 30 °C. Then, 20 mL of cold distilled water and 10 mL diethyl ether are added, followed by intensive stirring. The ether phase was washed with water (3 × 3 mL). The solution was dried with $MgSO_4$, filtered, and the solvent was removed in vacuo. Yield: 225.8 mg (87%) white solid. ^{1}H NMR (500 MHz, $CDCl_3$, TMS, ppm): δ= 1.28 – 1.39 (m, 1H), 1.53 (dq, *J* = 9.0, 4.4 Hz, 2H), 1.61 (dq, *J* = 9.0, 4.7 Hz, 1H), 1.67 – 1.78 (m, 2H), 1.86 (s, 1H), 1.90 – 1.98 (m, 2H), 2.47 (s, 1H), 5.49 (s, 2H), 7.27 (d, *J* = 2.6 Hz, 2H), 7.37 (d, *J* = 7.3 Hz, 4H). ^{13}C NMR (126 MHz, $CDCl_3$, TMS, ppm): δ= 21.94, 25.35, 38.11, 54.14, 69.55, 119.55, 128.12, 128.71, 129.10, 134.67, 156.12. MS: m/z (%): 91.00 (100), 206.95 (17), 65.00 (14), 92.00 (13), 257.00 (11), 55.00 (10), 41.00 (8), 214.05 (8), 96.10 (8), 69,05 (7). It corresponds to the published results.[6]

1-Hexyl-4-phenyl-1*H*-1,2,3-triazole (5b)

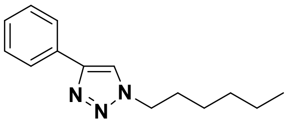

185.7 mg (1.12 mmol) 1-hexyl bromide, 92.6 mg (1.42 mmol) sodium azide and 2.5 mL of Cyrene™ as solvent. It was heated and stirred at 85 °C for 8 h. Then, 106.0 mg (1.04 mmol) phenylacetylene, 10 µL (12.5 mg) triethylamine, 2.0 mg copper(I) iodide were added. The reaction mixture was stirred overnight at 30 °C. Then, 20 mL of cold distilled water is added, followed by intensive stirring. The solid product was filtered and washed with distilled water (3 × 5 mL). It dried until constant weight under the fume hood. Yield: 134.6 mg (57%) beige solid. ^{1}H NMR (500 MHz, $CDCl_3$, TMS, ppm): δ= 0.85 – 0.91 (m, 3H), 1.26 – 1.40 (m, 6H), 1.93 (p, *J* = 7.2 Hz, 2H), 4.38 (t, *J* = 7.2 Hz, 2H), 7.29 – 7.36 (m, 1H), 7.42 (t, *J* = 7.6 Hz, 2H), 7.74 (s, 1H), 7.83 (dd, *J* = 8.3, 1.3 Hz, 2H).^{13}C NMR (126 MHz, $CDCl_3$, TMS, ppm): δ= 13.95, 22.42, 26.17, 30.32, 31.17, 50.43, 119.41, 125.69, 128.06, 128.82, 130.77, 147.71. MS: m/z (%): 117.05 (100), 43.00 (53), 104.05 (26), 41.00 (25), 116.05 (24), 229.10 (18), 89.00 (18), 103.00 (16), 200.10 (16), 90.00 (15). It corresponds to the published results.[6]

1-Octyl-4-phenyl-1*H*-1,2,3-triazole (5c)

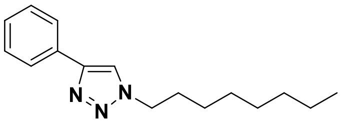

220.5 mg (1.14 mmol) 1-octyl bromide, 92.8 mg (1.43 mmol) sodium azide and 2.5 mL of Cyrene™ as solvent. It was heated and stirred at 85 °C for 8 h. Then, 107.7 mg (1.05 mmol) phenylacetylene, 10 µL (11.4 mg) triethylamine, 2.1 mg copper(I) iodide were added. The reaction mixture was stirred overnight at 30 °C. Then, 20 mL of cold distilled water is added, followed by intensive stirring. The solid product was filtered and washed with distilled water (3 × 5 mL). It dried until constant weight under the fume hood. Yield: 170.9 mg (63%) beige solid. ^{1}H NMR (500 MHz, $CDCl_3$, TMS, ppm): δ= 0.84 – 0.91 (m, 3H), 1.21 – 1.40 (m, 10H), 1.94 (p, *J* = 7.5 Hz, 2H), 4.38 (t, *J* = 7.2 Hz, 2H), 7.29 – 7.36 (m, 1H), 7.39 – 7.45 (m, 2H), 7.74 (s, 1H), 7.83 (dd, *J* = 8.3, 1.3 Hz, 2H). ^{13}C NMR (126 MHz, $CDCl_3$, TMS, ppm): δ= 14.06, 22.60, 26.51, 28.98, 29.05, 30.36, 31.71, 50.44, 119.40, 125.69, 128.06, 128.82, 130.77, 147.72. MS: m/z (%): 117.05 (100), 43.00 (44), 41.00 (34), 104.00 (33), 116.05 (21), 145.05 (20), 57.00 (18), 257.10 (16), 89.00 (16), 118.05 (15). It corresponds to the published results.[2]

1-(4-Methylbenzyl)-4-phenyl-1*H*-1,2,3-triazole (5d)

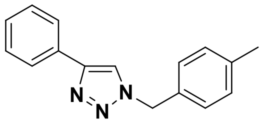

209.4 mg (1.13 mmol) 4-methylbenzyl bromide, 109.2 mg (1.68 mmol) sodium azide and 2.5 mL of Cyrene™ as solvent. It was heated and stirred at 85 °C for 8 h. Then, 108.7 mg (1.06 mmol) phenylacetylene, 10 µL (12.5 mg) triethylamine, 2.3 mg copper(I) iodide were added. The reaction mixture was stirred overnight at 30 °C. Then, 20 mL of cold distilled water is added, followed by intensive stirring. The solid product was filtered and washed with distilled water (3 × 5 mL). It dried until constant weight under the fume hood. Yield: 235.6 mg (89%) white solid. ^{1}H NMR (500 MHz, $CDCl_3$, TMS, ppm): δ= 2.35 (s, 3H), 5.51 (s, 2H), 7.16 – 7.22 (m, 4H), 7.27 – 7.32 (m, 1H), 7.35 – 7.41 (m, 2H), 7.63 (s, 1H), 7.78 (dd, *J* = 8.4, 1.4 Hz, 2H). ^{13}C NMR (126 MHz, $CDCl_3$, TMS, ppm): δ= 21.16, 54.01, 119.40, 125.68, 128.09, 128.12, 128.77, 129.79, 130.61, 131.66, 138.71, 148.13. MS: m/z (%): 116.05 (100), 105.05 (77), 220.05 (51), 89.00 (26), 77.00 (23), 117.05 (21), 118.05 (20), 79.00 (18), 249.10 (16), 103.00 15). It corresponds to the published results.[7]

1-(4-Fluorobenzyl)-4-phenyl-1*H*-1,2,3-triazole (5e)

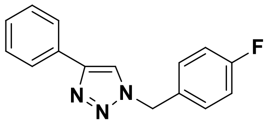

223.0 mg (1.18 mmol) 4-fluorobenzyl bromide, 100.6 mg (1.55 mmol) sodium azide and 2.5 mL of Cyrene™ as solvent. It was heated and stirred at 85 °C for 8 h. Then, 109.9 mg (1.08 mmol) phenylacetylene, 10 µL (14.4 mg) triethylamine, 2.6 mg copper(I) iodide were added. The reaction mixture was stirred overnight at 30 °C. Then, 20 mL of cold distilled water is added, followed by intensive stirring. The solid product was filtered and washed with distilled water (3 × 5 mL). It was dried until constant weight under the fume hood. Yield: 209.0 mg (76%) white solid. ^{1}H NMR (500 MHz, $CDCl_3$, TMS, ppm): δ= 5.53 (s, 2H), 7.03 – 7.06 (m, 2H), 7.26 – 7.34 (m, 3H), 7.40 (dd, *J* = 10.5, 4.7 Hz, 2H), 7.66 (s, 1H), 7.79 (dd, *J* = 5.1, 3.3 Hz, 2H). ^{13}C NMR (126 MHz, $CDCl_3$, TMS, ppm): δ= 53.45, 116.15 (d, *J* = 21.4 Hz), 119.41, 125.70, 128.25, 128.84, 129.93 (d, *J* = 7.6 Hz), 130.45, 130.58 (d, *J* = 2.5 Hz), 148.33, 163.87 (d, *J* = 2.5 Hz). ^{19}F NMR (282 MHz, $CDCl_3$, TMS, ppm) δ= -112.66. MS: m/z (%):116.05 (100), 109.00 (67), 224.00 (31), 89.00 (25), 83.00 (17), 122.00 (12), 253.05 (10), 62.95 (9), 117.05 (9), 225.00 (6). It corresponds to the published results.[8]

4-Phenyl-1-(4-(trifluoromethyl)benzyl)-1*H*-1,2,3-triazole (5f)

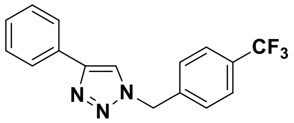

279.2 mg (1.17 mmol) 4-(trifluoromethyl)benzyl bromide, 100.8 mg (1.55 mmol) sodium azide and 2.5 mL of Cyrene™ as solvent. It was heated and stirred at 85°C for 8 h. Then, 111.3 mg (1.09 mmol) phenylacetylene, 10 µL (11.3 mg) triethylamine, 2.5 mg copper(I) iodide was added. The reaction mixture was stirred overnight at 30 °C. Then, 20 mL of cold distilled water is added, followed by intensive stirring. The solid product was filtered and washed with distilled water (3 × 5 mL). It was dried until constant weight under the fume hood. Yield: 299.9 mg (91%) white solid. ^{1}H NMR (500 MHz, $CDCl_3$, TMS, ppm): δ= 5.64 (s, 2H), 7.30 – 7.36 (m, 1H), 7.37 – 7.45 (m, 4H), 7.64 (d, *J* = 8.1 Hz, 2H), 7.71 (s, 1H), 7.81 (dd, *J* = 5.1, 3.3 Hz, 2H). ^{13}C NMR (126 MHz, $CDCl_3$, TMS, ppm): δ= 53.54, 119.61, 123,80 (q, *J* = 273.0 Hz), 125.75, 126.16 (q, *J* = 3.8 Hz), 128.19, 128.39, 128.89, 130.29, 131.08 (q, *J* = 33.2

Hz), 138.70, 148.58. ^{19}F NMR (282 MHz, $CDCl_3$, TMS, ppm) δ= -62.76. MS: m/z (%): 116.05 (100), 159.00 (22), 89.00 (19), 109.00 (16), 274.00 (15), 117.05 (9), 303.05 (6), 63.00 (6), 119.00 (4), 39.00 (3). It corresponds to the published results.[9]

1-Allyl-4-phenyl-1*H*-1,2,3-triazole (6a)

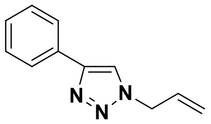

132.8 mg (1.10 mmol) allyl bromide, 106.6 mg (1.63 mmol) sodium azide and 2.5 mL of Cyrene™ as solvent. It was heated and stirred at 75 °C for 24 h. Then, 106.1 mg (1.03 mmol) phenylacetylene, 10 µL (10.5 mg) triethylamine, 2.2 mg copper(I) iodide was added. The reaction mixture was stirred overnight at 30 °C. Then, 20 mL of cold distilled water is added, followed by intensive stirring. The solid product was filtered and washed with distilled water (3 × 5 mL). It was dried until constant weight under the fume hood. Yield: 112.7 mg (59%) yellow solid. ^{1}H NMR (500 MHz, $CDCl_3$, TMS, ppm): δ= 5.01 (d, *J* = 6.1 Hz, 2H), 5.30 – 5.40 (m, 2H), 6.05 (ddt, *J* = 16.3, 10.2, 6.1 Hz, 1H), 7.29 – 7.36 (m, 1H), 7.41 (d, *J* = 15.3 Hz, 2H), 7.76 (s, 1H), 7.80 – 7.85 (m, 2H). ^{13}C NMR (126 MHz, $CDCl_3$, TMS, ppm): δ= 52.76, 119.45, 120.18, 125.71, 128.15, 128.83, 130.62, 131.33, 148.02. MS: m/z (%): 116.00 (100), 89.00 (27), 41.00 (12), 39.00 (11), 156.00 (11), 117.00 (10), 185.00 (10), 62.95 (9), 54.00 (7), 50.95 (6). It corresponds to the published results.[10]

1-Allyl-4-(4-fluorophenyl)-1*H*-1,2,3-triazole (6b)

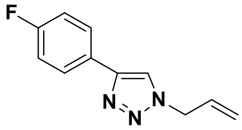

134.9 mg (1.11 mmol) allyl bromide, 107.2 mg (1.64 mmol) sodium azide and 2.5 mL of Cyrene™ as solvent. It was heated and stirred at 75 °C for 24 h. Then, 127.0 mg (1.05 mmol) 1-ethynyl-4-fluorobenzene, 10 µL (9.4 mg) triethylamine, 2.0 mg copper(I) iodide was added. The reaction mixture was stirred overnight at 30 °C. Then, 20 mL of cold distilled water is added, followed by intensive stirring. The solid product was filtered and washed with distilled water (3 × 5 mL). It was dried until constant weight under the fume hood. Yield: 140.3 mg (65 %) yellow solid. ^{1}H NMR (500 MHz, $CDCl_3$, TMS, ppm): δ= 5.01 (dt, *J* = 6.1, 1.4 Hz, 2H), 5.32 – 5.41 (m, 2H), 6.06 (ddt, *J* = 16.3, 10.2, 6.2 Hz, 1H), 7.07 – 7.14 (m, 2H), 7.73 (s, 1H), 7.77 – 7.83 (m, 2H). ^{13}C NMR (126 MHz, $CDCl_3$, TMS, ppm): δ= 52.80, 115.81 (d, *J* = 22.7 Hz), 119.22, 120.30, 126.8 (d, *J* = 3.8 Hz), 127.45 (d, *J* = 7.6 Hz), 131.26, 147.17, 161.69, 163.65. ^{19}F NMR (282 MHz, $CDCl_3$, TMS, ppm): δ= –113.62. MS: m/z (%):134.00 (100), 107.00 (38), 174.00 (20), 41.00 (16), 203.00 (14), 39.00 (12), 135.00 (10), 56.95 (9), 108.00 (8), 120.00 (7).

1-Allyl-4-(4-(trifluoromethyl)phenyl)-1*H*-1,2,3-triazole (6c)

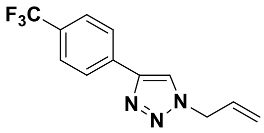

141.6 mg (1.17 mmol) allyl bromide, 100.7 mg (1.54 mmol) sodium azide and 2.5 mL of Cyrene™ as solvent. It was heated and stirred at 75 °C for 24 h. Then, 184.8 mg (1.09 mmol) 1-ethynyl-4-(trifluoromethyl)benzene, 10 µL (9.4 mg) triethylamine, 2.0 mg copper(I) iodide was added. The reaction mixture was stirred overnight at 30 °C. Then, 20 mL of cold distilled water is added, followed by intensive stirring. The solid product was filtered and washed with distilled water (3 × 5 mL). It was dried until constant weight under the fume

hood. Yield: 227.1 mg (83%) yellow solid. ^{1}H NMR (500 MHz, $CDCl_3$, TMS, ppm): δ= 5.05 (dt, *J* = 6.1, 1.4 Hz, 2H), 5.35 – 5.44 (m, 2H), 6.08 (ddt, *J* = 16.5, 10.2, 6.3 Hz, 1H), 7.67 (d, *J* = 8.4 Hz, 2H), 7.85 (s, 1H), 7.95 (d, *J* = 8.2 Hz, 2H). ^{13}C NMR (126 MHz, $CDCl_3$, TMS, ppm): δ= 52.92, 120.22, 120.59, 124.13 (q, *J* = 272 Hz), 125.86 (q, *J* = 3.8 Hz), 130.01 (q, *J* = 32.8 Hz),131.08, 134.07, 146.68. ^{19}F NMR (282 MHz, $CDCl_3$, TMS, ppm): δ= -62.60. MS: m/z (%):183.95 (100), 41.00 (56), 54.00 (19), 39.00 (17), 224.00 (16), 253.00 (15), 157.00 (12), 134.00 (12), 184.95 (11), 137.00 (9).

1-Allyl-4-(4-methoxyphenyl)-1*H*-1,2,3-triazole (6d)

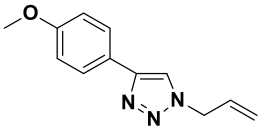

134.2 mg (1.11 mmol) allyl bromide 108.8 mg (1.67 mmol) sodium azide and 2.5 mL of Cyrene™ as solvent. It was heated and stirred at 75 °C for 24 h. Then, 132.4 mg (1.00 mmol) 1-ethynyl-4-methoxybenzene, 10 µL (8.4 mg), triethylamine, 2.3 mg copper(I) iodide was added. The reaction mixture was stirred overnight at 30 °C. Then, 20 mL of cold distilled water is added, followed by intensive stirring. The solid product was filtered and washed with distilled water (3 × 5 mL). It was dried until constant weight under the fume hood. Yield: 151.0 mg (70%) yellow solid. ^{1}H NMR (500 MHz, $CDCl_3$, TMS, ppm): δ= 3.83 (s, 3H), 5.00 (dt, *J* = 6.1, 1.5 Hz, 2H), 5.39 – 5.29 (m, 2H), 6.05 (ddt, *J* = 16.3, 10.2, 6.1 Hz, 1H), 6.92 – 6.98 (m, 2H), 7.67 (s, 1H), 7.73 – 7.77 (m, 2H). ^{13}C NMR (126 MHz, $CDCl_3$, TMS, ppm): δ= 52.73, 55.32, 114.24, 118.65, 120.09, 123.36, 127.02, 131.42, 147.89, 159.61. MS: m/z (%): 186.00 (100), 146.00 (85), 215.00 (57), 119.00 (47), 172.00 (45), 91.00 (30), 89.00 (30), 76.00 (29), 117.05 (28), 41.00 (24). It corresponds to the published results.[11]

1-Allyl-4-(phenoxymethyl)-1*H*-1,2,3-triazole (6e)

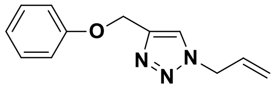

133.4 mg (1.10 mmol) allyl bromide, 104.4 mg (1.61 mmol) sodium azide and 2.5 mL of Cyrene™ as solvent. It was heated and stirred at 75 °C for 24 h. Then, 135.1 mg (1.02 mmol) phenyl propargyl ether, 10 µL (8.4 mg) triethylamine, 2.5 mg copper(I) iodide was added. The reaction mixture was stirred overnight at 30 °C. Then, 20 mL of cold distilled water and 10 mL diethyl ether are added, followed by intensive stirring. The ether phase was washed with water (3 × 3 mL). The solution was dried with $MgSO_4$, then filtered, and the solvent was removed in vacuo. Yield: 153.6 mg (75%) yellow oil. ^{1}H NMR (500 MHz, $CDCl_3$, TMS, ppm): δ= 4.97 (dt, J = 6.1, 1.4 Hz, 2H), 5.21 (s, 2H), 5.28 – 5.37 (m, 2H), 6.02 (ddt, J = 16.5, 10.2, 6.2 Hz, 1H), 6.94 – 7.01 (m, 3H), 7.26 – 7.32 (m, 2H), 7.61 (s, 1H).^{13}C NMR (126 MHz, $CDCl_3$, TMS, ppm): δ= 52.79, 62.04, 114.77, 120.34, 121.25, 122.46, 129.48, 131.12, 144.54, 158.22. MS: m/z (%): 94.00 (100), 41.00 (81), 39.00 (29), 122.05 (25), 67.00 (24), 65.00 (20), 215.00 (18), 54.00 (17), 77.00 (12), 66.00 (9).It corresponds to the published results.[12]

1-Allyl-4-butyl-1*H*-1,2,3-triazole (6f)

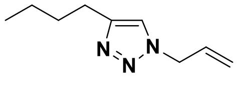

141.1 mg (1.16 mmol) allyl bromide, 101.6 mg (1.56 mmol) sodium azide and 2.5 mL of Cyrene™ as solvent. It was heated and stirred at 75 °C for 24 h. Then, 115.0 mg (1.07 mmol) 1-hexyne, 10 µL (8.4 mg) triethylamine, 2.4 mg copper(I) iodide added. The reaction mixture was stirred overnight at 30 °C. Then, 20 mL of cold distilled water and 10 mL diethyl ether are added, followed by

intensive stirring. The ether phase washed with water (3 × 3 mL). The solution was dried with $MgSO_4$, then filtered, and the solvent was removed in vacuo. Yield: 91.8 mg (52%) yellow oil. ^1H NMR (500 MHz, $CDCl_3$, TMS, ppm): δ= 0.93 (t, *J* = 7.3 Hz, 3H), 1.38 (h, *J* = 7.3 Hz, 2H), 1.65 (p, *J* = 7.6 Hz, 2H), 2.69 – 2.74 (m, 2H), 4.94 (dt, *J* = 6.1, 1.4 Hz, 2H), 5.25 – 5.35 (m, 2H), 6.01 (ddt, *J* = 16.3, 10.2, 6.2 Hz, 1H), 7.28 (s, 1H). ^{13}C NMR (126 MHz, $CDCl_3$, TMS, ppm): δ= 13.83, 22.31, 25.40, 31.59, 52.56, 119.75, 120.40, 131.63, 148.73. MS: m/z (%): 41.00 (100), 94.00 (28), 39.00 (17), 54.00 (14), 123.05 (14), 96.05 (11), 55.00 (7), 42.00 (7), 67.00 (7), 80.00 (6), 165.05 (0.6). It corresponds to the published results.[10]

^{1}H and ^{13}C NMR spectra of Cyrene

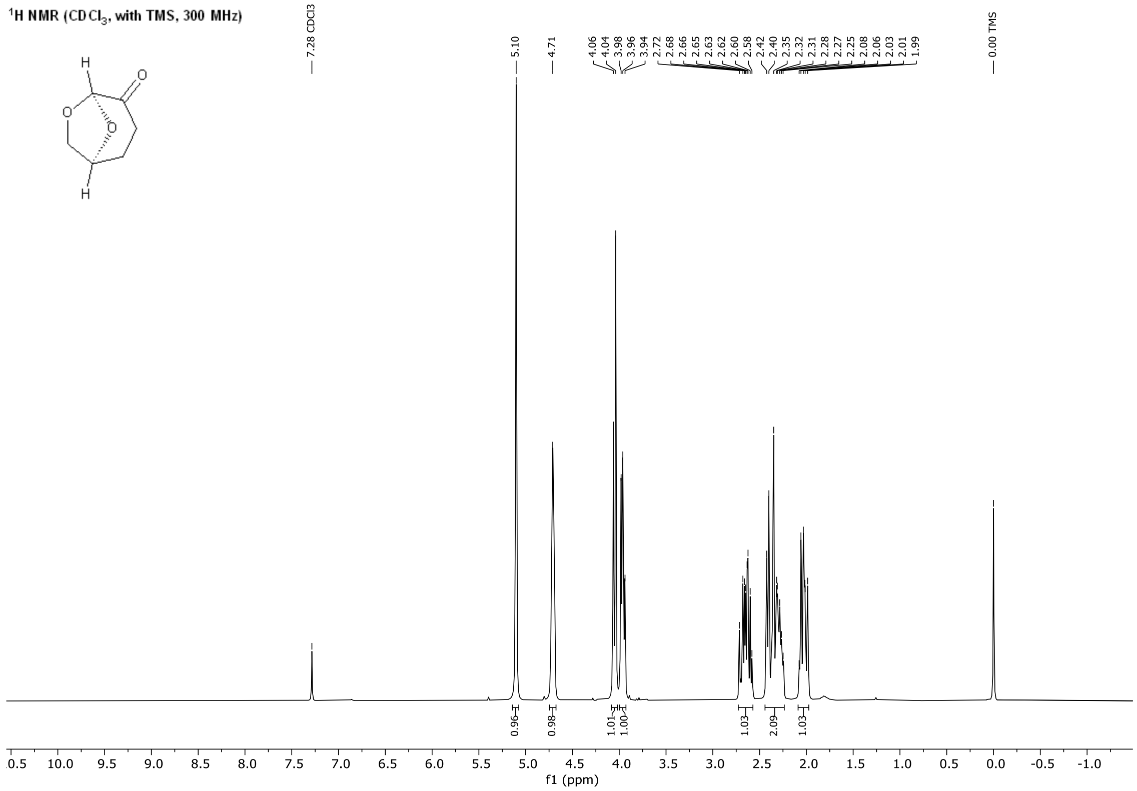

Figure S1. ^{1}H NMR spectrum of Cyrene

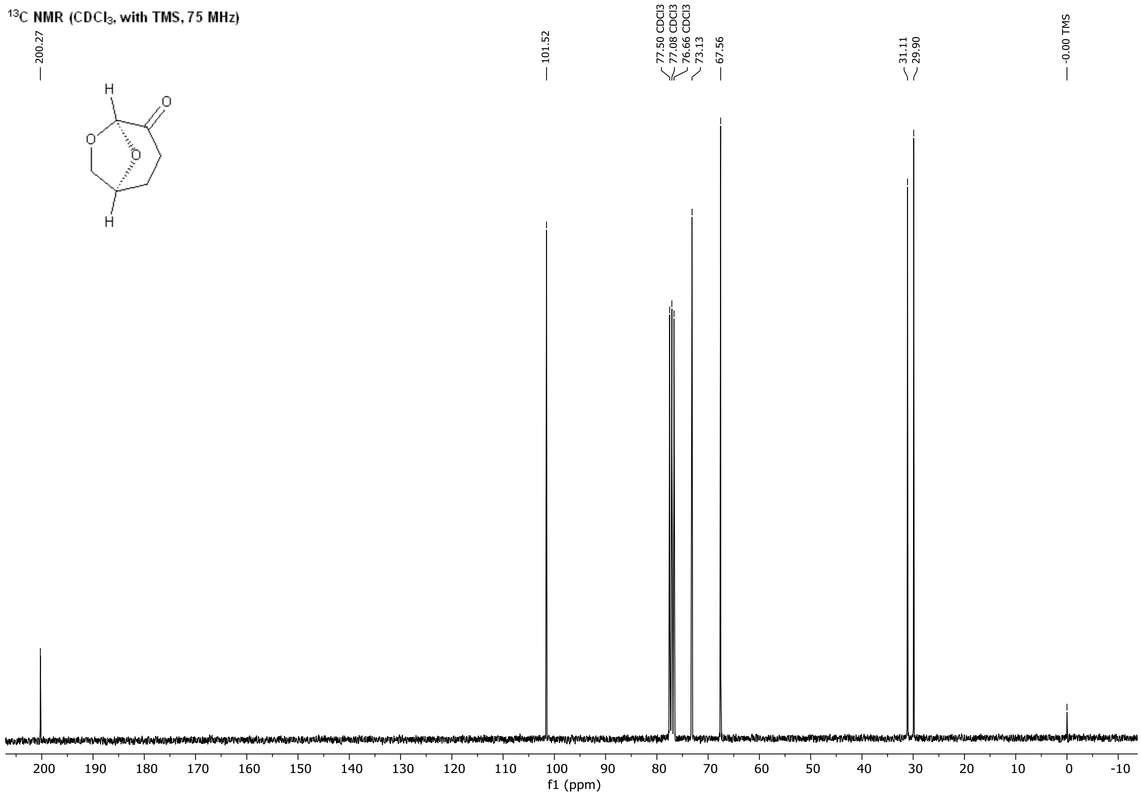

Figure S2. ^{13}C NMR spectrum of Cyrene

^1H, ^{13}C NMR and ^{19}F data for synthesized compounds

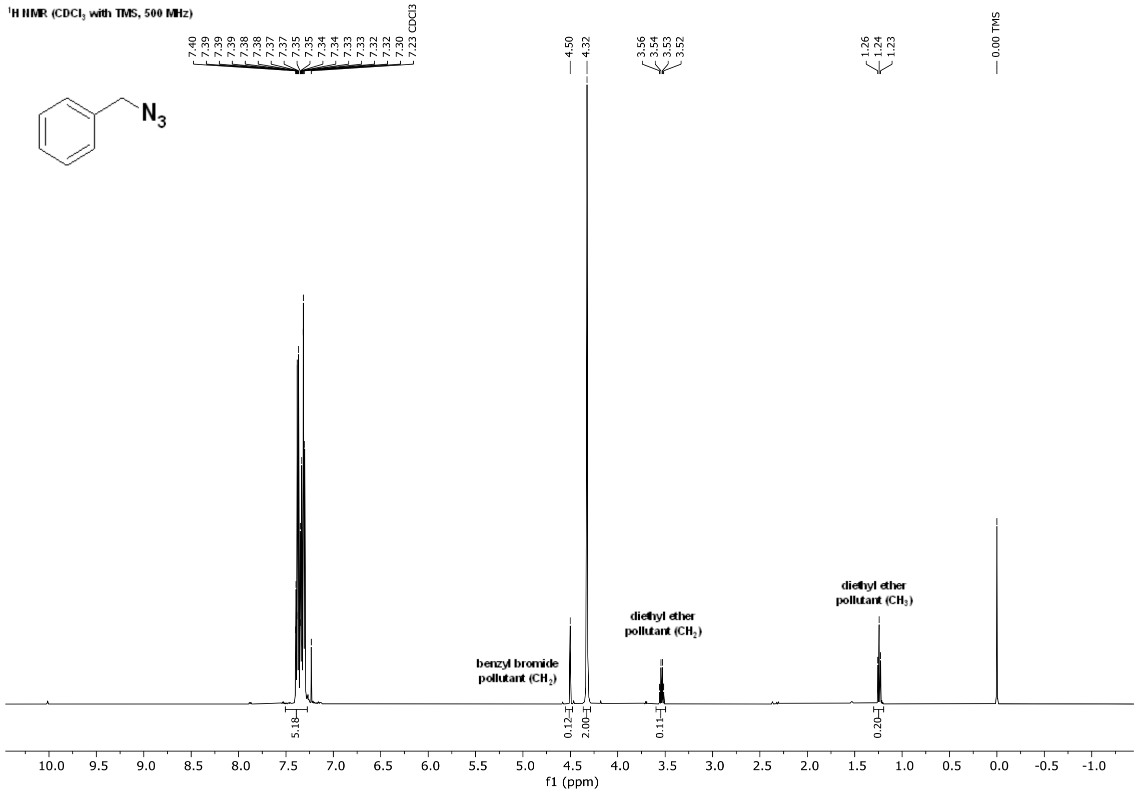

Figure S3. ^1H NMR spectrum of benzyl azide (**1a**)

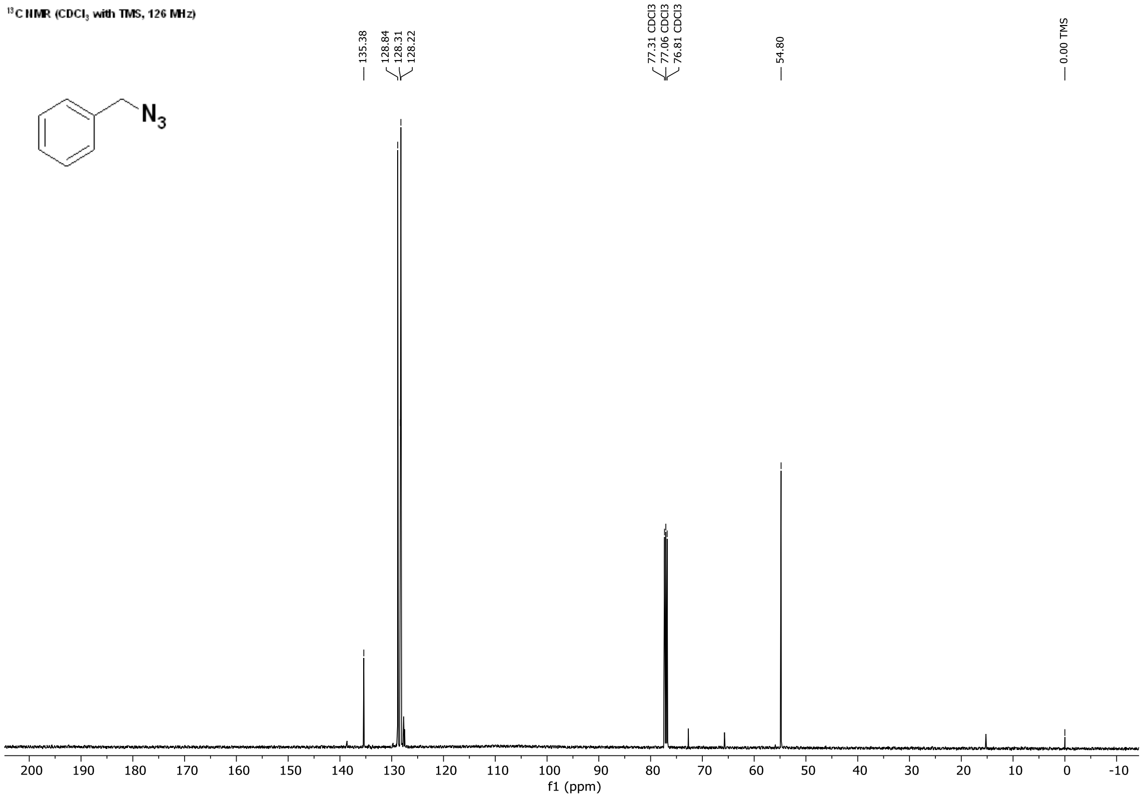

Figure S4. ^{13}C NMR spectrum of benzyl azide (**1a**)

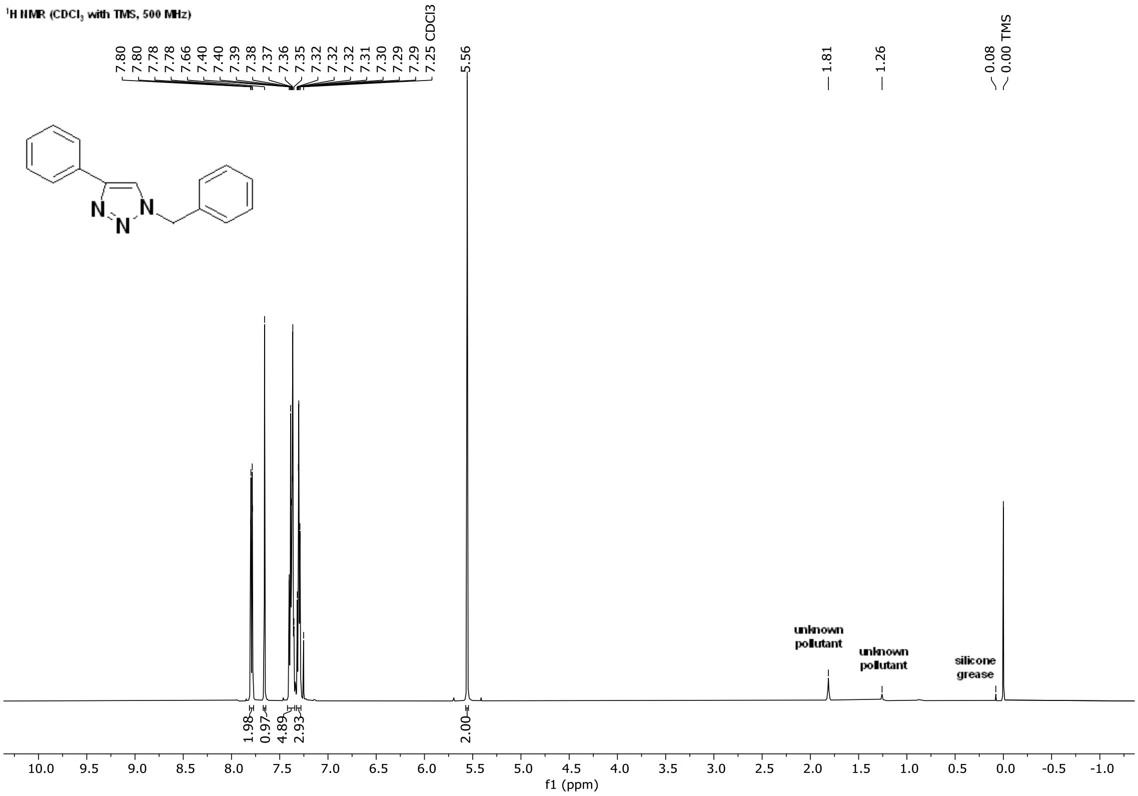

Figure S5. ^{1}H NMR spectrum of 1-benzyl-4-phenyl-1*H*-1,2,3-triazole (**3a**)

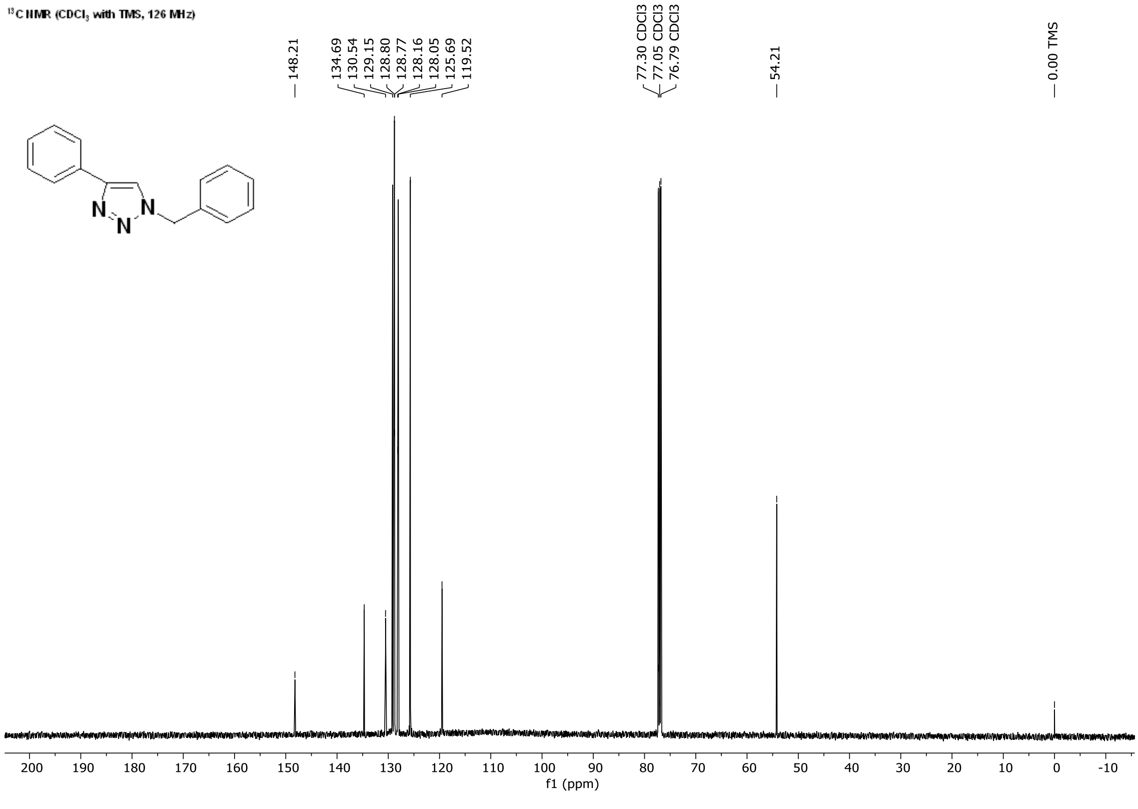

Figure S6. ^{13}C NMR spectrum of 1-benzyl-4-phenyl-1*H*-1,2,3-triazole (**3a**)

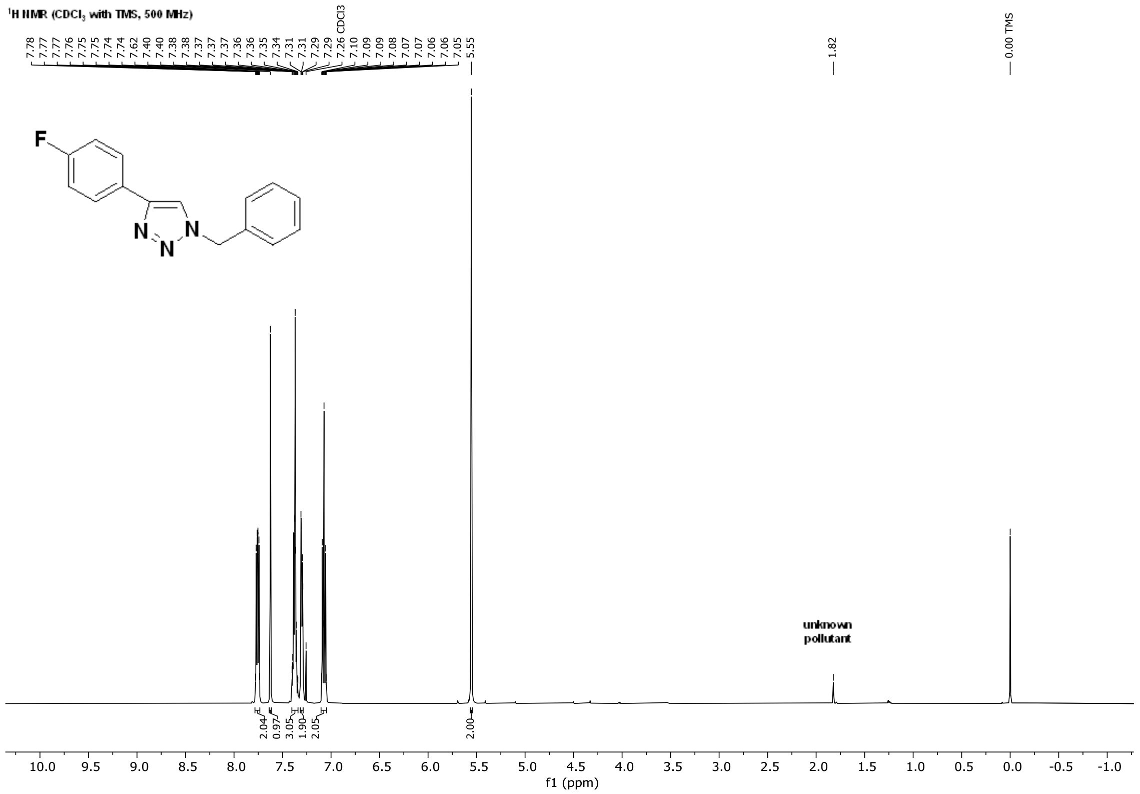

Figure S7. ^{1}H NMR spectrum of 1-benzyl-4-(4-fluorophenyl)-1*H*-1,2,3-triazole (**3b**)

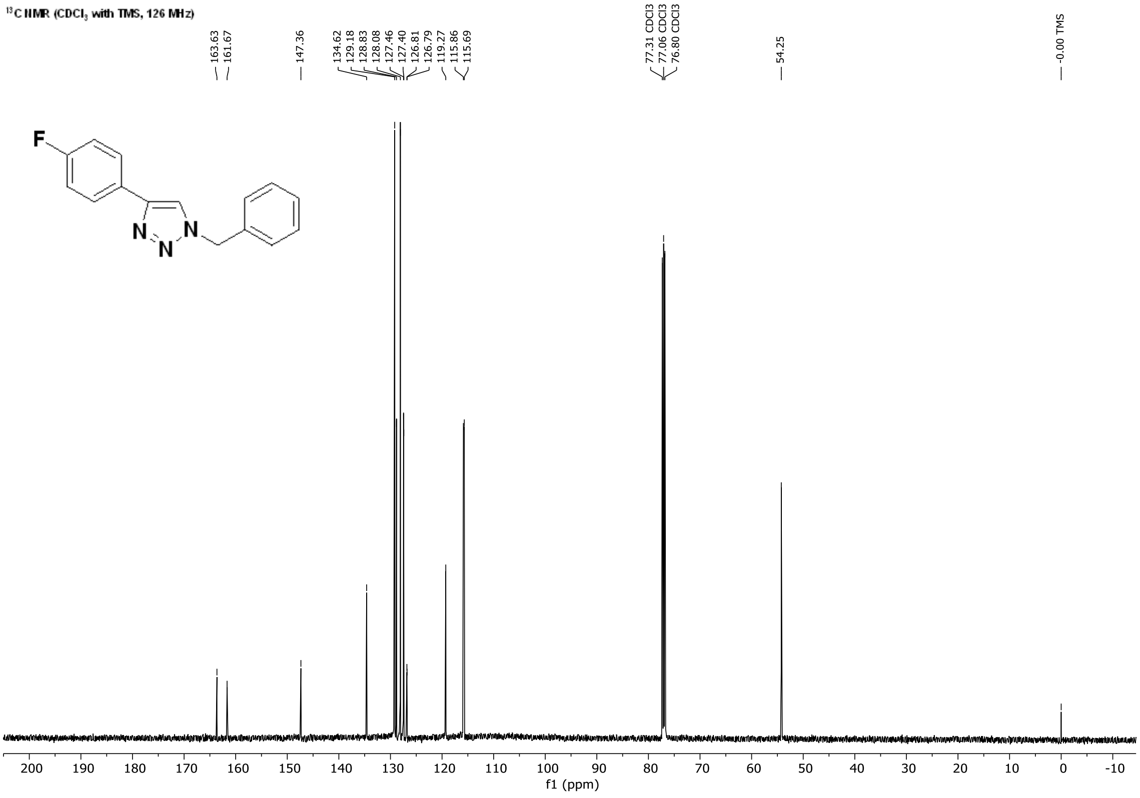

Figure S8. ^{13}C NMR spectrum of 1-benzyl-4-(4-fluorophenyl)-1*H*-1,2,3-triazole (**3b**)

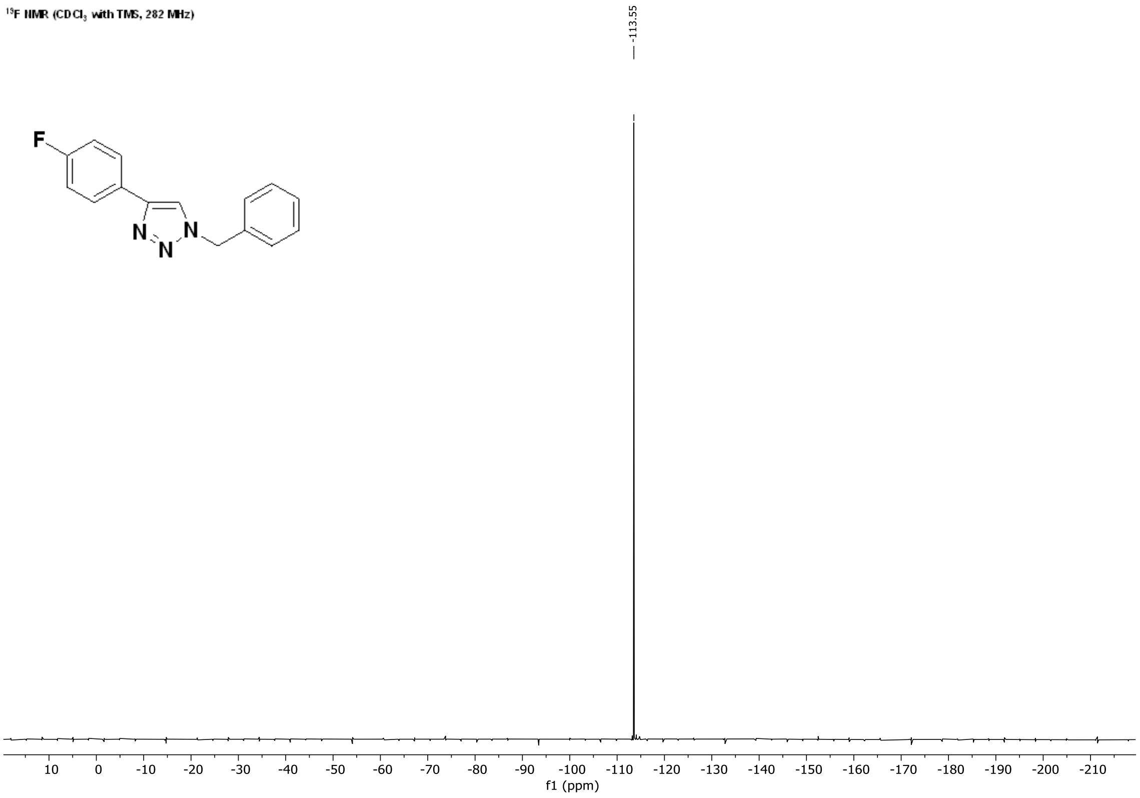

Figure S9. ^{19}F NMR spectrum of 1-benzyl-4-(4-fluorophenyl)-1*H*-1,2,3-triazole (**3b**)

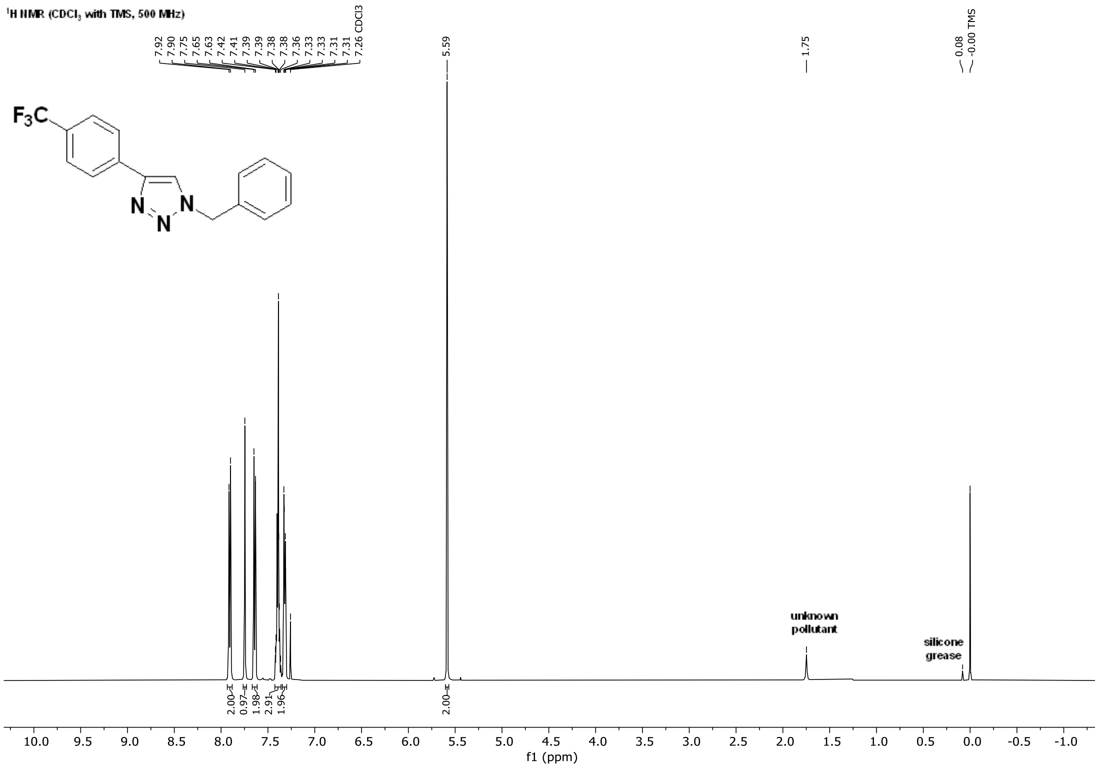

Figure S10. ^{1}H NMR spectrum of 1-benzyl-4-(4-(trifluoromethyl)phenyl)-1*H*-1,2,3-triazole (**3c**)

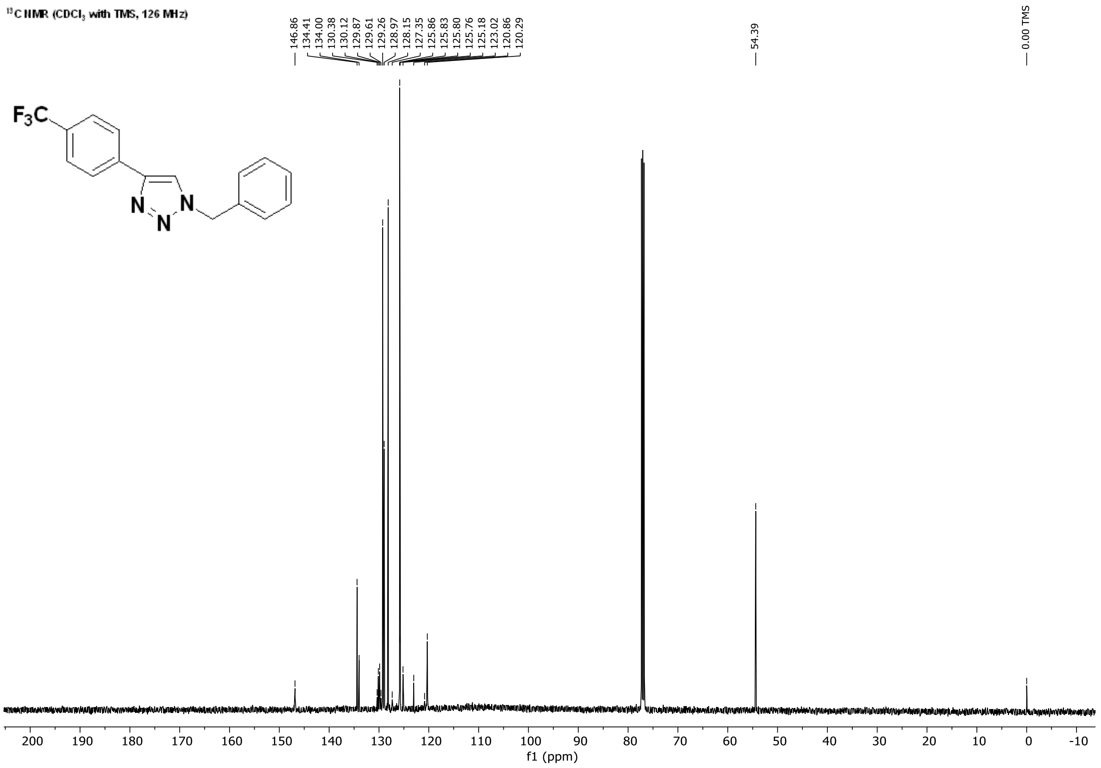

Figure S11. ^{13}C NMR spectrum of 1-benzyl-4-(4-(trifluoromethyl)phenyl)-1*H*-1,2,3-triazole (**3c**)

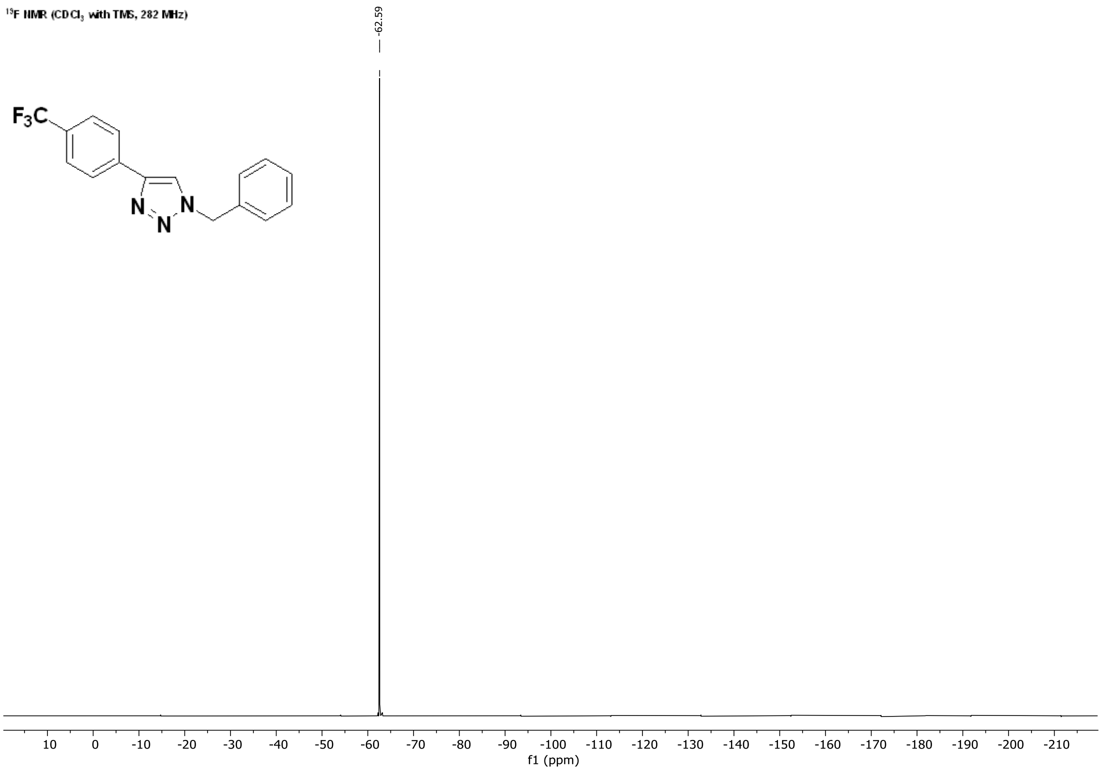

Figure S12. ^{19}F NMR spectrum of 1-benzyl-4-(4-(trifluoromethyl)phenyl)-1*H*-1,2,3-triazole (**3c)**

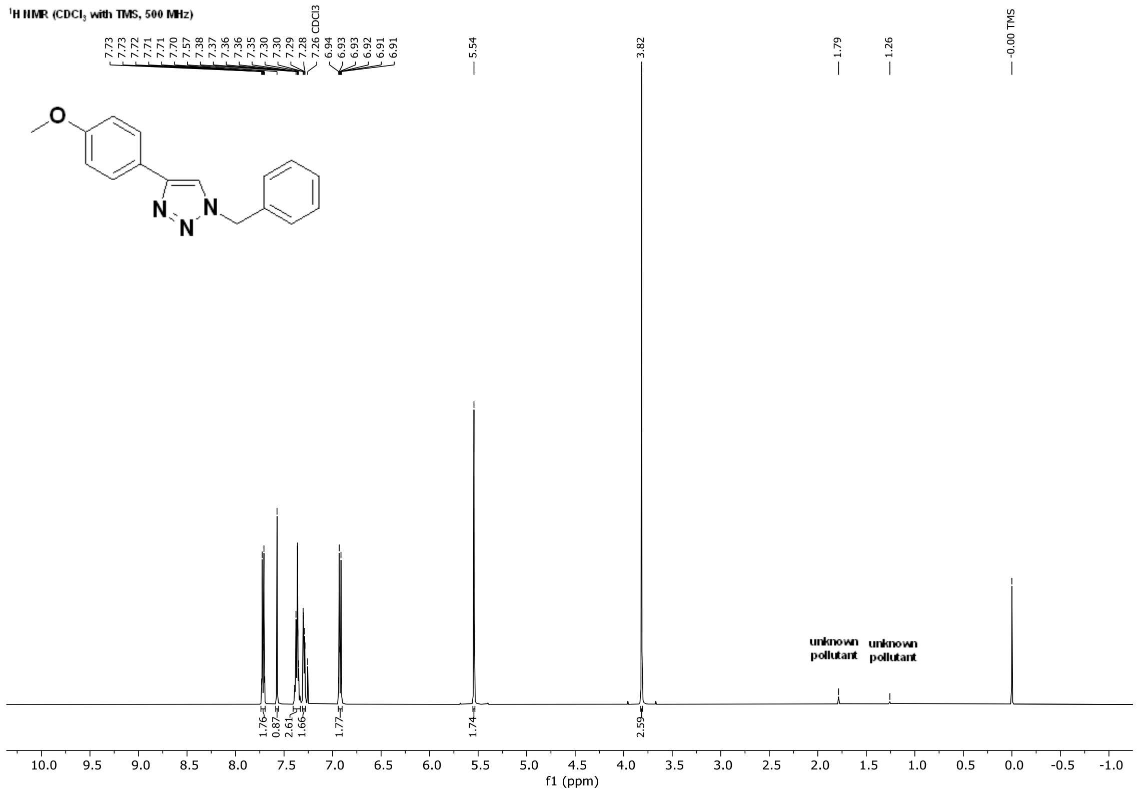

Figure S13. ^{1}H NMR spectrum of 1-benzyl-4-(4-methoxyphenyl)-1*H*-1,2,3-triazole (**3d**)

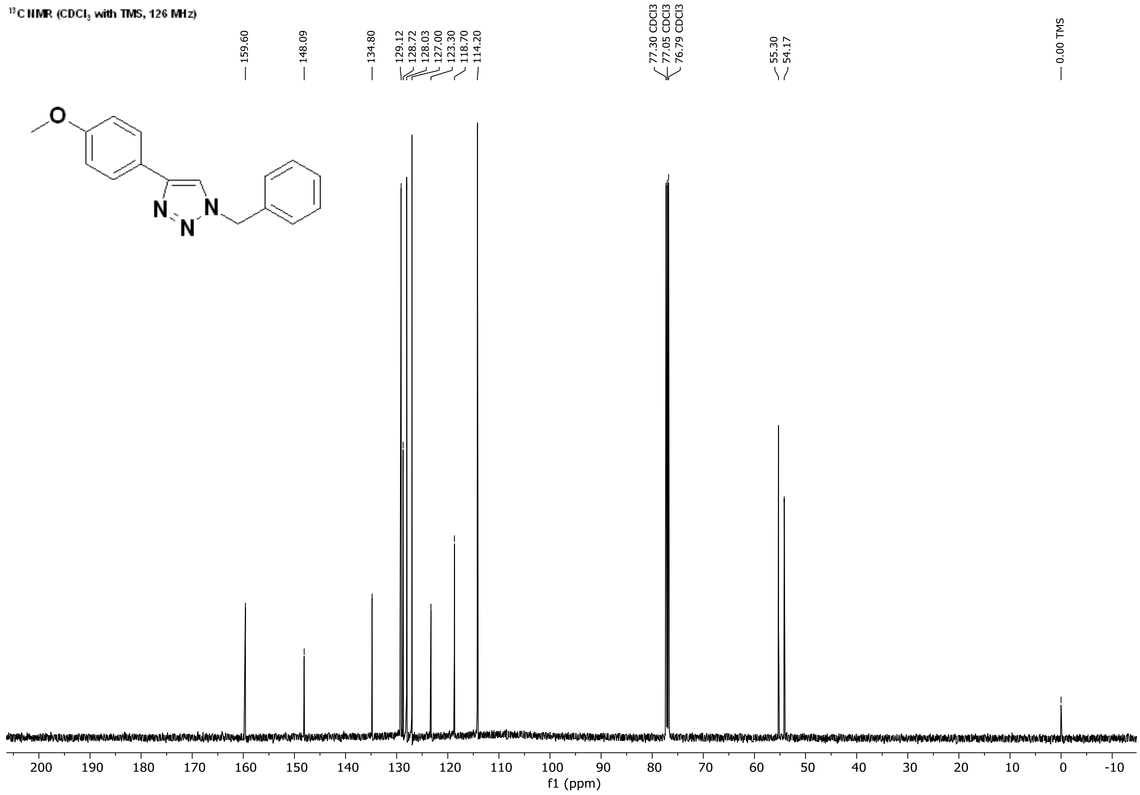

Figure S14. ^{13}C NMR spectrum of 1-benzyl-4-(4-methoxyphenyl)-1*H*-1,2,3-triazole (**3d**)

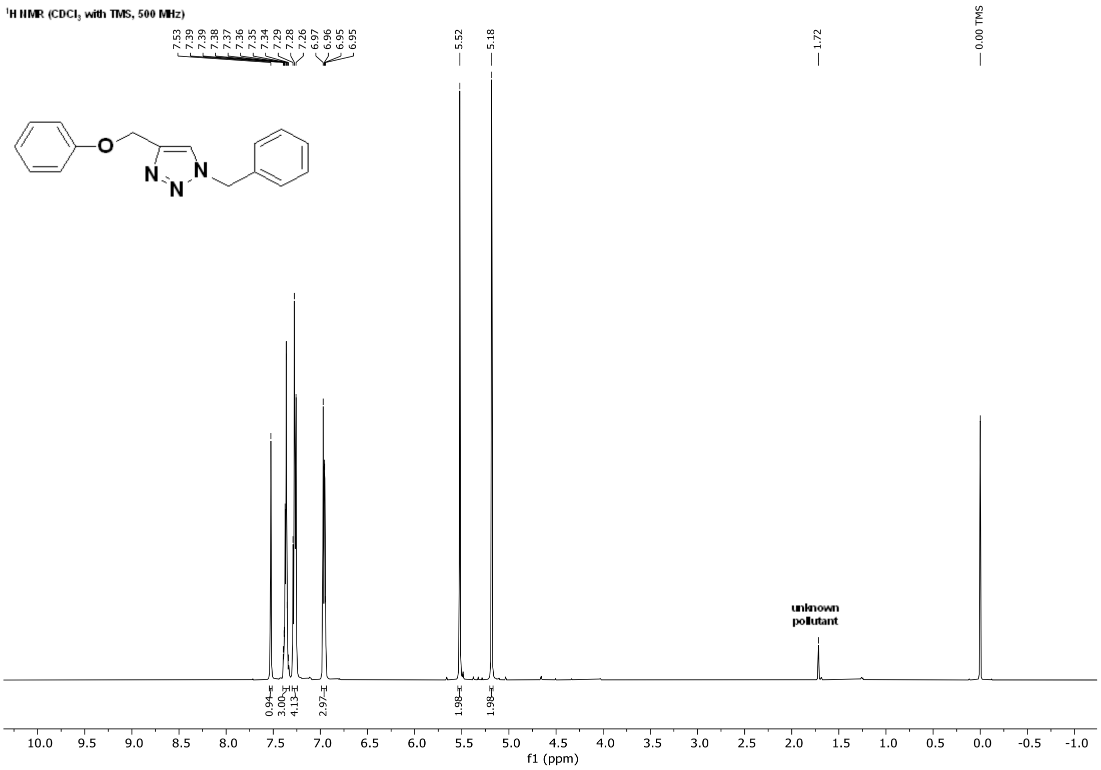

Figure S15. ^{1}H NMR spectrum of 1-benzyl-4-(phenoxymethyl)-1*H*-1,2,3-triazole (**3e**)

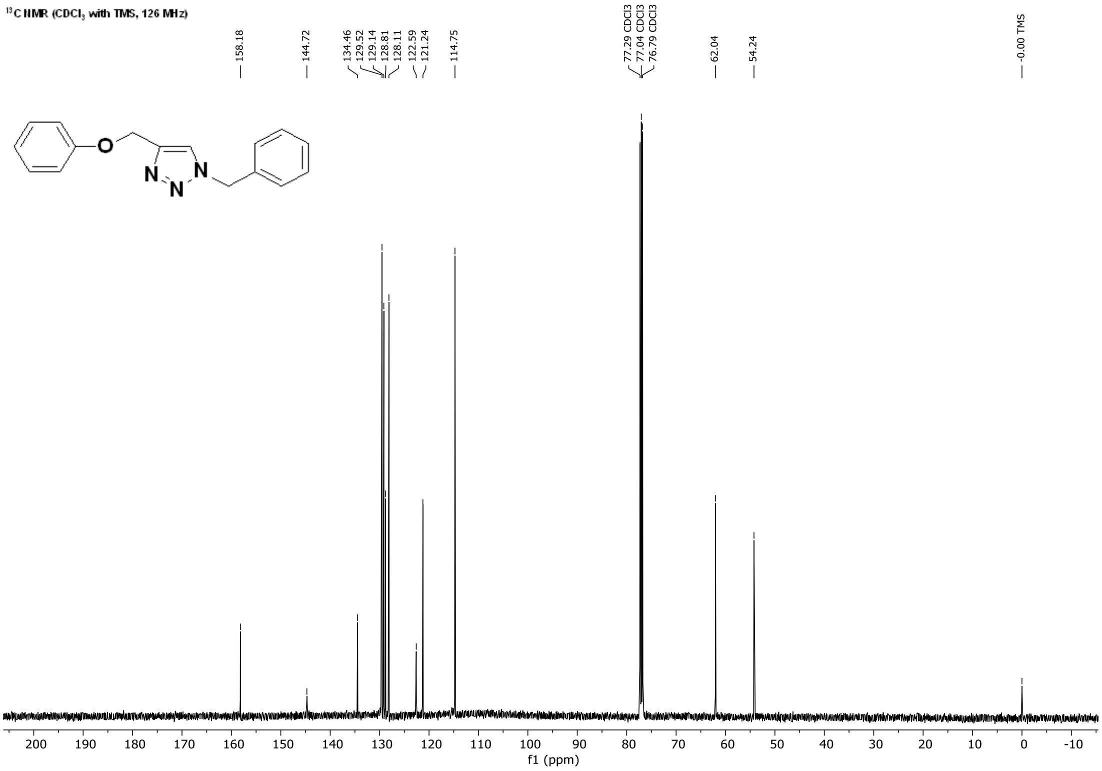

Figure S16. ^{13}C NMR spectrum of 1-benzyl-4-(phenoxymethyl)-1*H*-1,2,3-triazole (**3e**)

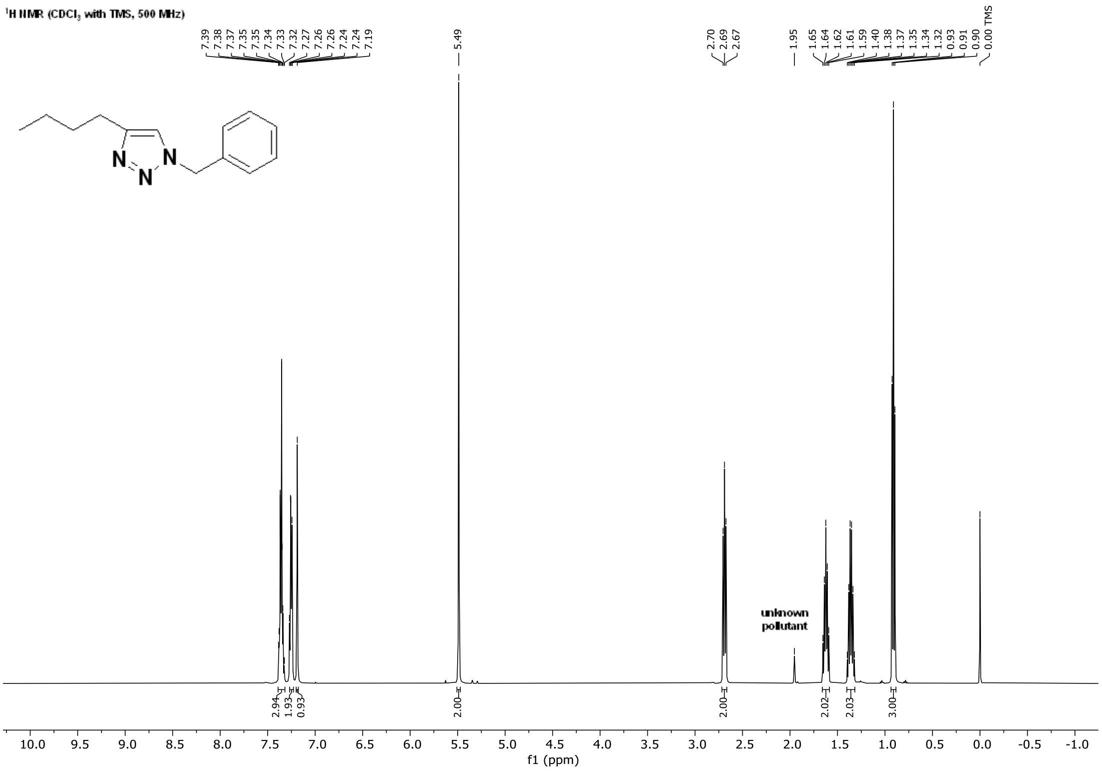

Figure S17. ^{1}H NMR spectrum of 1-benzyl-4-butyl-1*H*-1,2,3-triazole (**3f**)

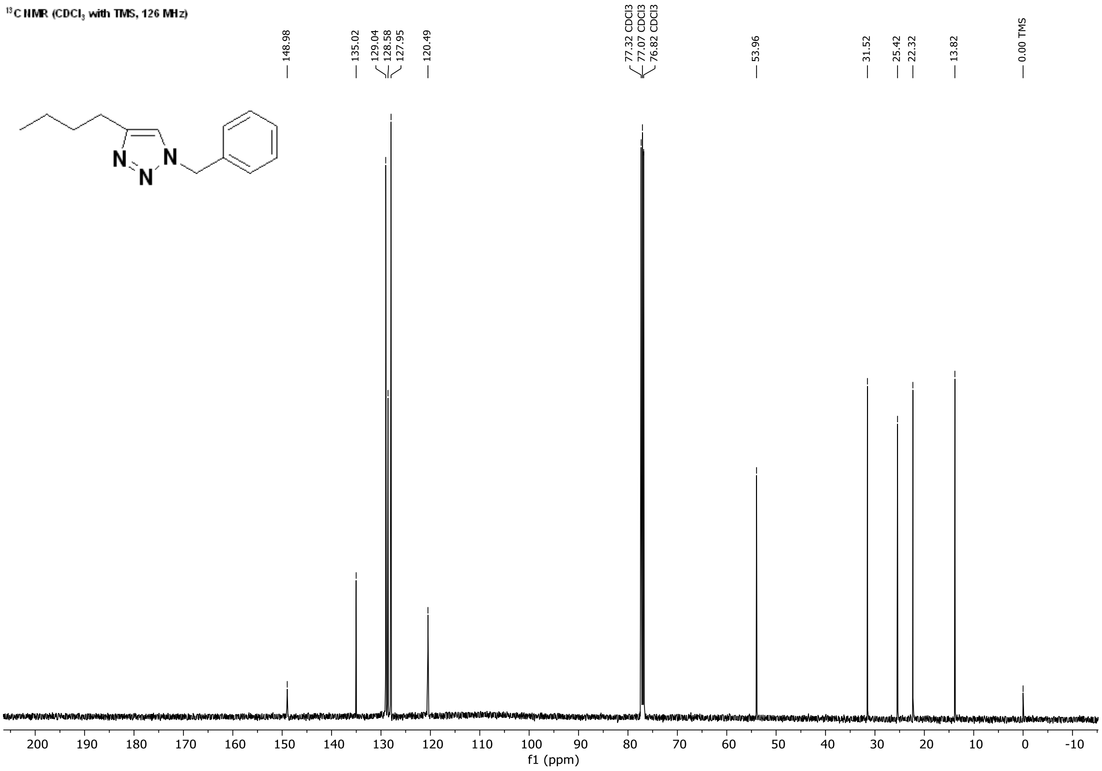

Figure S18. ^{13}C NMR of 1-benzyl-4-butyl-1*H*-1,2,3-triazole (**3f**)

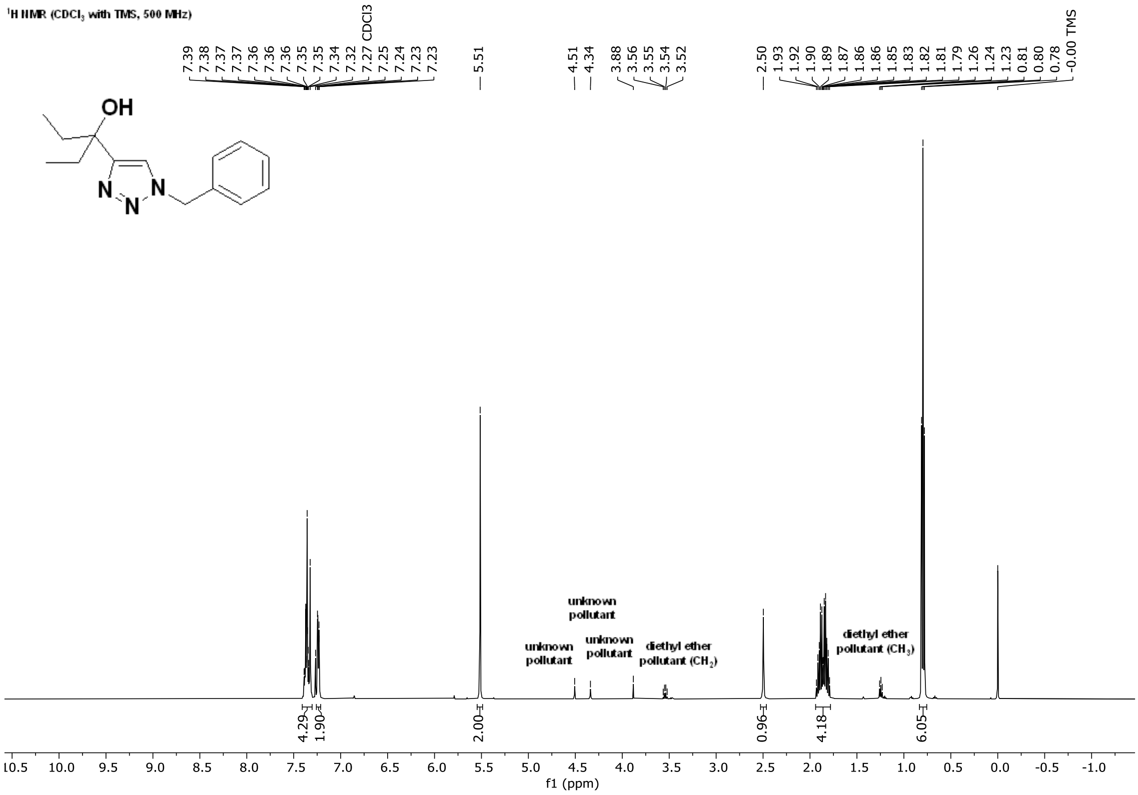

Figure S19. ^{1}H NMR spectrum of 3-(1-benzyl-1*H*-1,2,3-triazol-4-yl)pentan-3-ol (**3g**)

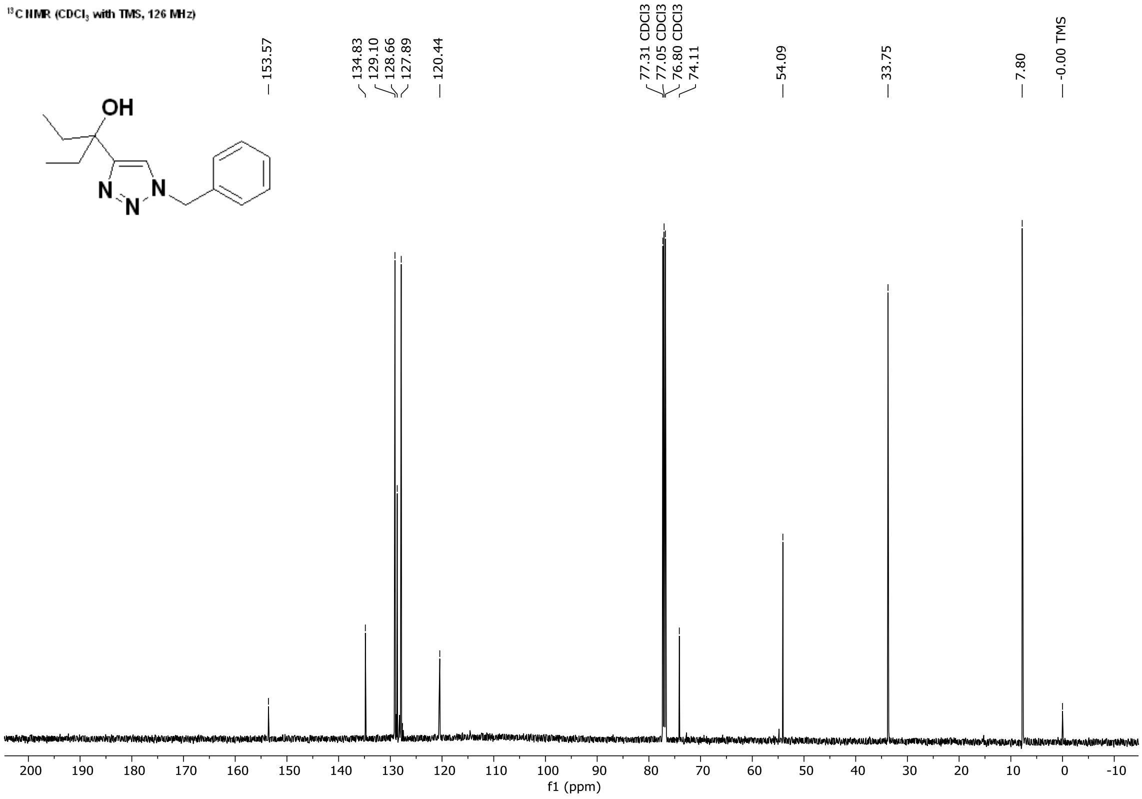

Figure S20. ^{13}C NMR spectrum of 3-(1-benzyl-1*H*-1,2,3-triazol-4-yl)pentan-3-ol (**3g**)

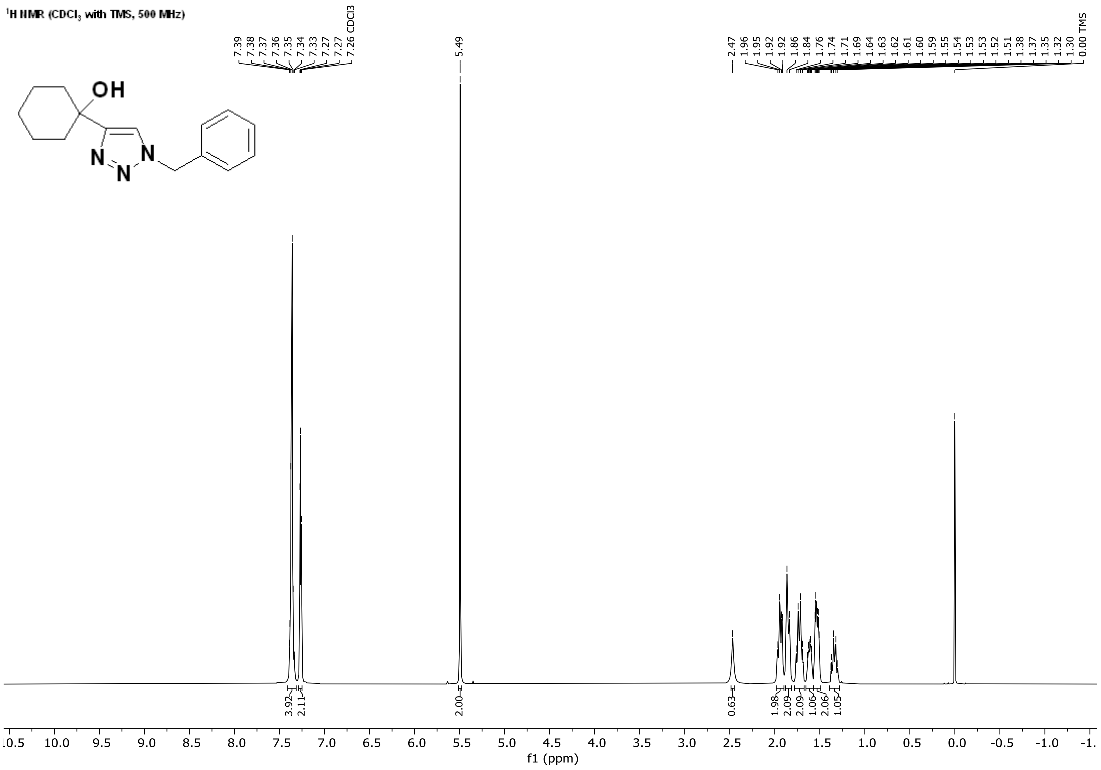

Figure S21. ^{1}H NMR spectrum of 1-(1-benzyl-1*H*-1,2,3-triazol-4-yl)cyclohexanol (**3h**)

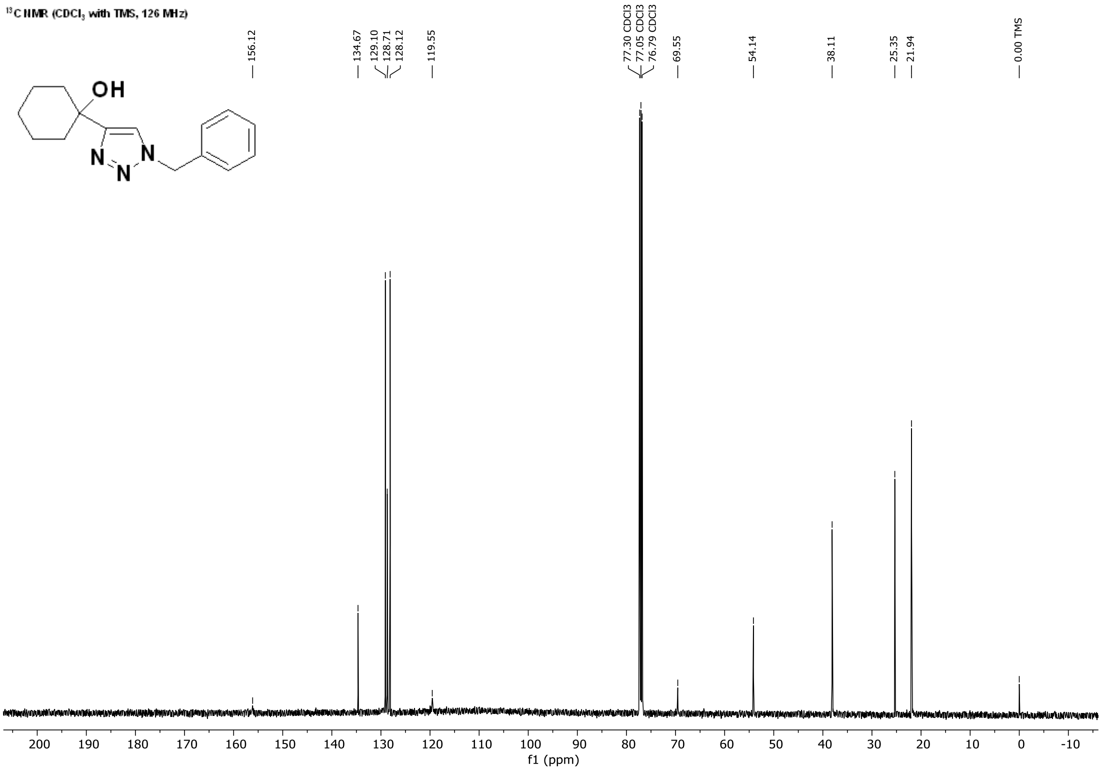

Figure S22. ^{13}C NMR spectrum of 1-(1-benzyl-1*H*-1,2,3-triazol-4-yl)cyclohexanol (**3h**)

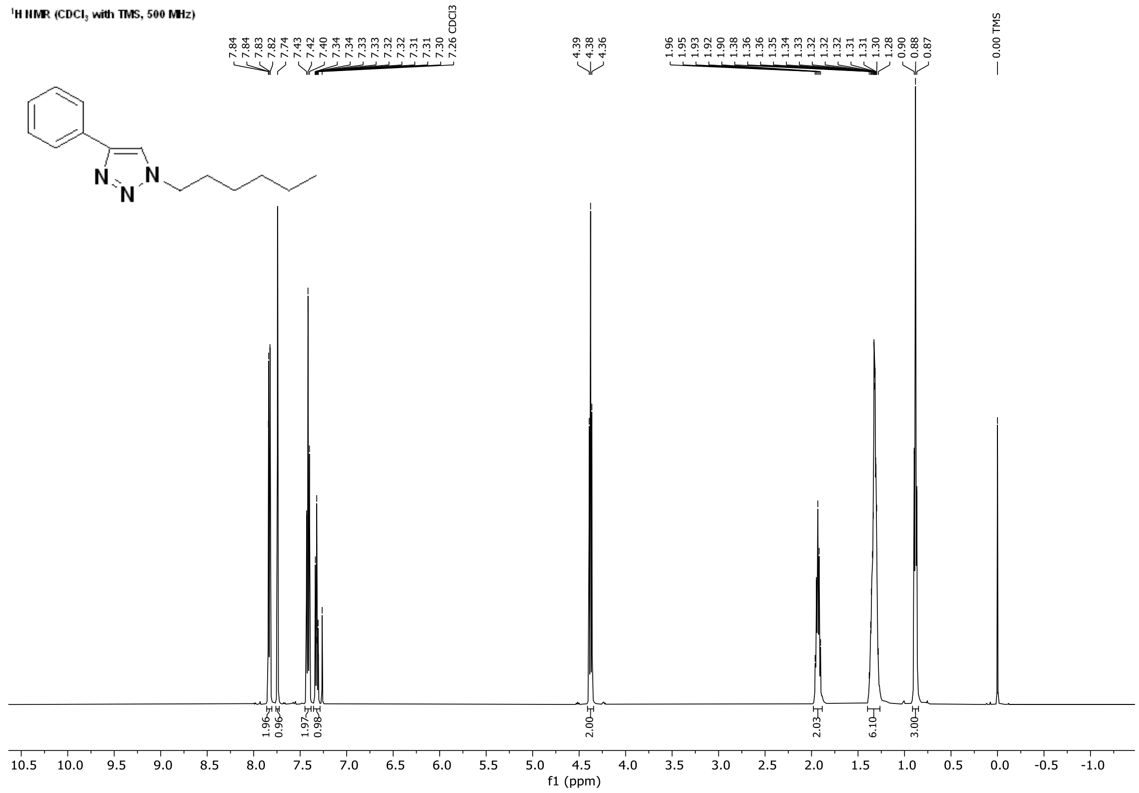

Figure S23. ^{1}H NMR spectrum of 1-hexyl-4-phenyl-1*H*-1,2,3-triazole (**5b**)

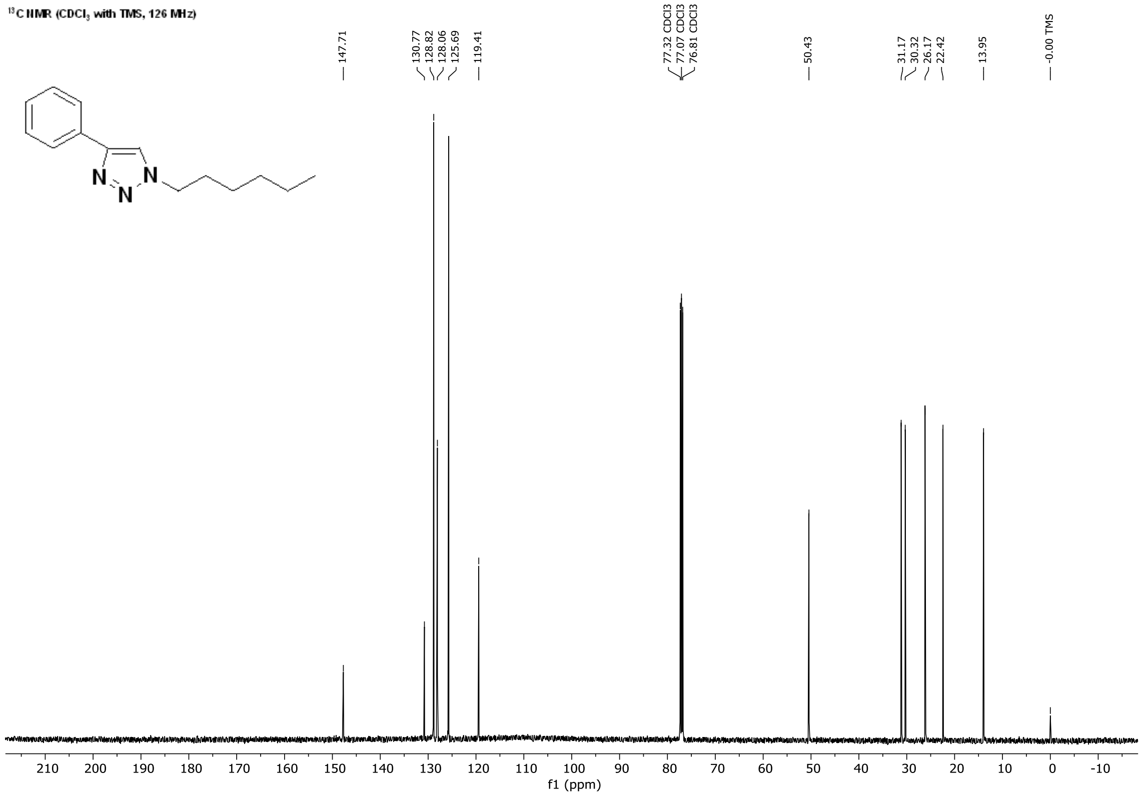

Figure S24. ^{13}C NMR spectrum of 1-hexyl-4-phenyl-1*H*-1,2,3-triazole (**5b**)

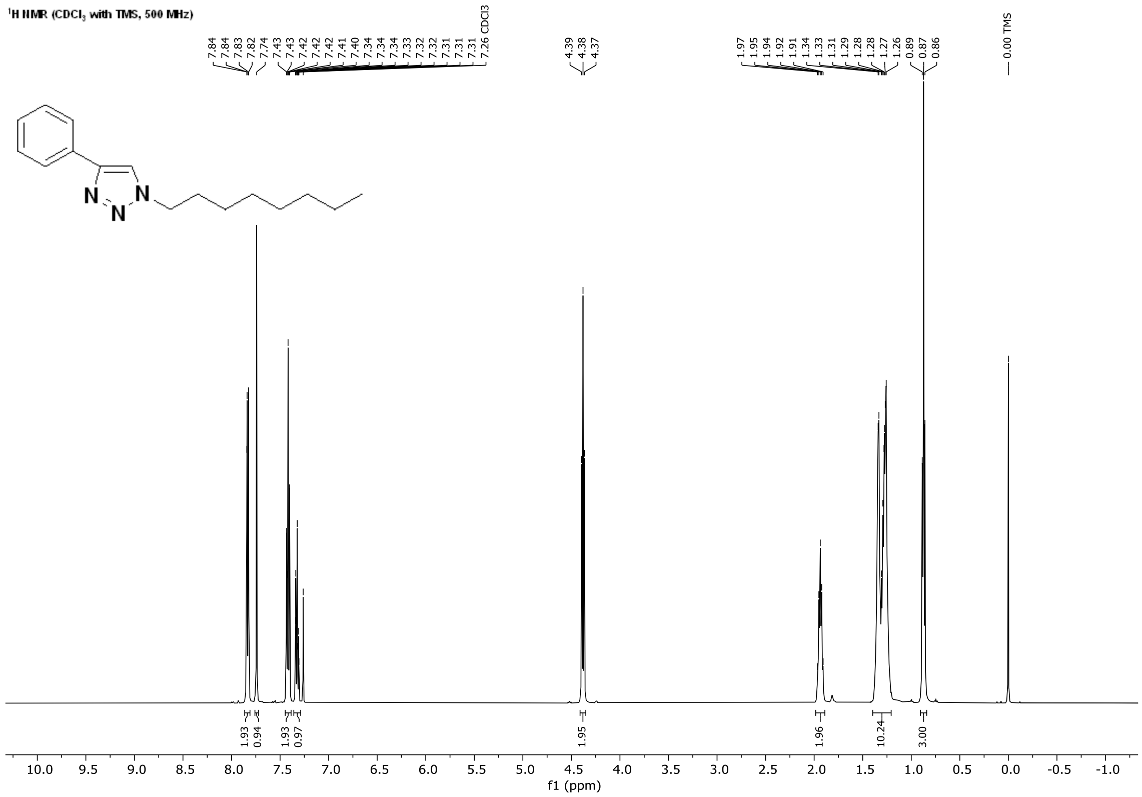

Figure S25. ^{1}H NMR spectrum of 1-octyl-4-phenyl-1*H*-1,2,3-triazole (**5c**)

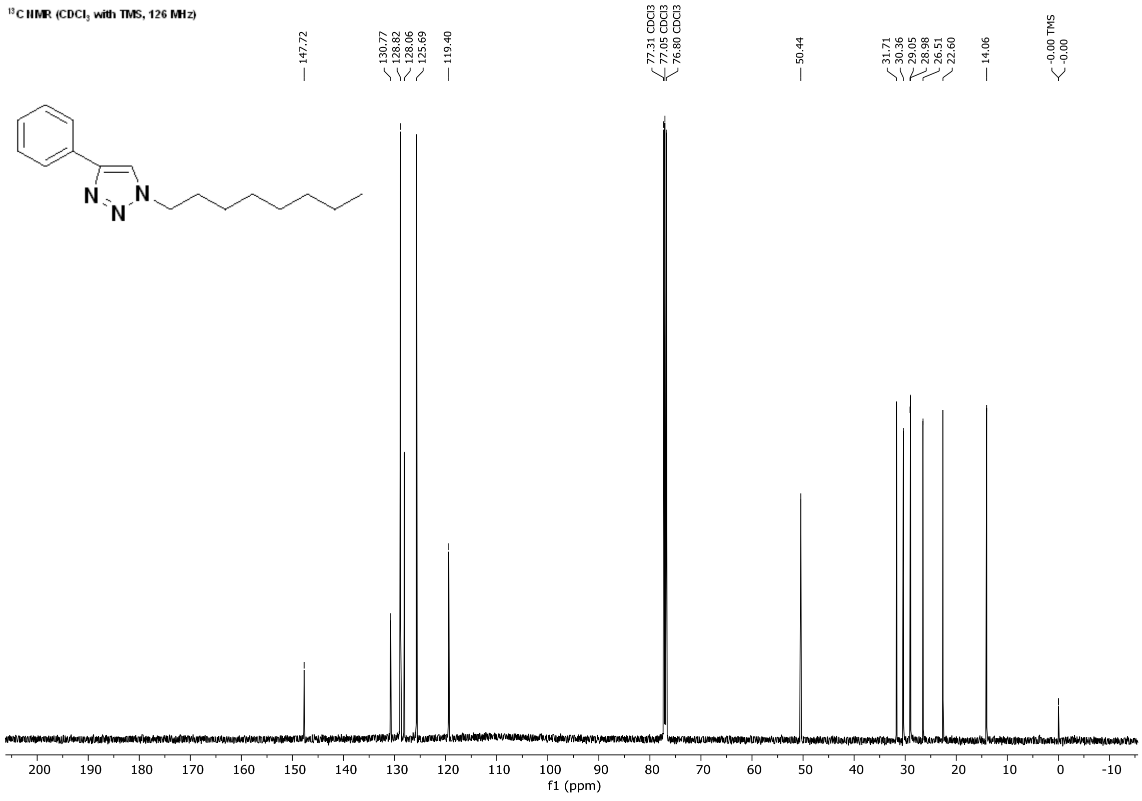

Figure S26. ^{13}C NMR spectrum of 1-octyl-4-phenyl-1*H*-1,2,3-triazole (**5c**)

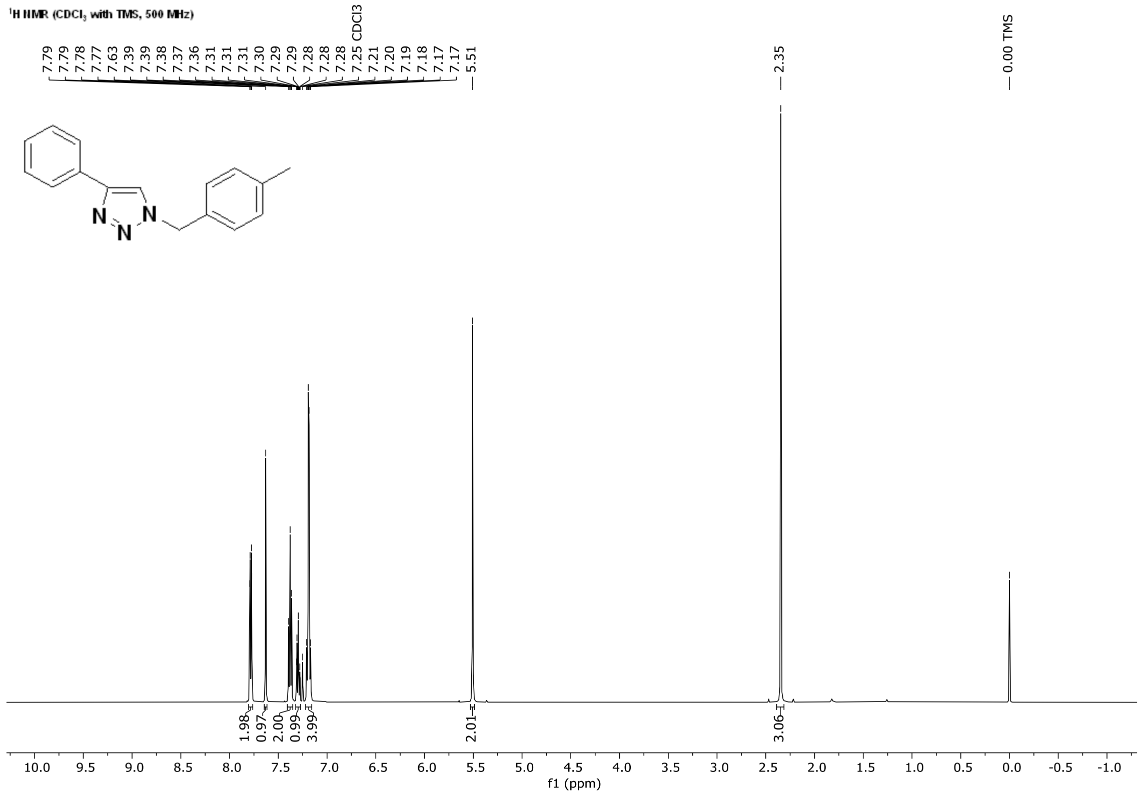

Figure S27. ^{1}H NMR spectrum of 1-(4-methylbenzyl)-4-phenyl-1*H*-1,2,3-triazole (**5d**)

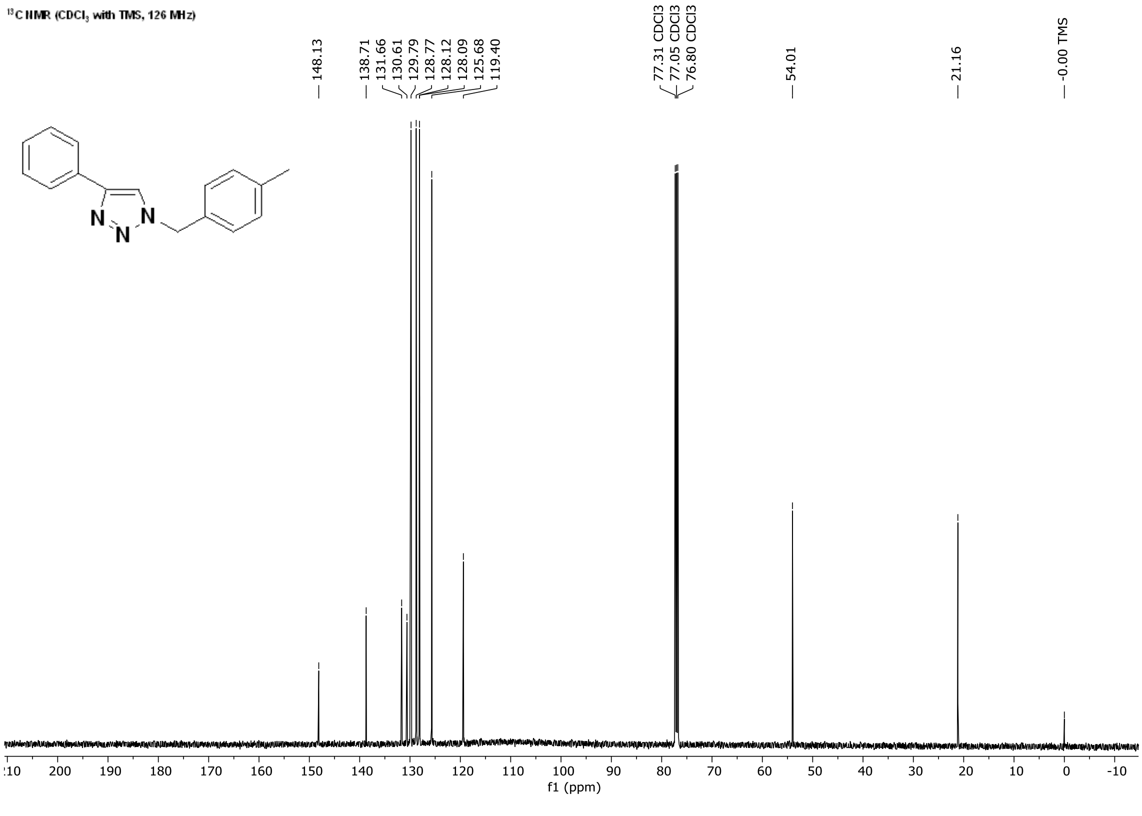

Figure S28. ^{13}C NMR spectrum of 1-(4-methylbenzyl)-4-phenyl-1*H*-1,2,3-triazole (**5d**)

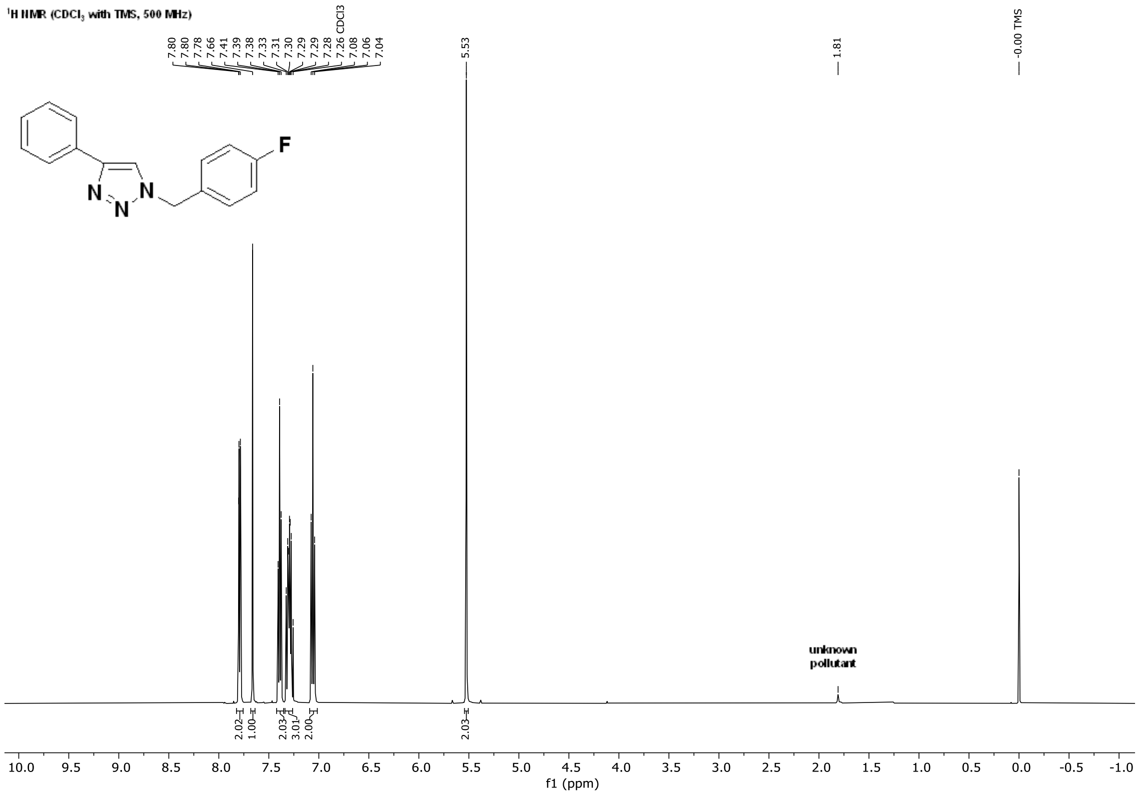

Figure S29. ^{1}H NMR spectrum of 1-(4-fluorobenzyl)-4-phenyl-1*H*-1,2,3-triazole (**5e**)

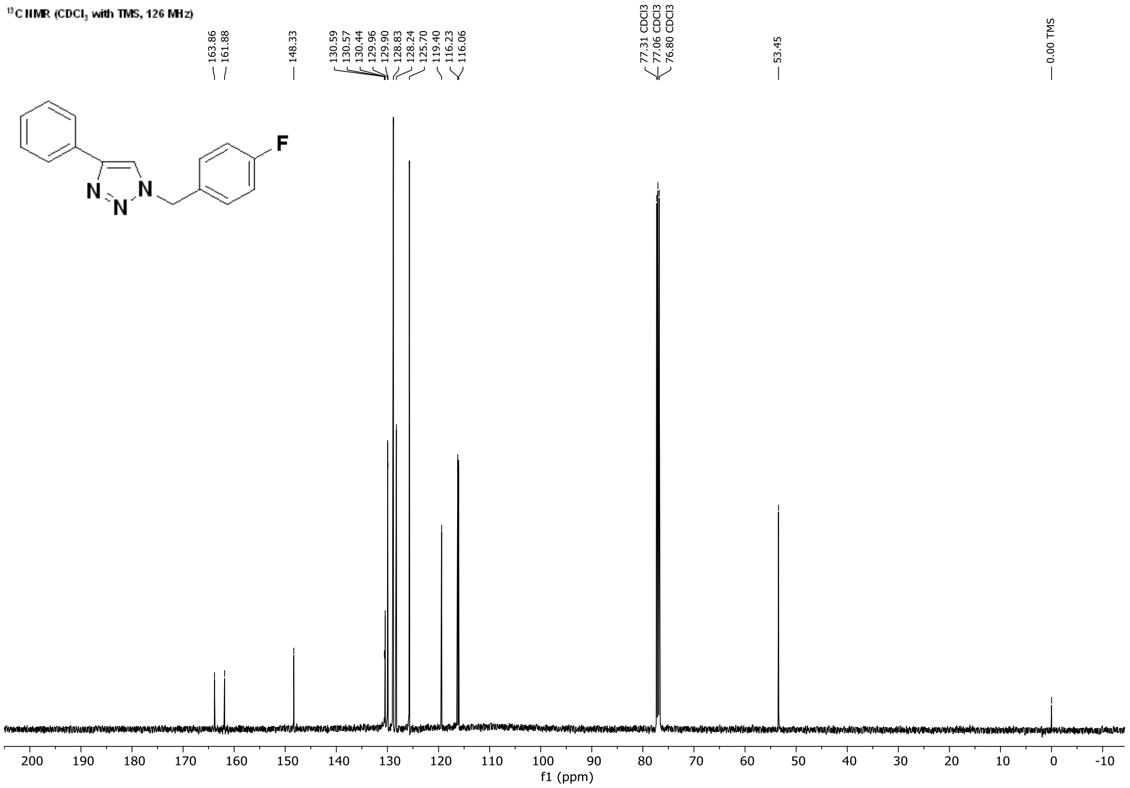

Figure S30. ^{13}C NMR spectrum of 1-(4-fluorobenzyl)-4-phenyl-1*H*-1,2,3-triazole (**5e**)

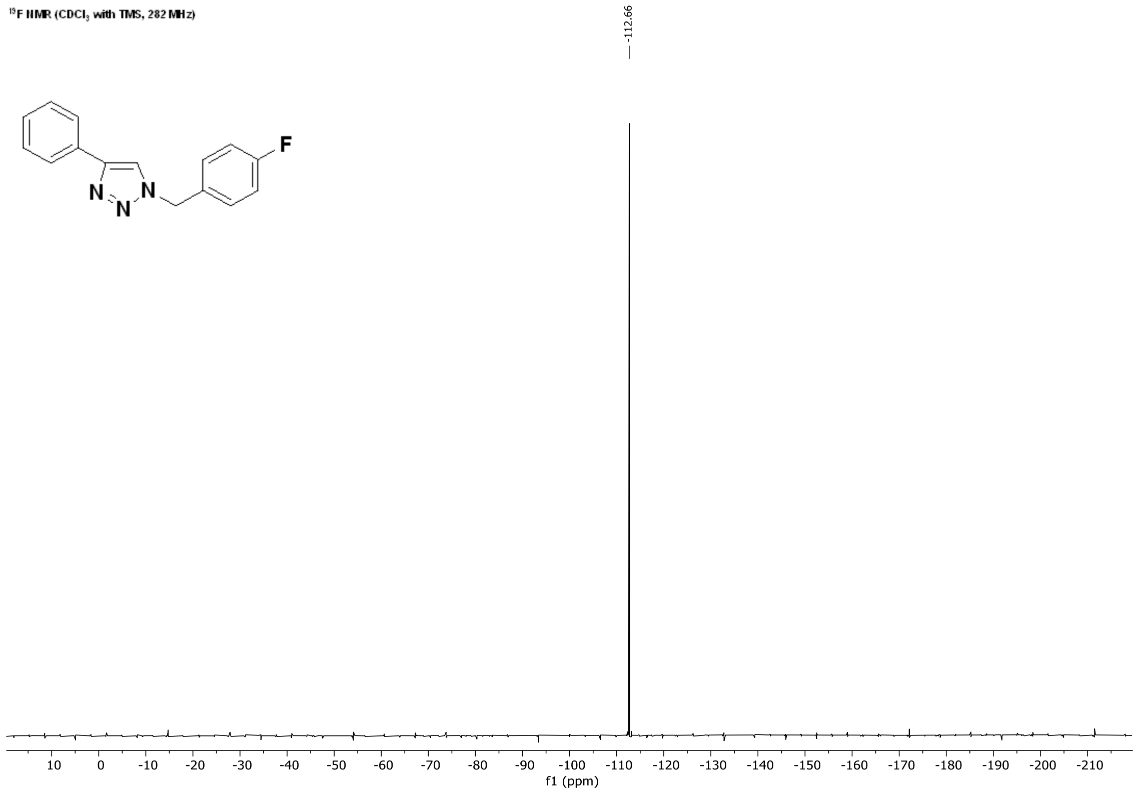

Figure S31. ^{19}F NMR spectrum of 1-(4-fluorobenzyl)-4-phenyl-1*H*-1,2,3-triazole (**5e**)

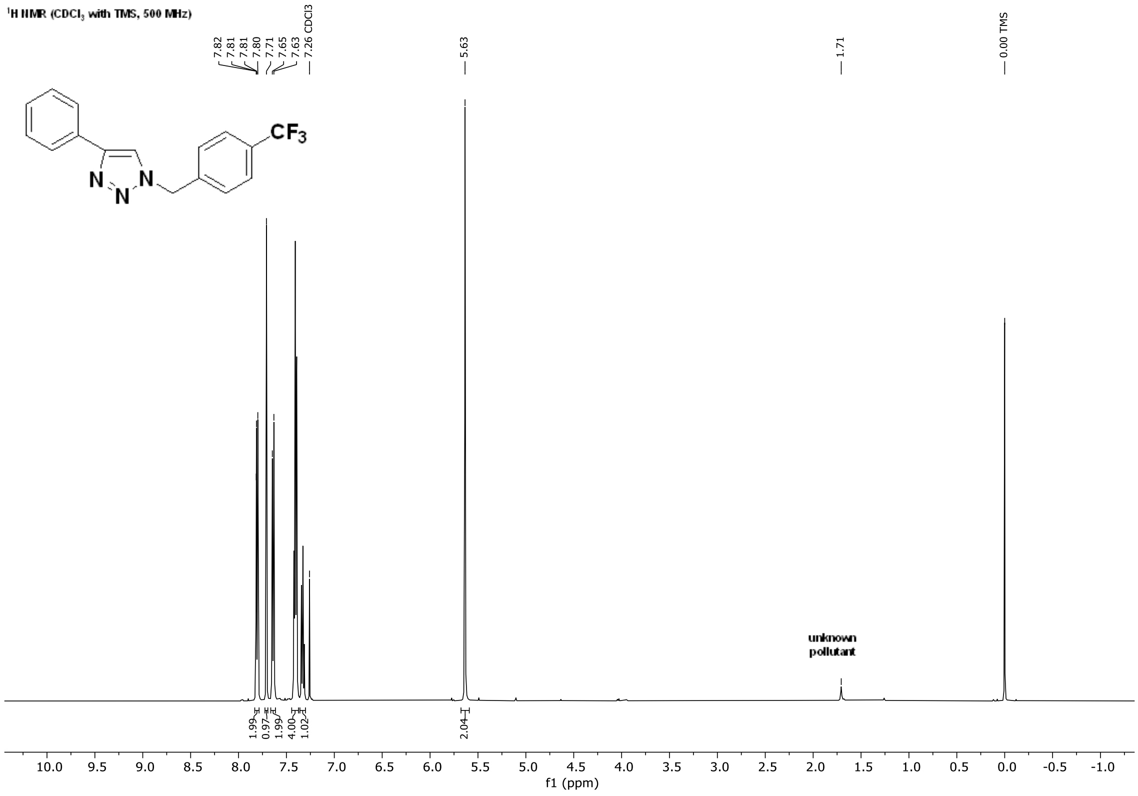

Figure S32. ^{1}H NMR spectrum of 4-phenyl-1-(4-(trifluoromethyl)benzyl)-1*H*-1,2,3-triazole (**5f**)

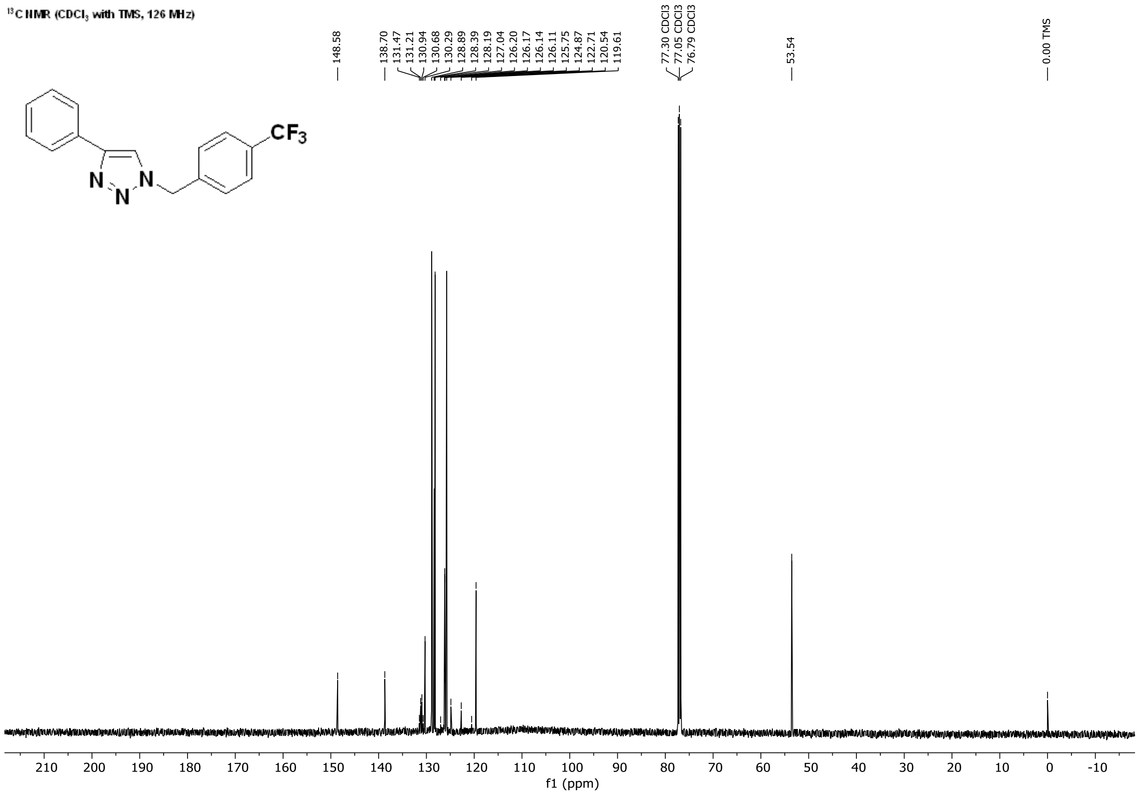

Figure S33. ^{13}C NMR spectrum of 4-phenyl-1-(4-(trifluoromethyl)benzyl)-1*H*-1,2,3-triazole (**5f**)

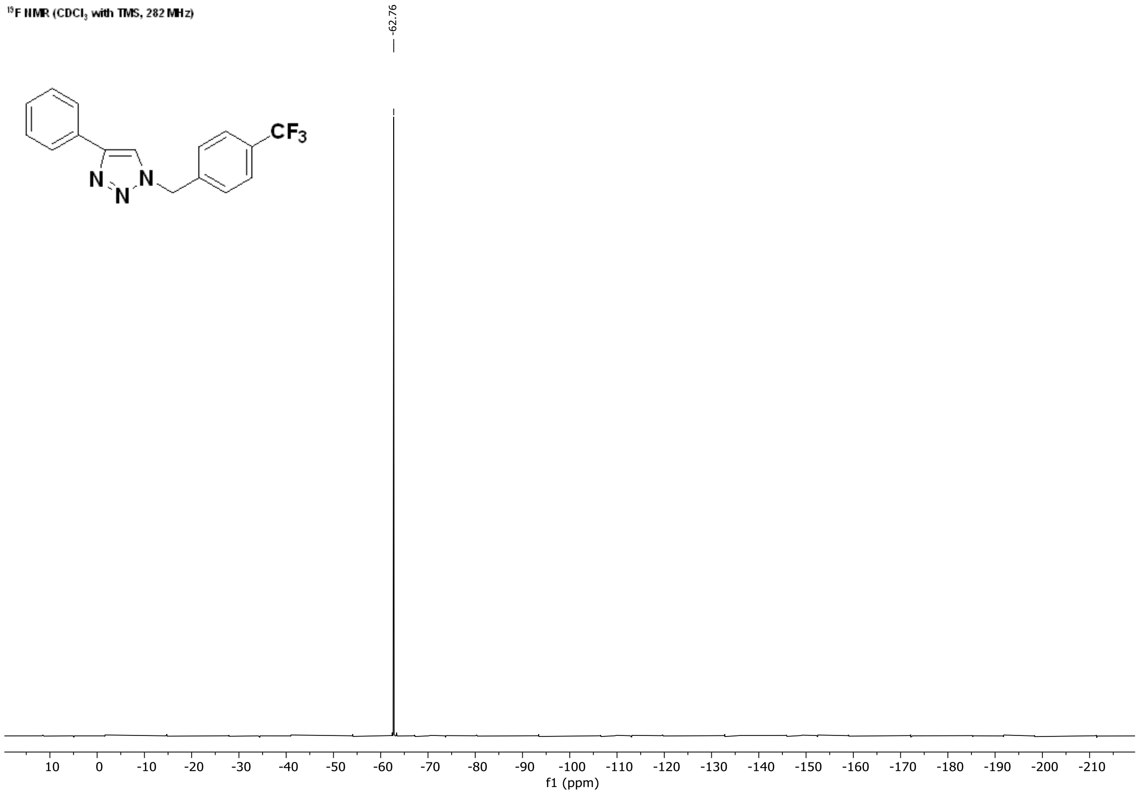

Figure S34. ^{19}F NMR spectrum of 4-phenyl-1-(4-(trifluoromethyl)benzyl)-1*H*-1,2,3-triazole (**5f**)

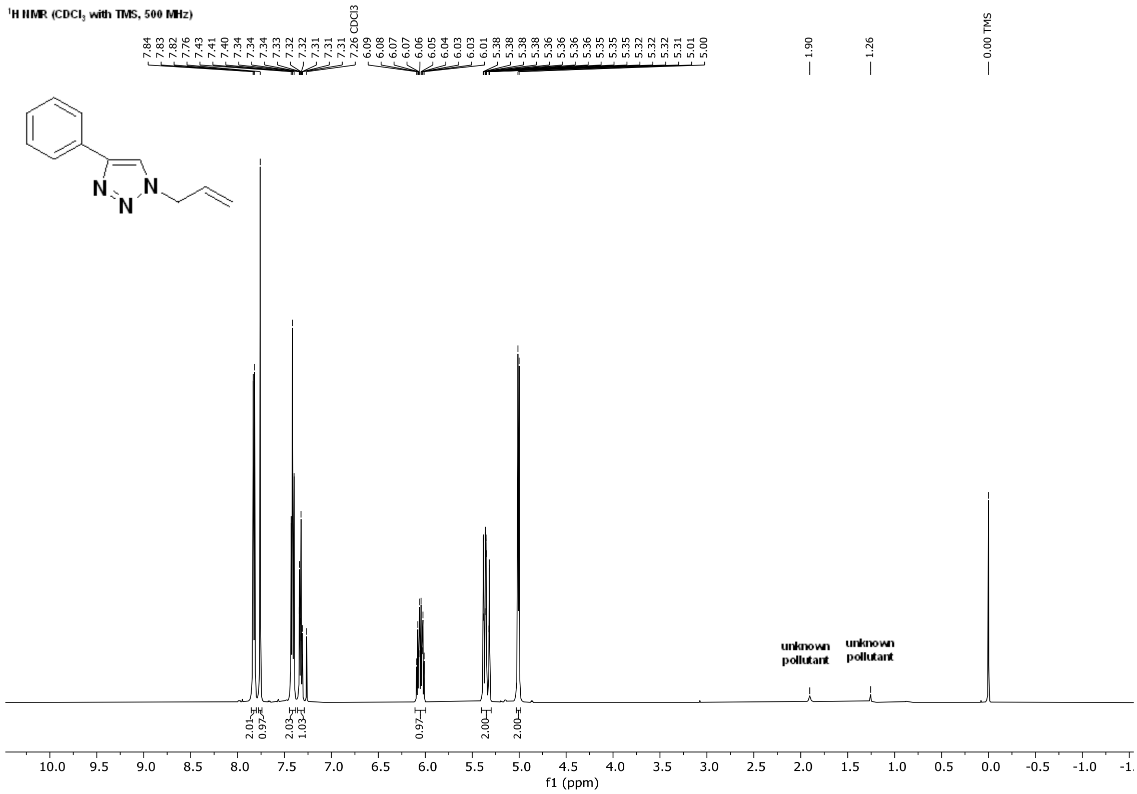

Figure S35. ^{1}H NMR spectrum of 1-allyl-4-phenyl-1*H*-1,2,3-triazole (**6a**)

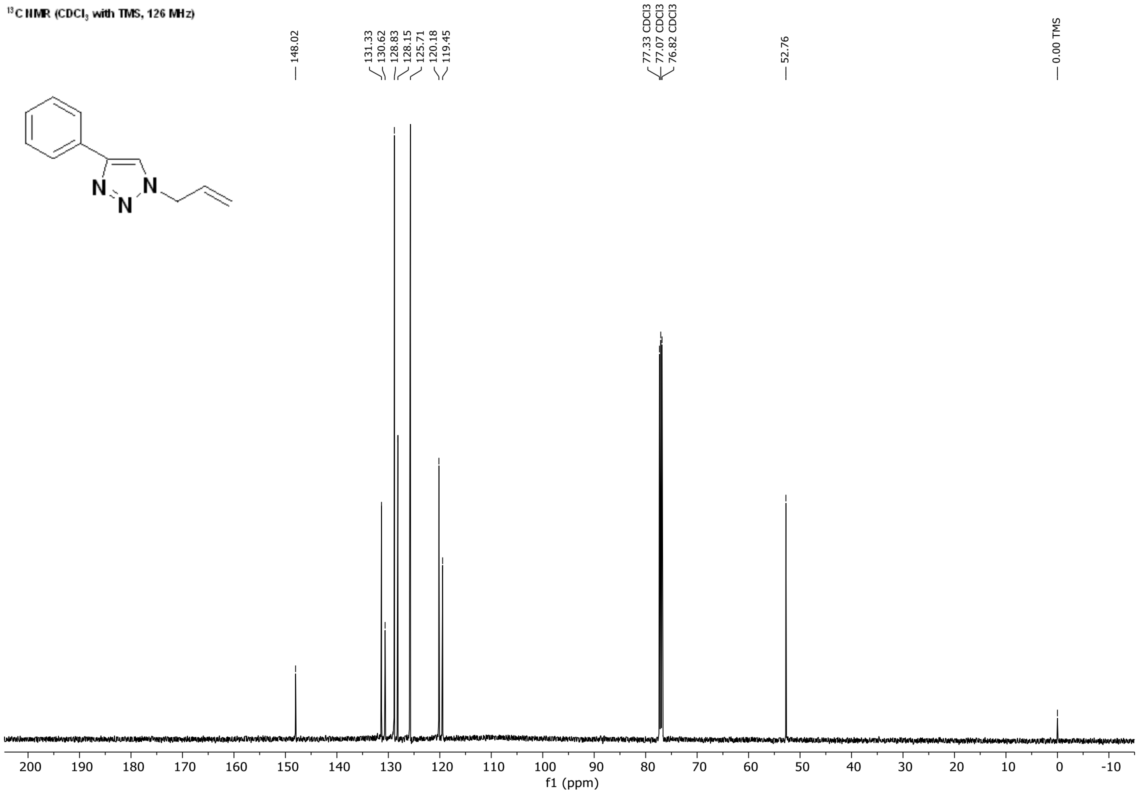

Figure S36. ^{13}C NMR spectrum of 1-allyl-4-phenyl-1*H*-1,2,3-triazole (**6a**)

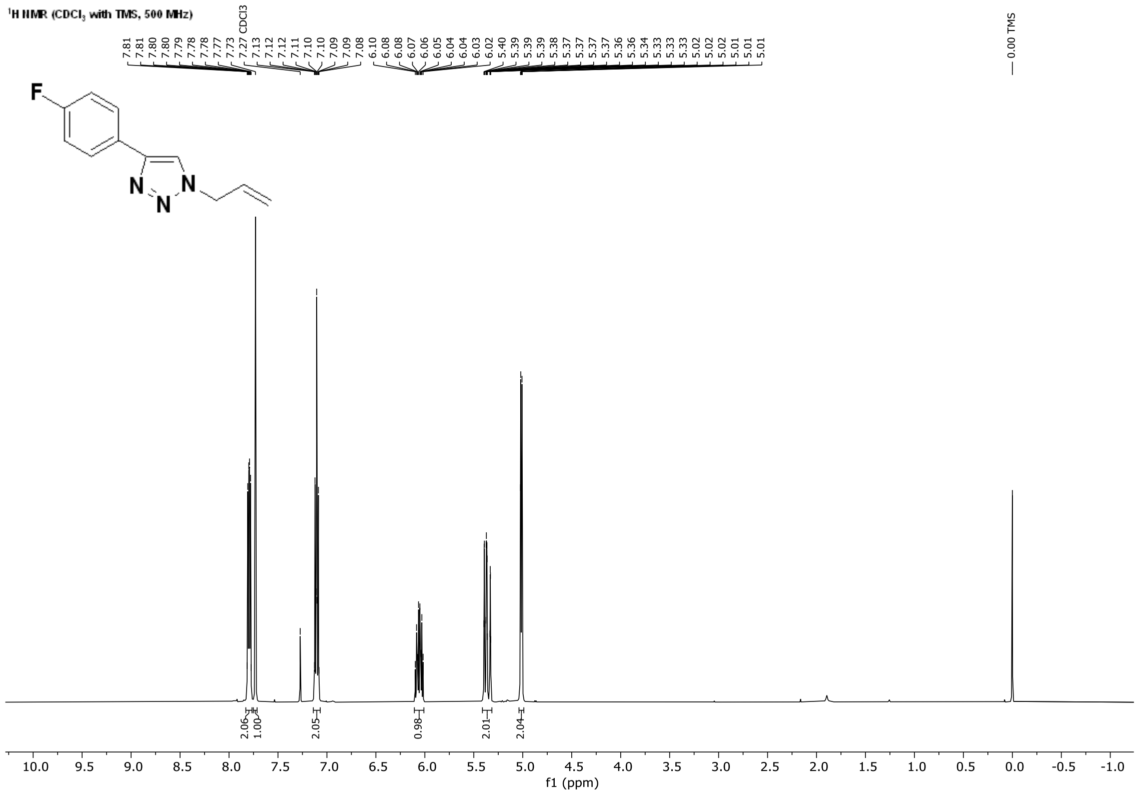

Figure S37. ^{1}H NMR spectrum of 1-allyl-4-(4-fluorophenyl)-1*H*-1,2,3-triazole (**6b**)

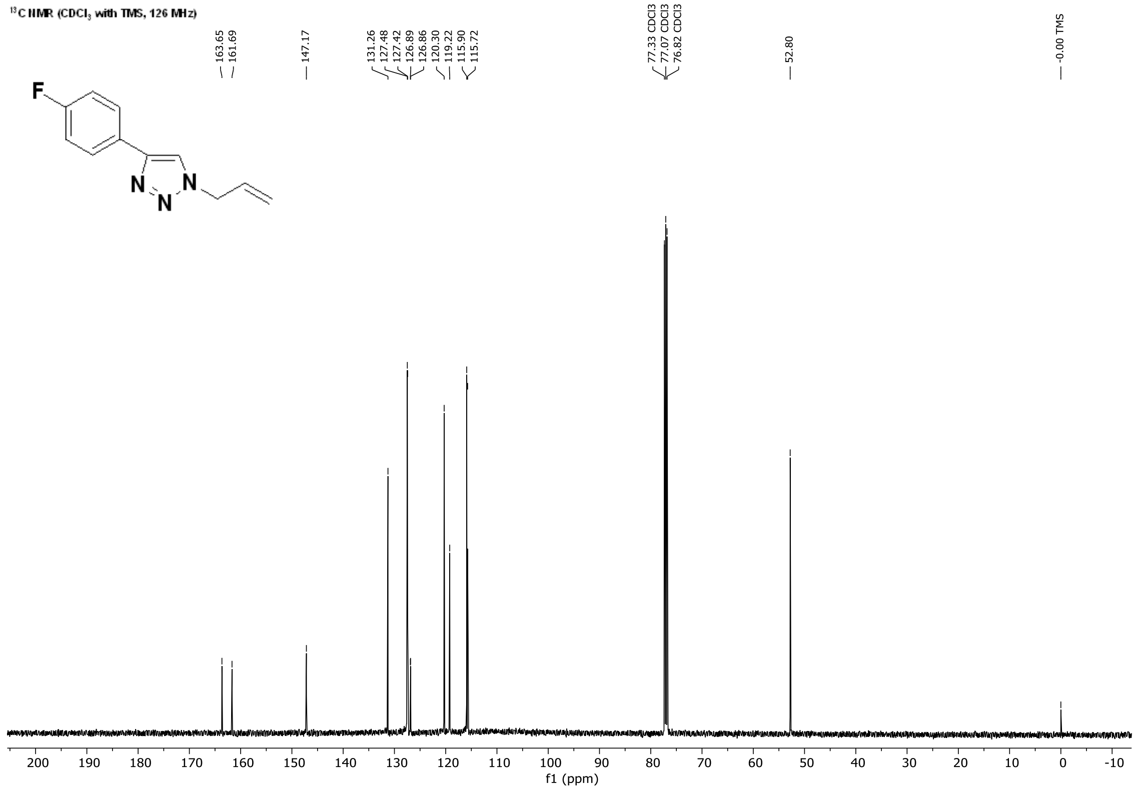

Figure S38. ^{13}C NMR spectrum of 1-allyl-4-(4-fluorophenyl)-1*H*-1,2,3-triazole (**6b**)

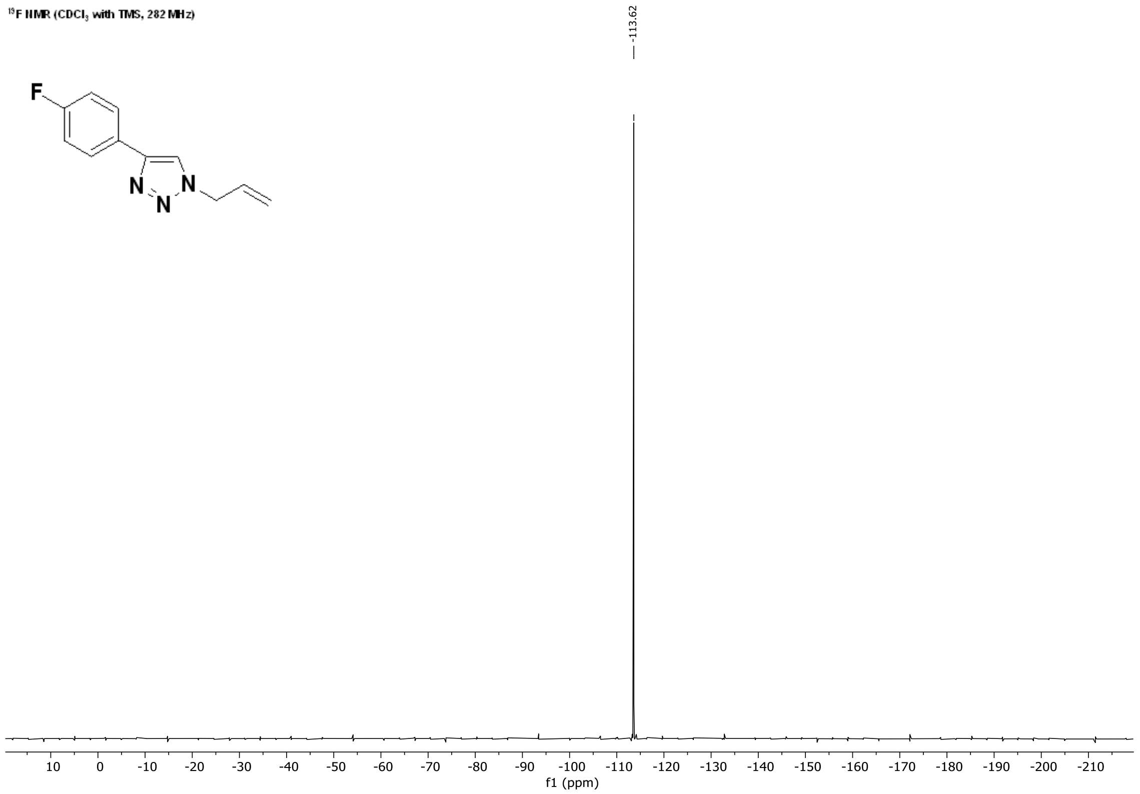

Figure S39. ^{19}F NMR spectrum of 1-allyl-4-(4-fluorophenyl)-1*H*-1,2,3-triazole (**6b**)

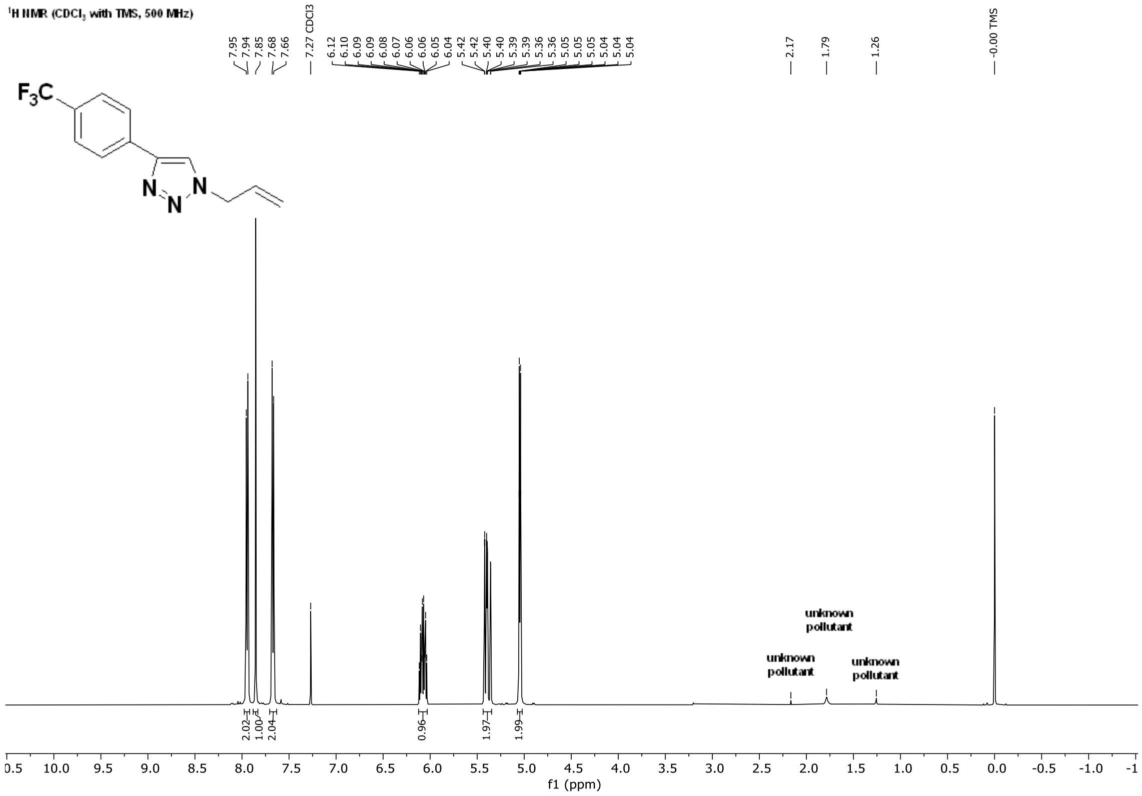

Figure S40. ^{1}H NMR spectrum of 1-allyl-4-(4-(trifluoromethyl)phenyl)-1*H*-1,2,3-triazole (**6c**)

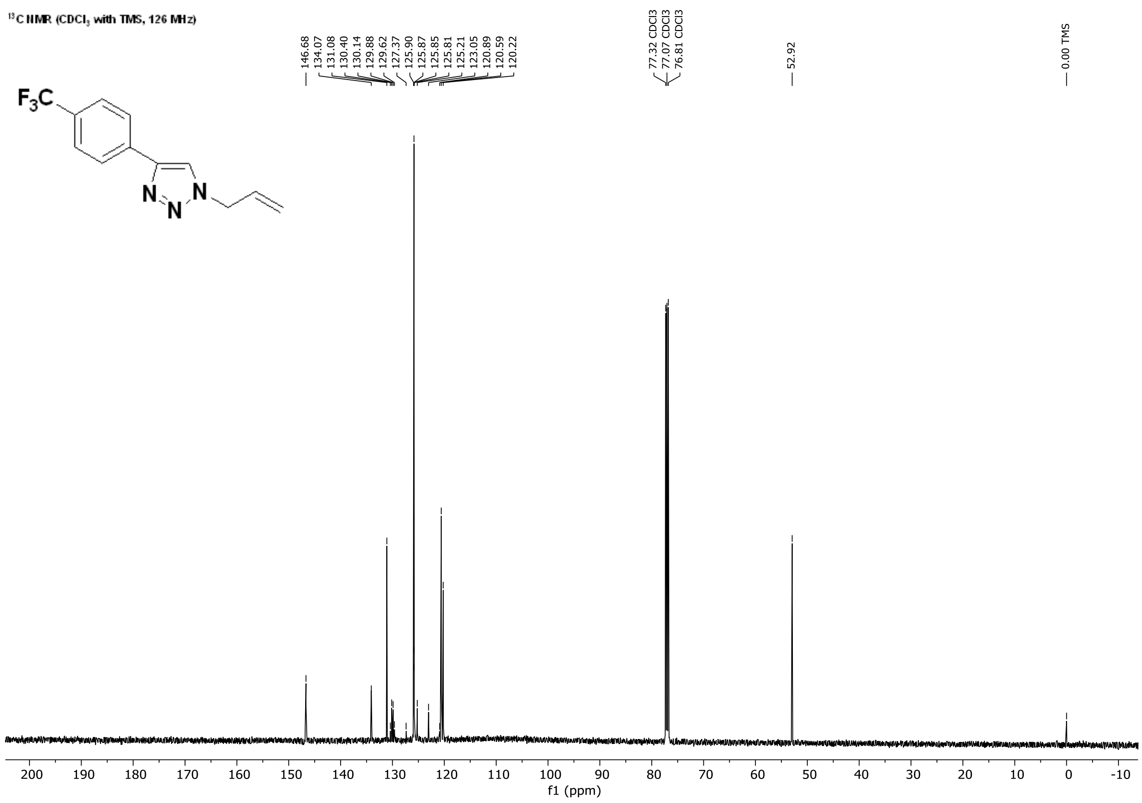

Figure S41. ^{13}C NMR spectrum of 1-allyl-4-(4-(trifluoromethyl)phenyl)-1*H*-1,2,3-triazole (**6c**)

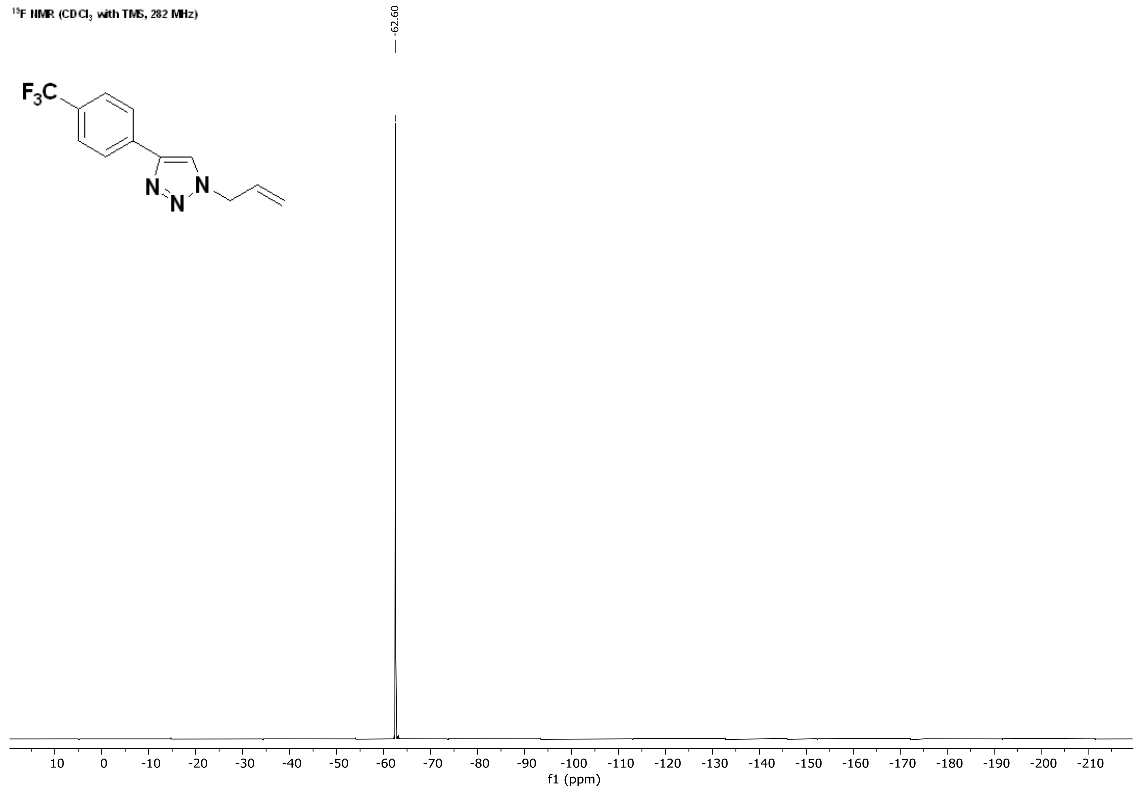

Figure S42. ^{19}F NMR spectrum of 1-allyl-4-(4-(trifluoromethyl)phenyl)-1*H*-1,2,3-triazole (**6c**)

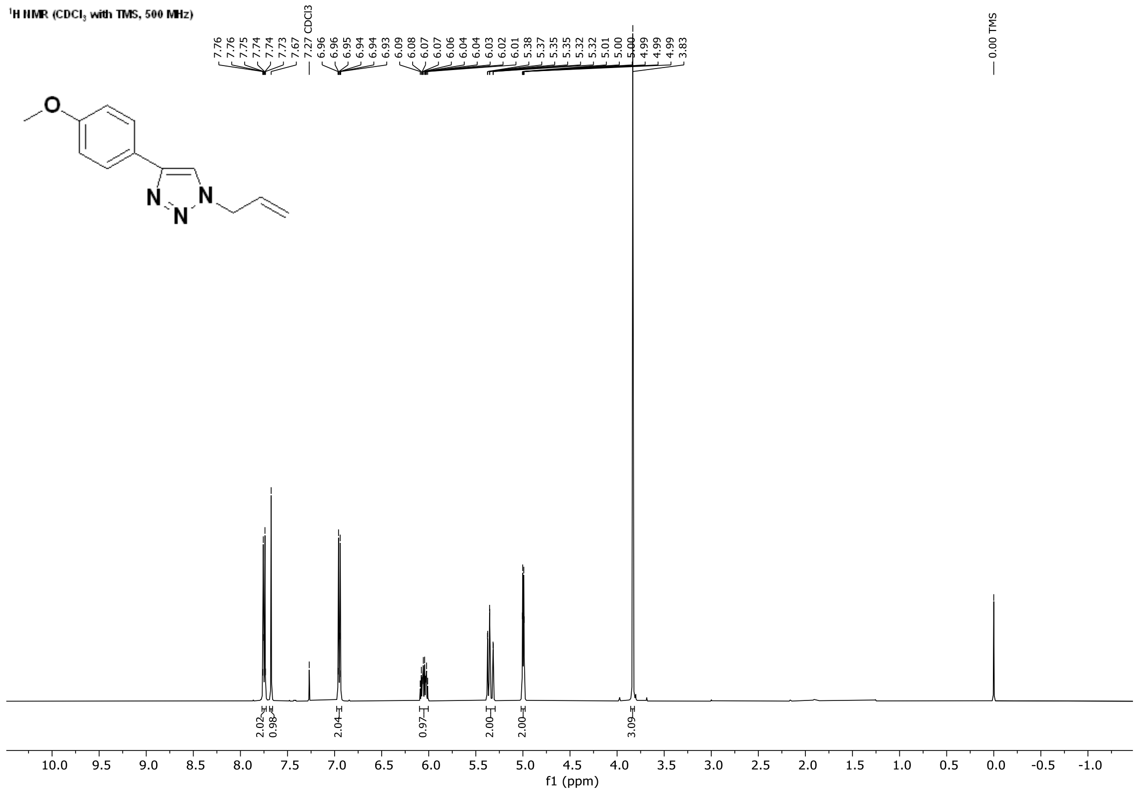

Figure S43. ^{1}H NMR spectrum of 1-allyl-4-(4-methoxyphenyl)-1*H*-1,2,3-triazole (**6d**)

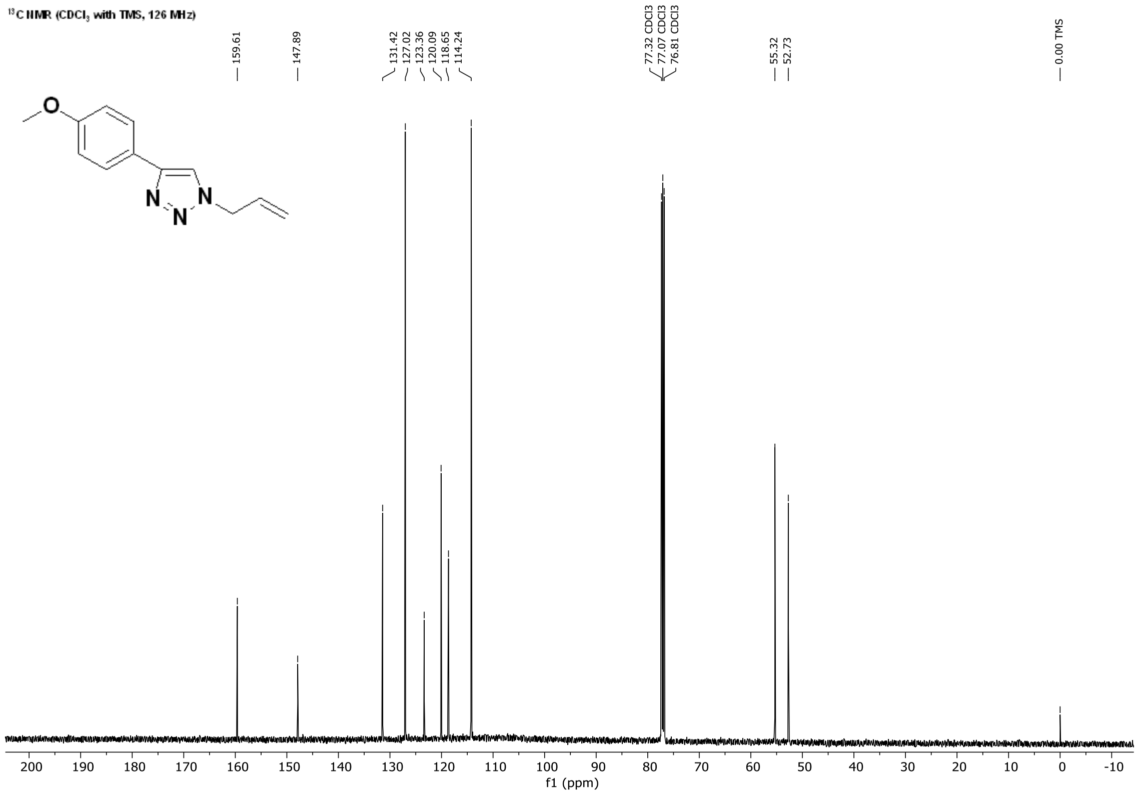

Figure S44. ^{13}C NMR spectrum of 1-allyl-4-(4-methoxyphenyl)-1*H*-1,2,3-triazole (**6d**)

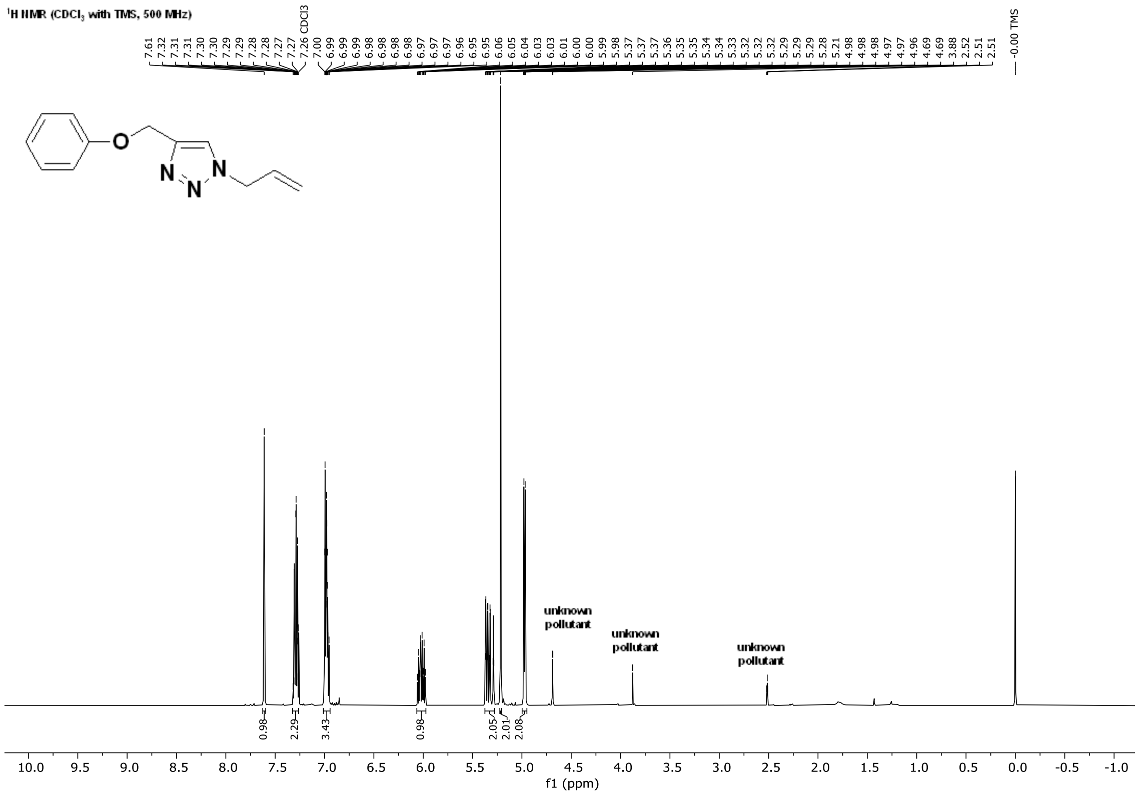

Figure S45. ^{1}H NMR spectrum of 1-allyl-4-(phenoxymethyl)-1*H*-1,2,3-triazole (**6e**)

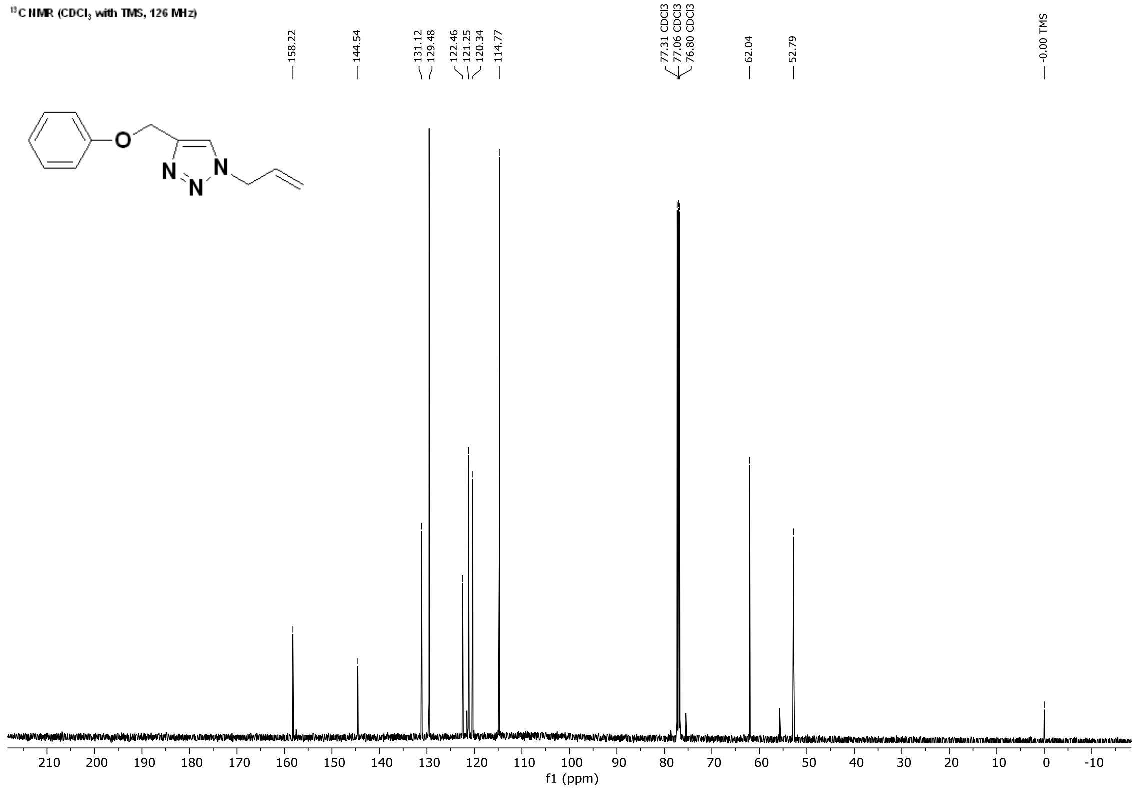

Figure S46. ^{13}C NMR spectrum of 1-allyl-4-(phenoxymethyl)-1*H*-1,2,3-triazole (**6e**)

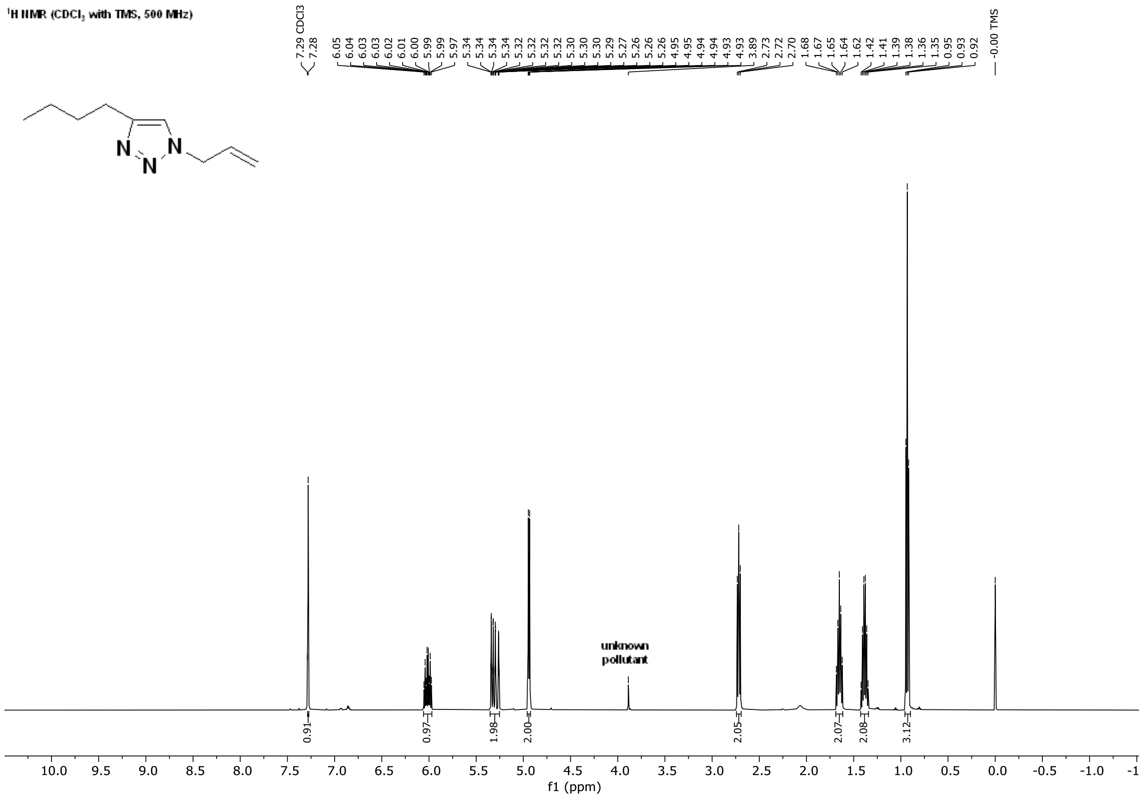

Figure S47. ^{1}H NMR spectrum of 1-allyl-4-butyl-1*H*-1,2,3-triazole (**6f**)

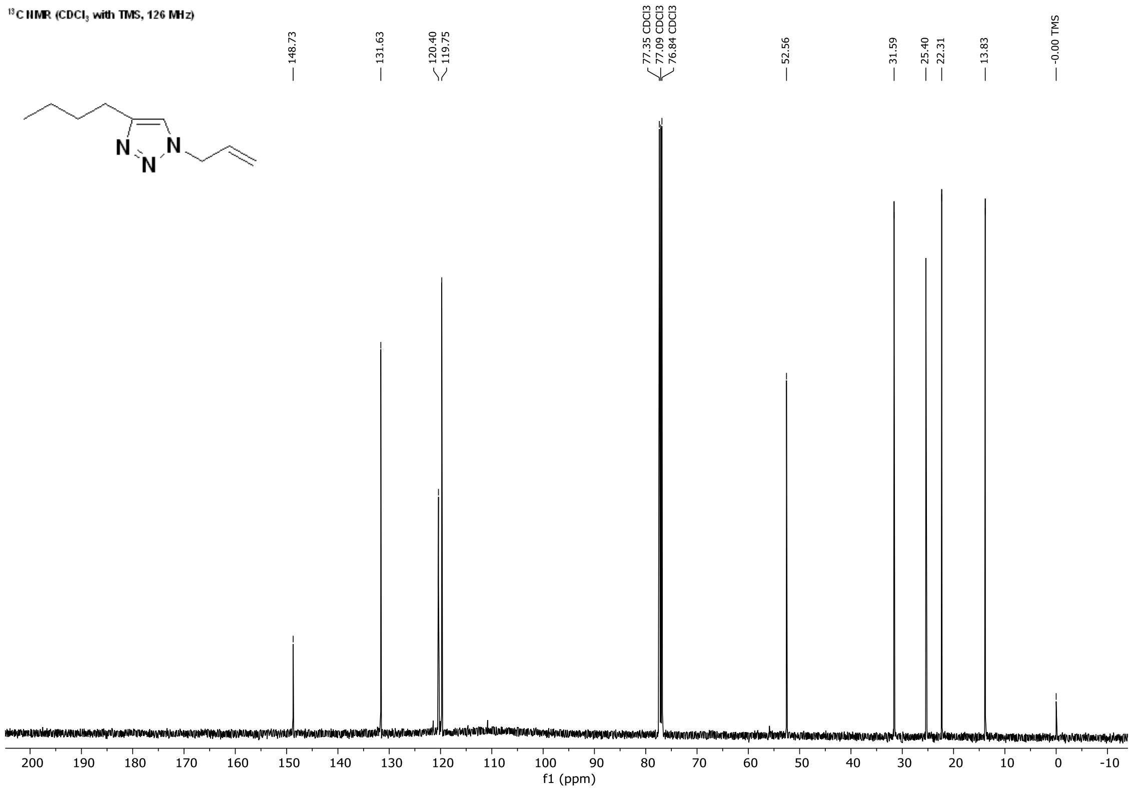

Figure S48. ^{13}C NMR spectrum of 1-allyl-4-butyl-1*H*-1,2,3-triazole (**6f**)

References

[1] Ju, Y.; Kumar, D.; Varma, R. S.; *J. Org. Chem*. **2006**, *71*, 17, 6697–6700. doi:10.1021/jo061114h

[2] Park, I. S.; Kwon, M. S.; Kim, Y.; Lee, J. S.; Park, J. Org. Lett. **2008**, *3*, 497–500. doi:10.1021/ol702790w

[3] Wen, J.; Wu, K.; Yang, D.; Tian, J.; Huang, Z.; Filatov, A. S.; Lei, A.; Lin X.-M. *ACS Appl. Mater. Interfaces*, **2018**, *10*, 25930–25935. doi:10.1021/acsami.8b06927

[4] Girard, C.; Önen, E.; Aufort, M.; Beauvière, S.; Samson, E.; Herscovici, J. *Org. Letters*, **2006**, *8*, 1689–1692. doi:10.1021/ol060283l

[5] Candelon, N.; Lastecoueres, D.; Diallo, A. K.; Ruiz Aranzaes, Astruc J.; D.; Vincent, J.-M. *Chem. Commun*. **2008**, 741-743. doi:10.1039/B716306A

[6] Saha, S.; Kaur, M.; Bera, J. K. *Organometallics.* **2015**, *34*, 3047–3054. doi:10.1021/acs.organomet.5b00348

[7] Radatz, C. S.; Soares, L. A.; Vieira, E. R.; Alves, D.; Russowsky, D.; Schneider, P. H. *New J. Chem.*, **2014**, *38*, 1410–1417. doi:10.1039/C3NJ01167D

[8] Bhoomireddy, R. P. R.; Narla, L.G. B.; Peddiahgari, V. G. R. *App. Org. Chem.*, **2019**, *3*, e4752. doi:10.1002/aoc.4752

[9] Angelis, S.; Franco, M.; Triminì, A.; Gonzalez, A.; Sainz, R.; Degennaro, L.; Romanazzi, G.; Carlucci, C.; Petrelli, V.; Esperanza, A.; Goñi, A.; Ferritto, R.; Aceña, J. L.; Luisi, R.; Cid, M. B. *Chemistry – An Asian J.*, **2019**, *17*, 3011-3018. doi:10.1002/asia.201900781

[10] Fletcher, J. T.; Keeney, M. E.; Walz, S. E. *Synthesis*. **2010**, *19*, 3339-3345. doi:10.1055/s-0030-1257909

[11] Ladouceur,S.; Soliman, A. M.; Zysman-Colman, E. *Synthesis*, **2011**, *22*, 3604–3611. doi:10.1055/s-0030-1260256

[12] Emilio. J. de la Cerda-Pedro, Amador-Sánchez, Y. A.; Cortés-Hernández, M.; Pérez-Pérez, J.; Rojas-Lima, S.; López-Ruiz, H. *Heterocycles*, **2014**, *1*, 27–41. doi:10.3987/COM-13-12764
